# Behavioral engagement and stimulus regularities coordinate auditory-prefrontal network activity

**DOI:** 10.1371/journal.pbio.3003966

**Published:** 2026-08-26

**Authors:** Haoxuan Xu, Peirun Song, Hangting Ye, Ana Belén Lao-Rodríguez, Qichen Zhang, Yuying Zhai, Xuehui Bao, Ishrat Mehmood, Hisashi Tanigawa, Zhiyi Tu, Lingling Zhang, Xuan Zhao, David Pérez-González, Manuel S. Malmierca, Xiongjie Yu

**Affiliations:** 1 Department of Anesthesia, Women’s Hospital, Zhejiang University School of Medicine, Hangzhou, Zhejiang, China; 2 Department of Anesthesiology, Shanghai Tenth People’s Hospital, Tongji University School of Medicine, Shanghai, China; 3 Zhejiang Provincial Key Laboratory of Precision Diagnosis and Therapy for Major Gynecological Diseases, Women’s Hospital, Zhejiang University School of Medicine, Hangzhou, Zhejiang, China; 4 College of Biomedical Engineering and Instrument Science, Zhejiang University, Hangzhou, China; 5 Cognitive and Auditory Neuroscience Laboratory (CANELAB), Institute of Neuroscience of Castilla y León (INCYL), University of Salamanca, Salamanca, Spain; 6 Institute for Biomedical Research of Salamanca (IBSAL), Salamanca, Spain; 7 Department of Cell Biology and Pathology, Faculty of Medicine, University of Salamanca, Salamanca, Spain; 8 Department of Basic Psychology, Psychobiology and Methodology of Behavioral Sciences, Faculty of Psychology, University of Salamanca, Salamanca, Spain; University College London, UNITED KINGDOM OF GREAT BRITAIN AND NORTHERN IRELAND

## Abstract

The ability to detect deviations from expected sensory input is fundamental for adaptive behavior. We recorded electrocorticographic activity from the auditory (AC) and prefrontal (PFC) cortices of behaving macaques during an auditory oddball task to probe the cortical dynamics of predictive processing. Repetition of standard stimuli evoked suppression and facilitation in AC and strong low-frequency (2 Hz) enhancement in PFC, accompanied by bidirectional delta-band GC-defined interactions indicative of a shared predictive state. Deviant stimuli triggered earlier AC responses followed by PFC activation and increased GC-defined directed interactions across theta, alpha, and conventional gamma bands. Behavioral engagement amplified both repetition-related and deviance-related ECoG responses, strengthening cortical network coordination. Together, these findings reveal behaviorally gated auditory–prefrontal dynamics that are consistent with hierarchical predictive-processing accounts, while also allowing for contributions from repetition-, novelty-, and salience-related mechanisms.

## Abstract 2

How inter-regional coordination supports adaptive responses to behaviorally-relevant stimuli is incompletely understood. This study characterizes the coordination of auditory and prefrontal cortices in context- and behavior-dependent auditory processing, consistent with a predictive coding framework.

## Introduction

Living organisms are continuously exposed to a storm of sensory stimuli, yet only a small fraction is behaviorally meaningful, while most are routine and can be disregarded. Detecting changes or novel events is therefore essential for adaptive behavior, and sensory salience depends strongly on context and prior experience [1–5]. To handle this continuous stream of input efficiently, the brain relies on a proactive computational framework known as predictive coding, which uses prior expectations to guide the processing of incoming sensory information. The predictive coding theory posits that the brain continuously generates active predictions (top-down processing) based on sensory signals from the external environment [6–8]. These predictions are then compared to actual sensory input (bottom-up processing), producing prediction errors that serve as signals to update the system.

The auditory system is particularly efficient at filtering complex acoustic input into manageable representations [9–11]. At the neuronal level, some auditory neurons reduce their firing to repeated sounds while maintaining sensitivity to novel ones, a phenomenon known as stimulus-specific adaptation (SSA) [12–25]. At the macroscopic level, mismatch negativity (MMN), an event-related potential observed in human electroencephalogram (EEG), reflects a similar mechanism of auditory deviance detection [26–30]. SSA and MMN have been extensively investigated using the classical oddball paradigm in animal models, with evidence of their presence across multiple brain regions (including the auditory midbrain and cortex) and across species and arousal states [12–14,16,29,31–34]. Despite these advances, most animal studies have employed acoustically simple, behaviorally irrelevant sounds, leaving key gaps in understanding how prior experience shapes SSA and MMN or how these responses reflect neuronal plasticity [11,18,29,35–37]. Moreover, many findings come from passive conditions in which subjects were not engaged in cognitive or behavioral tasks, highlighting the need to explore SSA and MMN within meaningful behavioral contexts [18,29,38,39]. Non-human primates, whose auditory and cognitive systems closely resemble those of humans, offer an ideal model for such research [40–45]. Macaques, in particular, display human-like auditory abilities including speech discrimination, sound localization and sensitivity to surprising sounds, making them especially suited for investigating the neural mechanisms of change or novelty detection [41,46–48].

SSA and MMN represent neural correlates of prediction errors that intensify along the auditory hierarchy, from the inferior colliculus (IC) to the auditory cortex (AC), and from primary (lemniscal) to higher-order (non-lemniscal) regions [21,34,49,50]. These findings underscore the hierarchical nature of novelty processing but provide limited insight into the interregional, long-range cortico-cortical dynamics underlying prediction and prediction error. It has been proposed that predictions and errors operate in distinct frequency bands—low (alpha/beta oscillations) frequencies for predictions and high (gamma oscillations) frequencies for errors—originating from higher and lower cortical areas, respectively [51–57]. However, direct experimental evidence remains elusive. Testing these hypotheses requires simultaneous recordings across multiple levels of the hierarchy. The AC and prefrontal cortex (PFC) are particularly suitable regions to examine, as both play essential roles in novelty detection yet occupy distinct positions within the cortical hierarchy [31,50,56–60]. Importantly, the PFC is thought to contribute directly to the generation of predictive components [58–62], highlighting the need to determine how top-down signals from the PFC are propagated and integrated with bottom-up inputs from sensory areas. Resolving this interaction remains a central challenge for understanding the neural circuitry that supports the predictive coding framework. Reciprocal anatomical connections between these regions have long been established [63]. In macaques, higher-order auditory cortical fields, including belt and parabelt regions, are anatomically connected to lateral prefrontal regions, providing a structural substrate for auditory–prefrontal interactions [63,64]. Nevertheless, such anatomical connectivity alone does not demonstrate that field-potential coupling reflects direct monosynaptic communication, as both regions may also receive common or indirect sensory-related inputs. Because the MMN typically emerges around 200 ms after a deviant tone, it has been interpreted as a prediction-error response derived from comparing the deviant input with predictions formed from the repeating standards [8]. Human EEG and electrocorticography (ECoG) studies [64,65] show that a network linking the superior temporal gyrus in the temporal lobe with frontal regions is involved in MMN generation. Evidence from non-human primates further supports the view that the prediction-error components underlying MMN are distributed across the temporal and frontal cortices [66–69].

Yet the respective roles of the PFC and AC, and their interactions during predictive processing, remain elusive. These mechanisms are fundamental for adaptive behavior, enabling organisms to anticipate events, detect discrepancies, and update expectations. Monkey ECoG studies have shown that top-down and bottom-up signals propagate through distinct frequency bands [55], and assessing the frequency dependence of phase synchrony between the frontotemporal cortices can reveal the functional coupling that supports prediction and prediction-error responses. ECoG recordings from electrodes implanted in the temporal, lateral prefrontal, and orbitofrontal cortices of macaque monkeys have demonstrated synchronous low-frequency oscillations between frontotemporal regions during both tone presentation and omission [70]. Examining AC–PFC communication, particularly with directional metrics such as Granger causality (GC) [53,61,62], may therefore clarify the circuitry underlying prediction and prediction error. Understanding these processes in behaving non-human primates is essential for advancing our knowledge of cognitive prediction mechanisms.

In this study, we investigated how the auditory and prefrontal cortices jointly implement predictive processing during active listening. Behaving macaques performed a behaviorally relevant auditory oddball task while we recorded electrocorticographic activity simultaneously from both regions. Granger causality analyses revealed the directionality and frequency structure of interareal communication underlying prediction and prediction-error signaling. The two regions showed distinct repetition-dependent dynamics, enhanced low-frequency coupling during auditory regularity, and temporally precise prediction-error responses to deviant sounds. Critically, task engagement strongly amplified these effects, indicating that behavioral relevance sharpens both predictive and error-related representations. Together, these findings uncover a dynamic interplay between auditory and prefrontal cortices that supports context-dependent predictive coding and flexible auditory perception.

## Results

### Repetition-related dynamics during novelty detection in AC and PFC under behavioral conditions

**Fig 1:**
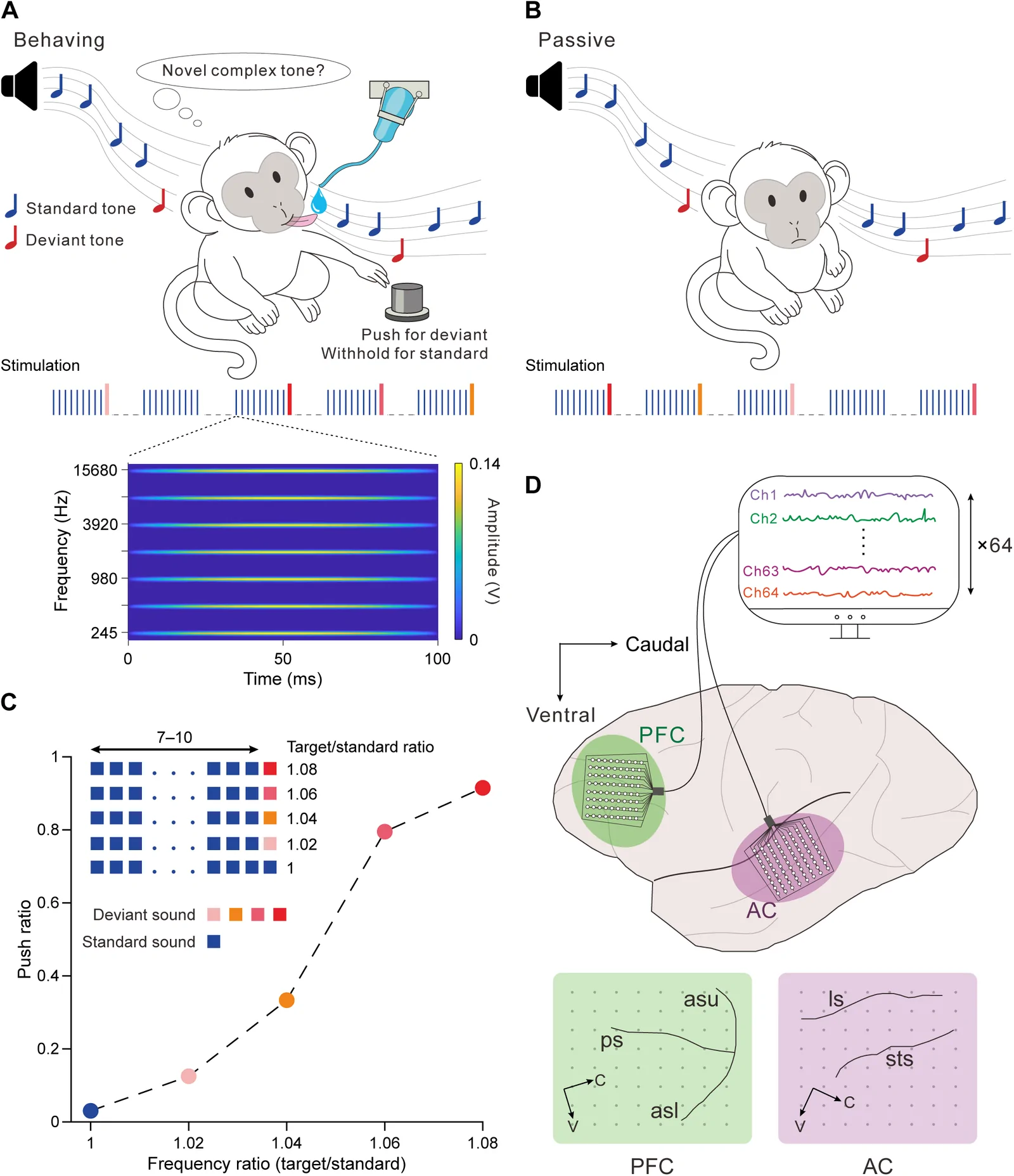
Experimental setup and task design for auditory novelty detection in rhesus monkeys. **(A)** Auditory oddball task during the behaving session. Each trial contained 7–10 repetitions of a standard complex tone followed by a target tone that was either identical to the standard or a frequency deviant. The standard tone had a 245-Hz fundamental frequency with six additional octave-spaced harmonic components. Target/standard frequency ratios were 1 (control), 1.02, 1.04, 1.06, or 1.08. Tones lasted 100 ms and were presented every 500 ms. Monkeys pressed a button within 600 ms after deviant onset and withheld responses for control trials. The lower panel shows the spectrogram and waveform of one complex tone. **(B)** Passive session with the same stimulus sequences but no task engagement. **(C)** Button-push ratio as a function of target/standard frequency ratio, shown for an example session from monkey C. **(D)** Schematic of the two 64-channel ECoG arrays implanted over auditory cortical region and prefrontal cortex in monkey C. AC, auditory cortex; PFC, prefrontal cortex; asl, lower limb of the arcuate sulcus; asu, upper limb of the arcuate sulcus; ps, principal sulcus; ls, lateral sulcus; sts, superior temporal sulcus; C, caudal; V, ventral.

To probe the neural and behavioral mechanisms of novelty detection, we trained two macaques to perform an auditory oddball task composed of sequential blocks of stimuli (Fig 1A). Each block contained 7–10 repetitions of complex sounds composed of seven octave-spaced frequency components with a 245-Hz fundamental frequency (Fig 1A bottom, see Materials and methods for details). Each block ended with either a standard stimulus, identical to preceding ones, or a frequency deviant (Fig 1A). Monkeys were required to press a button within 600 ms after deviants (hit trials) and to withhold responses to standards (correct rejections). A separate passive session was conducted after the behavioral session, during which the same oddball stimuli were presented without task engagement (Fig 1B). Behavioral accuracy increased with the magnitude of frequency contrast or deviation (Fig 1C). Neural activity was simultaneously recorded from two 64-channel ECoG arrays implanted over the temporal auditory region and sampled lateral prefrontal cortex (Figs 1D and S1 show electrode coverage and visible anatomical landmarks, including the superior temporal sulcus and lateral sulcus). Because some temporal-array contacts were located outside the likely auditory cortical territory, AC analyses were restricted to channels located within the lateral sulcus–superior temporal sulcus region and to adjacent contacts showing significant auditory responses (S2 Fig; Materials and methods).

**Fig 2:**
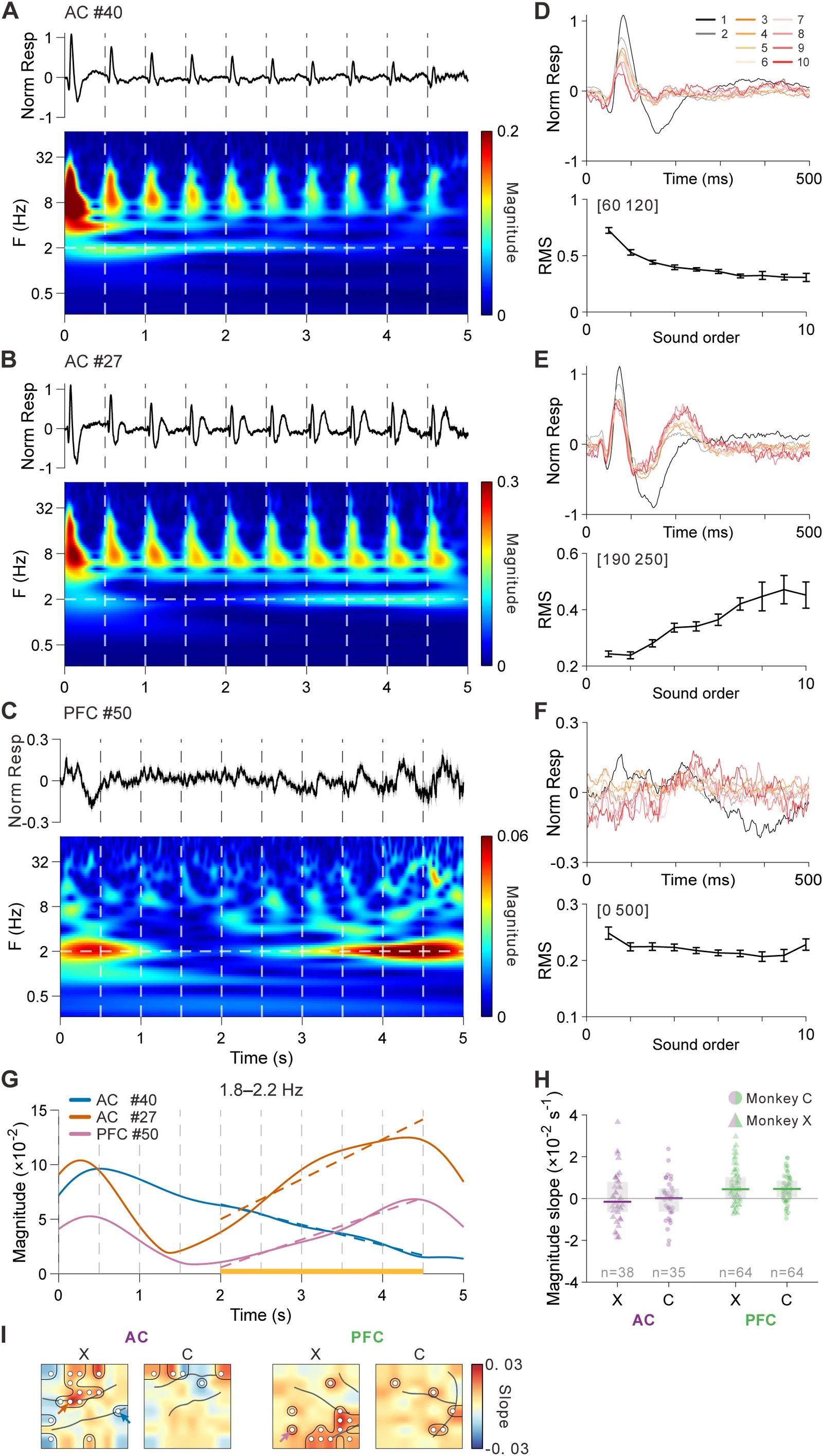
Repetition-related suppression and enhancement in AC and PFC activity. **(A)** Example AC channel (#40, monkey X) showing repetition-related suppression. Top, trial-averaged normalized response; bottom, corresponding time–frequency magnitude. Vertical dashed lines indicate standard-tone onsets, and the horizontal dashed line marks 2 Hz, corresponding to the 500-ms onset-to-onset interval. **(B, C)** Example AC (#27) and PFC (#50) channels from monkey X showing repetition-related enhancement. **(D–F)** Responses to standard tones 1–10 for the example channels. Top, normalized responses by sound order; bottom, RMS response magnitude measured within the indicated time window. **(G)** Time course of magnitude in the 1.8–2.2 Hz band for the three example channels. Vertical black dashed lines indicate the onset of sounds. Slanted dashed lines represent linear fits from 2 to 4.5 s (corresponding to orders 5 to 9, indicated by the horizontal yellow line). The endpoint at 4.5 s marks the onset of the 10th standard and was used only as the boundary of the 9th-standard response window. **(H)** Dot plots of 1.8–2.2 Hz magnitude slopes across included channels in the AC and PFC arrays of both monkeys. Each dot represents one channel. Horizontal lines indicate the median, and vertical gray bars indicate the interquartile range (25th–75th percentile). Numbers indicate the channels included in each group. **(I)** Topographic maps of 1.8–2.2 Hz magnitude slopes for the AC and PFC arrays in monkeys X and C. Colored arrows indicate the three example channels shown in **(G)**.

We first examined neural responses to repeated standard tones and identified two distinct adaptation profiles. In the first, exemplified by an AC site, strong responses to the initial tone progressively attenuated across repetitions (Fig 2A, top). This site showed continued attenuation through the 10th repetition, consistent with the phenomenon of *repetition suppression*, a reduction in neural response under high certainty of repeated input [71]. Time-frequency analysis revealed parallel decreases in magnitude within the alpha band (8–14 Hz) and around 2 Hz (Fig 2A, bottom). In contrast, a second response type exhibited progressive enhancement in amplitude from the third to 10th tone (Fig 2B, top), accompanied by rising ~2 Hz magnitude aligned with the stimulus rhythm (Fig 2B, bottom). This pattern suggests the phenomenon of *repetition enhancement*, reflecting the buildup of temporal expectations or short-term memory of the tone sequence [72]. Finally, a representative PFC site showed minimal responses to early repetitions (3–6) but strong activation during later ones (8–10), paralleled by increased ~2 Hz magnitude (Fig 2C), indicating that repetition-related low-frequency dynamics also emerged in frontal regions.

To further characterize repetition-related dynamics, we aligned neural responses by presentation order. In the example AC site, evoked activity around 100 ms after tone onset progressively declined with repetition (Fig 2D). In contrast, a second AC site showed increasing responses centered near 220 ms (Fig 2E), whereas a PFC site showed a gradual buildup of activity across the 500-ms inter-stimulus interval (Fig 2F). These divergent profiles reveal systematic, site-specific modulation by repetition—decreasing activity in one AC site, increasing in another, and broad repetition-related enhancement in PFC. Corresponding time–frequency maps showed parallel trends, particularly within the 1.8–2.2 Hz range (bottom rows in Fig 2A–2C).

We quantified these effects by tracking 2-Hz-band magnitude, defined as the mean magnitude within 1.8–2.2 Hz, aligned with the 2-Hz stimulation rhythm corresponding to the 500-ms ISI (Fig 2G). Linear fits (dashed lines, Fig 2G) across the 5th–9th tones revealed a negative slope for the first AC site, consistent with repetition suppression, and positive slopes for the second AC and PFC sites, consistent with repetition enhancement. Across the anatomically and functionally defined AC channels, 49.3% showed increasing 2-Hz-band magnitude over repetitions, consistent with repetition enhancement, whereas 50.7% showed decreasing 2-Hz-band magnitude, consistent with repetition suppression. In PFC, 75.8% of channels showed increasing 2-Hz-band magnitude and 24.2% showed decreasing 2-Hz-band magnitude over repetitions (Fig 2H). The average slope of 2-Hz-band magnitude was significantly steeper in PFC than in AC (*F*(1,197) = 16.66, *p* < 0.001, *η*² = 0.08; two-way ANOVA), indicating stronger repetition-related low-frequency modulation in PFC. Together, these results show that repetition enhancement is particularly prominent in prefrontal regions, where it is reflected in the progressive buildup of 2-Hz repetition-related ECoG activity. A separate 400-ms ISI condition showed comparable repetition-related slope analyses centered on the 2.5-Hz stimulation rate, providing an additional control for the repetition-related magnitude analysis (S3 Fig).

**Fig 3:**
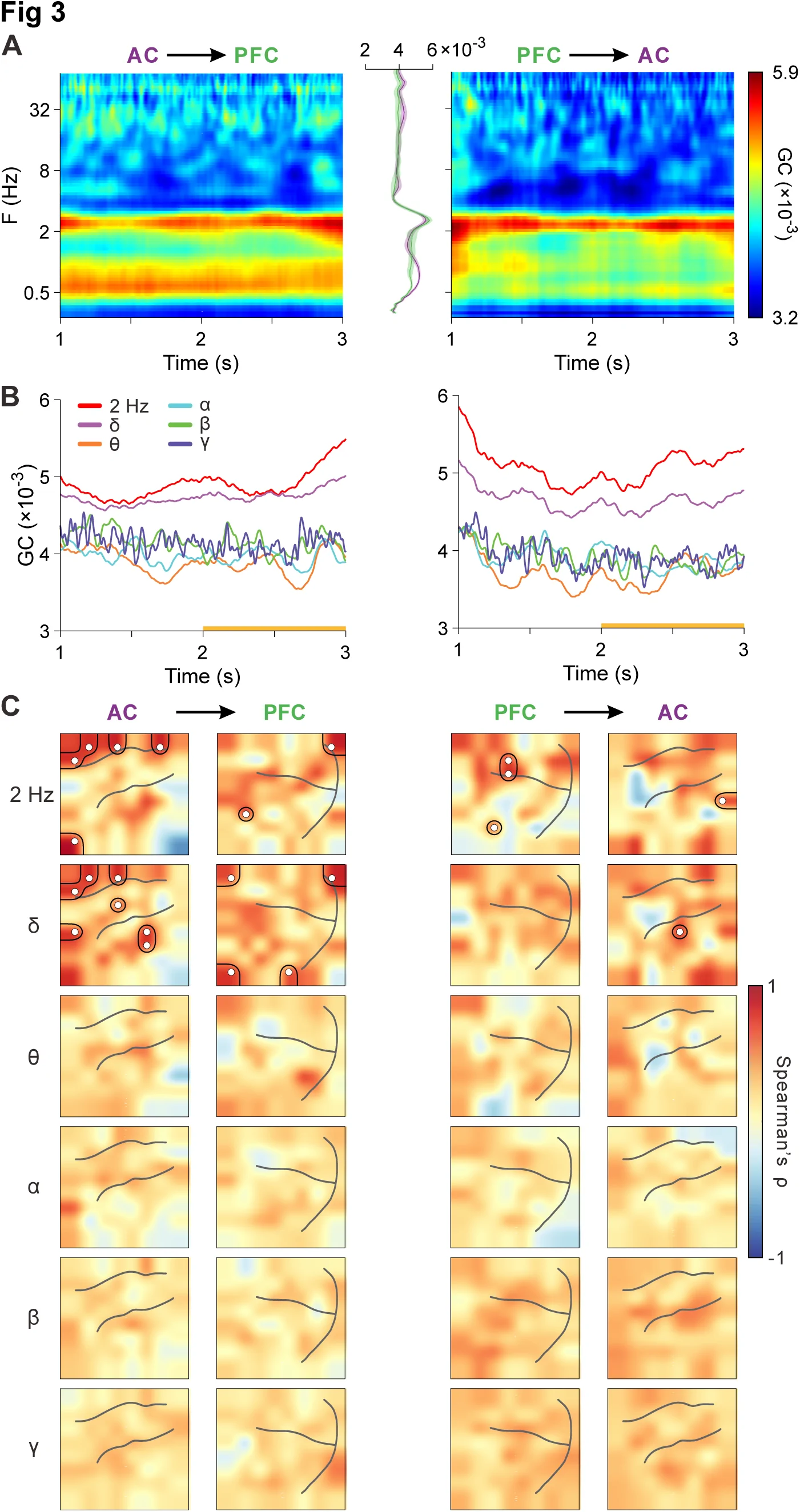
Enhancement of 2-Hz GC-defined interactions between AC and PFC during standard repetition. **(A)** Time-frequency maps of Granger Causality (GC) spectrum, aligned to the onset of the first standard sound and averaged across AC–PFC channel pairs in monkey C. For visualization, only the later standard-repetition period from 1 to 3 s after sequence onset is shown; because the ISI was 500 ms, this displayed window begins at the onset of the third standard sound. The example GC was computed using trials with a standard sound number of 8. Traces on the right and left display the GC averaged across time from 1 to 3 s, with a peak around 2 Hz. **(B)** GC for different frequency bands as a function of time, averaged across AC–PFC channel pairs for monkey C in both directions. Horizontal yellow bars indicate time window (2–3 s, orders 5–6) used for temporal-correlation analysis. **(C)** Topographic maps of temporal correlations in GC for each frequency band and direction in monkey C. Circled channels indicate significant correlations (Spearman’s *ρ*, *p* < 0.05, FDR-corrected across channels).

After characterizing local responses to standard stimuli, we next examined large-scale interactions between AC and PFC during the repetition period using Granger causality analysis. We focused on the 1–3 s window of each tone block corresponding to the later repetition period beginning at the third standard tone, and averaged GC estimates across pairs between the anatomically and functionally defined AC contacts and PFC contacts (Fig 3A). GC spectra revealed a prominent ~2 Hz peak in both directions (AC → PFC and PFC → AC) indicating bidirectional GC-defined interactions near the stimulus repetition rate.

To assess frequency-specific GC-defined interactions, we computed GC across the 2-Hz band (1.8–2.2 Hz) and canonical frequency bands (delta: 0.6–4 Hz; theta: 4–8 Hz; alpha: 8–14 Hz; beta: 14–30 Hz; gamma: 30–70 Hz) (Fig 3B). In both the AC → PFC and PFC → AC directions, GC-defined interactions were strongest at 2 Hz and within the delta range, with minimal coupling at higher frequencies. Temporal correlations of GC (Spearman, 2–3 s window) confirmed that significant, stable interactions emerged exclusively in the 2 Hz and delta bands (Fig 3C). These effects were consistent across animals (Figs 3C and S4). Notably, when the repetition rate was increased from 2 to 2.5 Hz using a 400-ms ISI, GC peaks remained near ~2 Hz rather than shifting to the 2.5-Hz stimulation rate (S5A Fig). This observation suggests that low-frequency AC–PFC coupling reflects more than direct tracking of the external stimulus rhythm, although sensory tracking, temporal alignment to the repeated acoustic stream, and other sequence-related low-frequency processes may contribute to the observed effects.

### Deviance-related responses during novelty detection in AC and PFC under behavioral conditions

**Fig 4:**
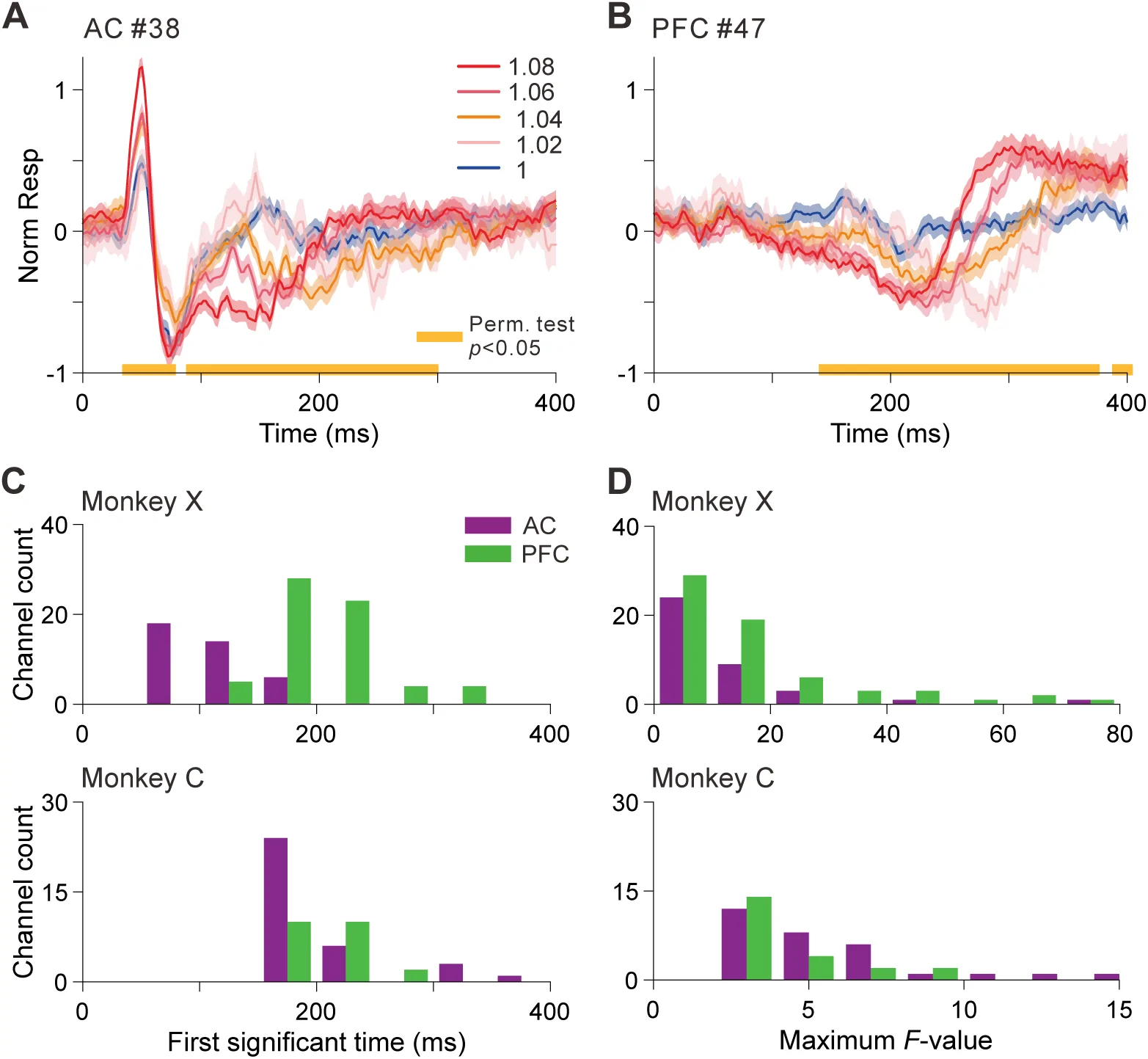
Deviance-related responses in the AC array and PFC. **(A)** Normalized responses of an example AC channel (#38, monkey X) to the final sound, plotted separately for target/standard frequency ratios of 1, 1.02, 1.04, 1.06, and 1.08. Shaded areas indicate ±s.e.m. Yellow bars at the bottom indicate time points with significant response differences across ratios (cluster-based permutation test, *p* < 0.05, corrected). **(B)** Same as (A) for an example PFC-array channel (#47, monkey X). **(C)** Distributions of the first significant time across AC and PFC channels showing significant deviance-related responses within 0–350 ms after final-sound onset for each monkey. **(D)** Distributions of the maximum *F*-value within 0–350 ms after final-sound onset across the same significant channels shown in (C).

Next, we analyzed ECoG responses to deviant tones in both AC and PFC. An example AC site showed robust deviant responses whose magnitude scaled with the frequency difference from standards (Fig 4A). In contrast, a representative PFC site exhibited minimal activity to standards but strong activation to deviants (Fig 4B). To quantify these effects, we extracted two measures: (1) the first significant time, defined as the earliest time bin showing a significant response difference across target/standard frequency ratios in a cluster-based permutation test, and (2) the maximum *F*-value within 0–350 ms after final-sound onset, indexing deviance-related response strength. In the illustrated AC site, the first significant time occurred at 38 ms with a maximum *F*-value of 20.93, whereas in the PFC site, significance emerged later at 144 ms with a peak *F*-value of 14.27.

At the population level, deviance-related response differences emerged reliably earlier in AC than in PFC among channels showing significant deviance-related responses within 0–350 ms after final-sound onset for both monkeys (Fig 4C; monkey X: |*Z|* = 7.83, *p* < 0.001, *r* = 0.78; monkey C: |*Z|* = 3.14, *p* < 0.01, *r* = 0.42; two-tailed Mann–Whitney *U* test, AC versus PFC). However, absolute latency distributions varied across animals, with Monkey C exhibiting generally later and more variable response onsets, including within AC. Accordingly, we interpret these results as evidence for a relative AC-before-PFC temporal sequence within each animal, rather than for a fixed absolute latency of deviance detection across animals. In contrast, maximum *F*-values among these significant channels did not differ significantly between regions (Fig 4D; monkey X: |*Z|* = 1.19, *p* = 0.24, *r* = 0.12; monkey C: |*Z|* = 1.57, *p* = 0.12, *r* = 0.22). Sorting channels by first significant time further confirmed earlier deviance-related responses in AC (S6A and S6B Fig). The channel-by-time *F*-value maps also illustrated inter-animal variability in the timing and distribution of deviance-related responses (S6A and S6B Fig).

We further examined local time–frequency responses time-locked to the onset of the final sound. For each deviant ratio (1.02, 1.04, 1.06, and 1.08), local time-frequency magnitude was compared with the standard condition by computing deviant-minus-standard ΔMagnitude in AC and PFC. The same example channels shown in Fig 4A and 4B exhibited deviance-related time-frequency magnitude changes after final-sound onset (S7 Fig). Band-specific quantification further showed positive deviant-minus-standard magnitude changes across multiple frequency bands and deviant ratios in both regions, consistent with the ratio-dependent deviance-related responses shown in Fig 4. These local time–frequency analyses further characterize deviance-related ECoG activity before turning to the GC-defined AC–PFC interaction analyses.

**Fig 5:**
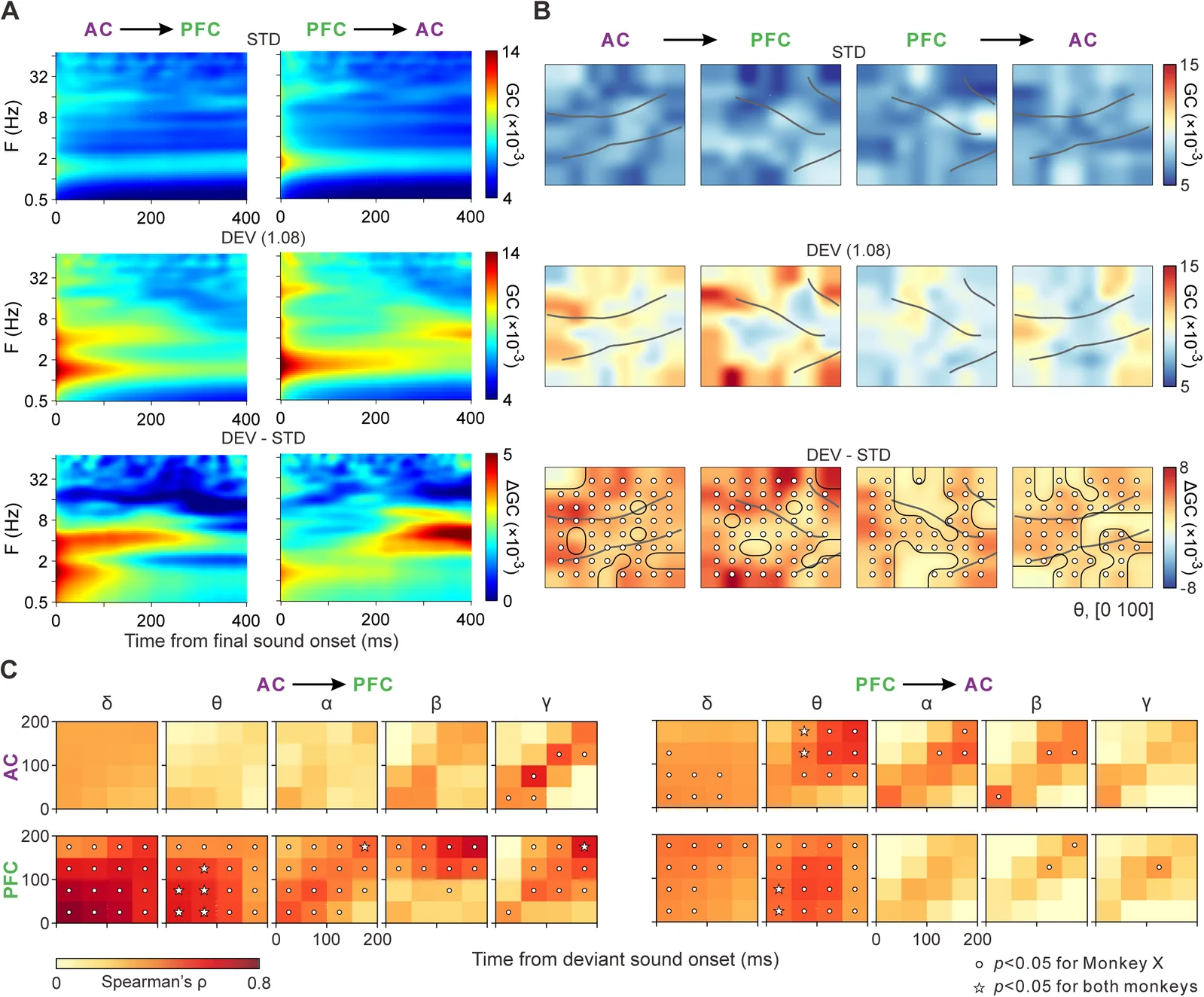
Deviance-related GC-defined interactions between AC and PFC. **(A)** Time-frequency maps of bidirectional GC between AC and PFC in monkey X, aligned to the onset of the final sound and averaged across AC–PFC channel pairs. Left, AC → PFC; right, PFC → AC. Rows show GC for the standard condition, the deviant condition with a target/standard frequency ratio of 1.08, and the differential GC map (deviant minus standard). **(B)** Topographic GC maps for the standard, deviant, and differential conditions, averaged in the theta band within 0–100 ms after final-sound onset. Maps are shown for both cortical regions and both directions. Open circles indicate channels with significantly larger GC for deviant than standard trials (one-tailed permutation test, *p* < 0.05, FDR-corrected across channels). **(C)** Time-by-time cross-condition correlation matrices of deviance-related differential GC topographies for monkey X. Differential GC was defined as deviant minus standard GC. Matrices show the mean Spearman’s *ρ* across deviant-condition pairs (1.04, 1.06, and 1.08), computed separately for each frequency band, direction, and cortical map. Dots indicate significant correlations in monkey X, and stars indicate significant correlations in both monkeys (*p* < 0.05, uncorrected; see Materials and methods).

To probe GC-defined interactions during deviant processing, we applied Granger causality analysis. Time-frequency GC spectra (aligned to final-sound onset and averaged across AC–PFC channel pairs in monkey X) revealed weak GC-defined directed interactions during standards (Fig 5A, top) but a marked GC increase for deviants (1.08 frequency ratio; Fig 5A, middle). This enhancement emerged within 100 ms, dominated by delta/theta GC increases (0.6–8 Hz, AC → PFC), and shifted to theta/alpha frequencies (4–14 Hz, PFC → AC) around 200–300 ms (Fig 5A, bottom), consistent with rapid, frequency-specific engagement of the AC–PFC network during deviance-related processing.

Topographic GC maps corroborated these effects (Fig 5B): bidirectional GC-defined interactions were minimal for standards but rose sharply for deviants, particularly in the AC → PFC direction, with subtraction maps showing larger deviance-related increases in this direction. Across frequency bands, deviant stimuli elicited significant GC-defined directed interactions from delta to gamma ranges in both directions and across both monkeys (S8 Fig). Temporal correlation matrices of differential GC maps across frequency-ratio pairs (1.04 versus 1.06, 1.04 versus 1.08, 1.06 versus 1.08) further showed consistent AC → PFC patterns in the theta/alpha (4–14 Hz) and conventional gamma (30–70 Hz) bands, while PFC → AC patterns predominated in the theta (4–8 Hz) range (Fig 5C). These effects were reproduced in the second monkey (S9 Fig), consistent with frequency-specific, bidirectional ECoG-level coordination between auditory and prefrontal cortices during deviant detection.

### Active behavioral states amplify repetition-related and deviance-related dynamics in AC and PFC

**Fig 6:**
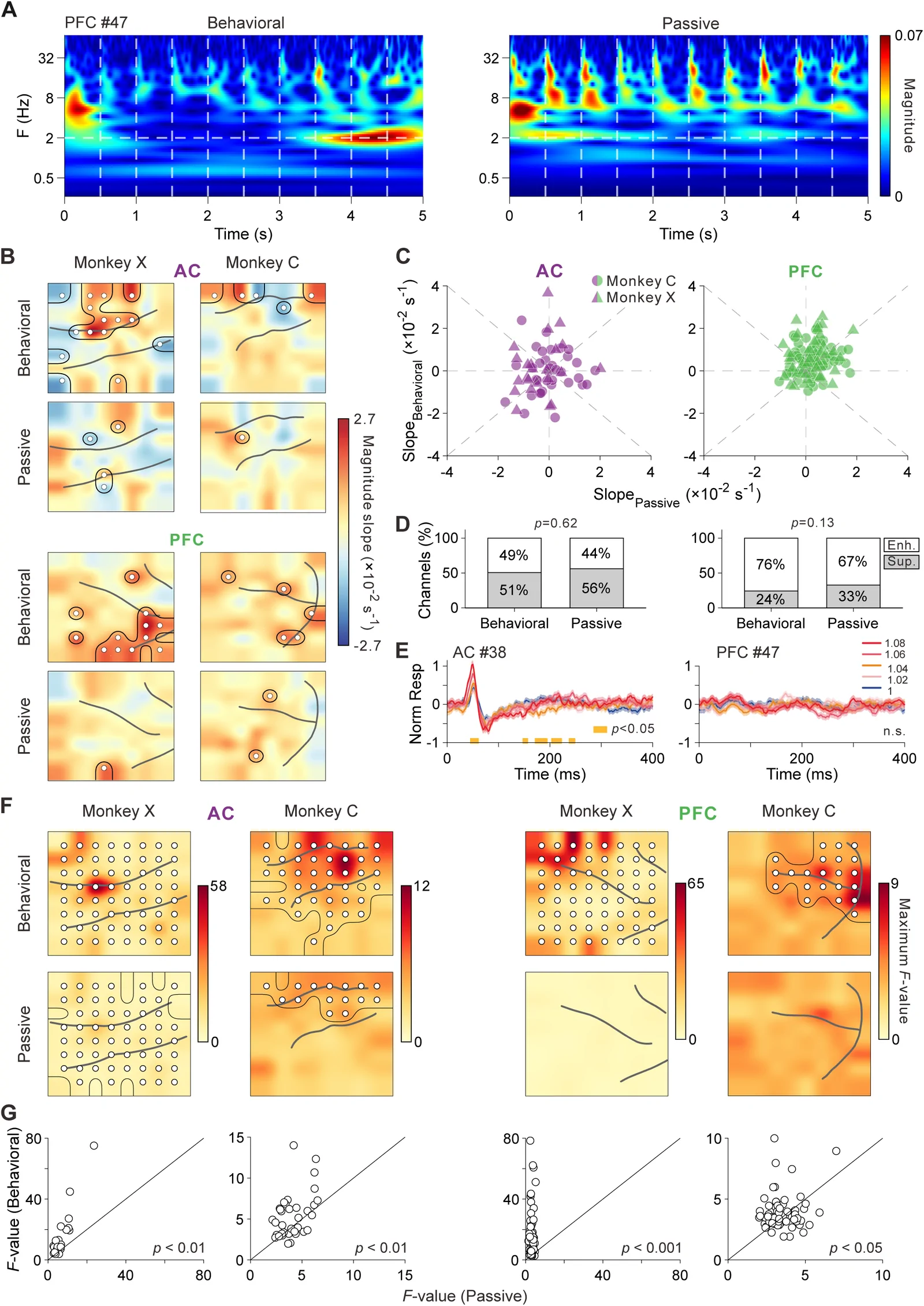
Behavioral engagement modulates repetition-related and deviance-related responses. **(A)** Time-frequency magnitude of an example PFC channel (#47, monkey X) during repeated standard presentation under behavioral and passive conditions. **(B)** Topographic maps of 1.8–2.2 Hz magnitude slopes across repeated standard sounds in AC and PFC under both conditions. **(C)** Channel-wise comparison of 1.8–2.2 Hz magnitude slopes between behavioral and passive conditions. Each point represents one channel; data from both monkeys are pooled. **(D)** Proportions of channels showing repetition enhancement or repetition suppression under behavioral and passive conditions. Channels were classified according to the sign of the 1.8–2.2 Hz magnitude slope. Positive and negative slopes were classified as repetition enhancement and suppression, respectively. Stacked bars show the percentages of enhancement and suppression channels pooled across monkeys. p-values shown above the bars indicate exact McNemar tests comparing behavioral and passive conditions. Enh., enhancement; Sup., suppression. **(E)** Normalized responses of example AC and PFC channels from monkey X under the passive condition, aligned to final-sound onset and plotted for target/standard frequency ratios of 1, 1.02, 1.04, 1.06, and 1.08. Yellow bars indicate significant response differences across ratios (cluster-based permutation test, *p* < 0.05, corrected). **(F)** Topographic maps of the maximum *F*-values within 0–350 ms after final-sound onset for AC and PFC in both monkeys. Open circles indicate channels with significant deviance-related responses within this window. **(G)** Channel-wise comparison of maximum *F*-values between behavioral and passive conditions for each area and monkey. *p*-values indicate two-tailed paired *t*-*t*ests.

In the previous sections, we characterized how repetition-related and deviance-related activity emerged during active behavioral engagement. However, to determine whether these effects required task involvement or instead reflected more general auditory processing, it was essential to examine them under passive listening as well (Fig 1B). Therefore, we next compared neural responses during passive presentation of the auditory sequences with those recorded while monkeys performed the active novelty-detection task, allowing us to assess how behavioral engagement shaped repetition-related and deviance-related processing. We first examined repetition-related processing. In the behavioral condition (Fig 1A), a representative PFC site displayed robust ~2 Hz repetition-related activity between 3.5 and 5 s (Fig 6A, left), which was markedly reduced during passive listening (Fig 6A, right). To assess regional specificity, we compared the included AC contacts and PFC contacts (Fig 6B). Behavioral modulation of repetition-related activity was stronger in PFC than in AC, as shown by site-by-site scatter plots (Fig 6C). Statistical analysis confirmed a significant behavioral effect in PFC (*F*(1,126) = 14.83, *p* < 0.001, *η*² = 0.11; Fig 6C, right), but not in AC (*F*(1,71) = 0.34, *p* = 0.56, *η*² = 0.01; Fig 6C, left). We further quantified the balance between repetition enhancement and repetition suppression by classifying each channel according to the sign of the 2-Hz-band magnitude slope. Channels with positive slopes were defined as showing repetition enhancement, whereas channels with negative slopes were defined as showing repetition suppression. This analysis showed a numerically higher proportion of enhancement channels during active behavior, particularly in PFC, although the categorical enhancement/suppression balance did not differ significantly between behavioral states (Fig 6D). In AC, enhancement channels accounted for 49.3% during active behavior and 43.8% during passive listening; in PFC, enhancement channels accounted for 75.8% during active behavior and 67.2% during passive listening. Thus, behavioral engagement increased the magnitude of repetition-related low-frequency modulation, especially in PFC, while producing only a modest, non-significant change in the proportion of channels classified as repetition enhancement versus suppression. Granger causality analysis further revealed weaker AC–PFC GC-defined interactions during passive listening (S10A Fig) compared with active engagement (Fig 3A). Unlike the pronounced bidirectional dynamics observed during task performance, temporal modulation of GC-defined interactions was largely absent under passive conditions (S10B Fig).

Next, we examined deviance-related responses. Example sites in both AC and PFC (Fig 6E, left and right, respectively) showed markedly reduced ratio-dependent response differentiation during passive listening, compared with the clear response separation observed during active task performance (*cf.*, Fig 4A and 4B). Across both regions and animals, the spatial distribution of *F*-values was attenuated in the passive condition (Fig 6F). Site-wise comparisons confirmed significantly stronger deviance-related responses during active behavior (*p* < 0.05 for all comparisons; two-tailed paired *t t*est; Fig 6G). Granger causality analysis further revealed stable GC-defined AC → PFC interactions in the theta and alpha bands during passive listening, indicating that deviance-related auditory–prefrontal coupling was still detectable even without active behavioral engagement (S11 Fig).

## Discussion

**Fig 7:**
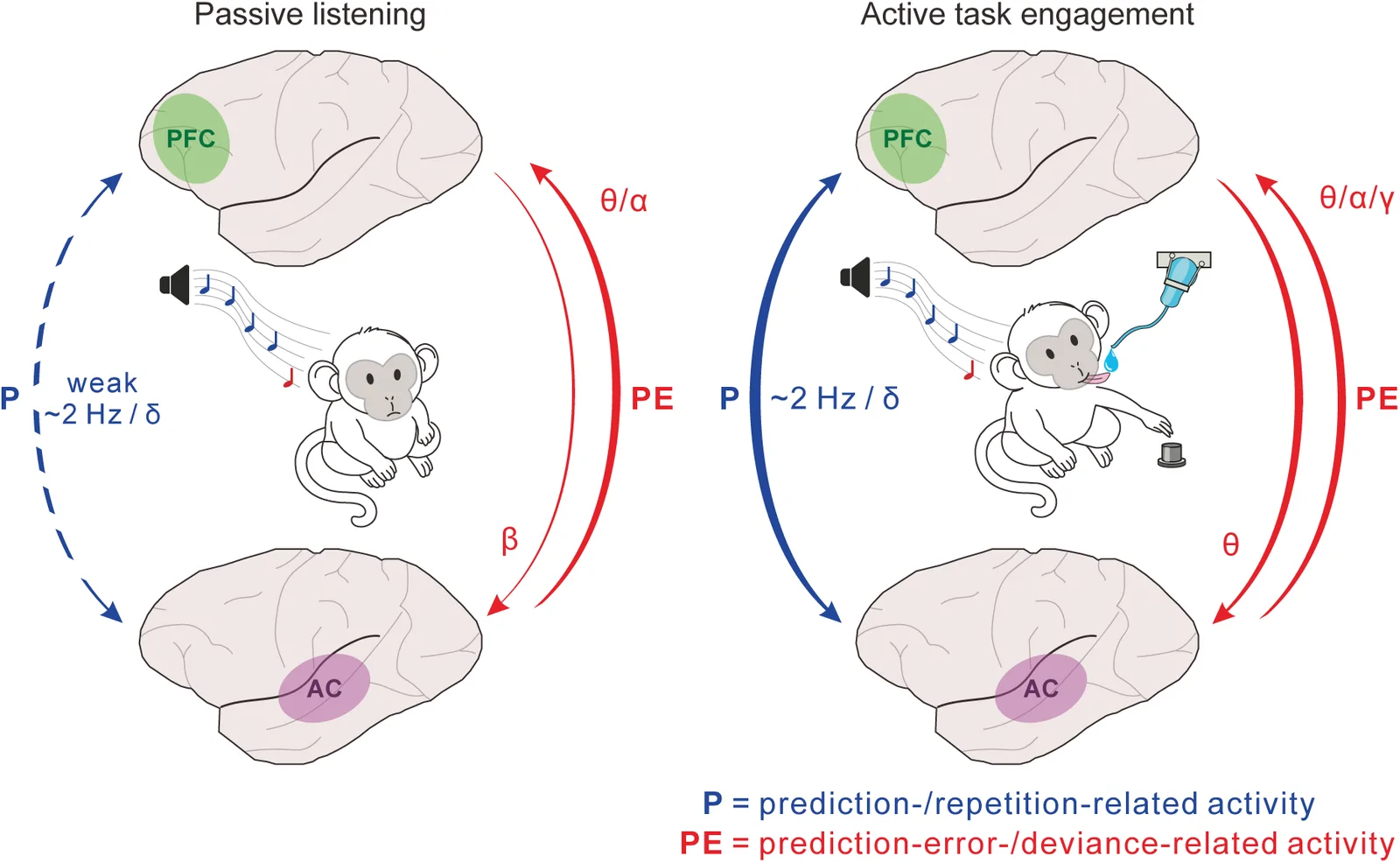
State-dependent auditory–prefrontal dynamics during novelty detection. Schematic summary of the main findings. During standard repetition, prediction-/repetition-related activity (P, blue) was associated with low-frequency GC-defined interactions between AC and PFC, mainly around the ~2-Hz/delta range. These interactions were weak during passive listening and stronger during active task engagement. During deviant processing, prediction-error-/deviance-related activity (PE, red) was associated with frequency-specific bidirectional GC-defined interactions. Under passive listening, AC → PFC interactions were detectable mainly in theta/alpha bands, with limited beta-band PFC → AC patterns. Under active task engagement, AC → PFC interactions were more prominent in theta/alpha and conventional gamma bands, whereas PFC → AC interactions were more evident in the theta range. Dashed arrows indicate weak or limited GC-defined interactions, and solid arrows indicate stronger or more consistent interactions. AC, auditory cortex; PFC, sampled lateral prefrontal cortex; δ, delta; θ, theta; α, alpha; β, beta; γ, conventional gamma band.

In this study, we examined how auditory cortex and prefrontal ECoG networks cooperate to predict and detect sensory novelty in behaving macaques. Repetition of standard tones produced two distinct response profiles: while some AC sites showed repetition suppression, a subset of AC sites and most PFC sites exhibited repetition enhancement, dominated by low-frequency (~2 Hz) repetition-related ECoG activity (Fig 2). Linear fits of 2-Hz-band magnitude confirmed stronger facilitation in PFC than in AC (Fig 2H). Granger causality analyses revealed robust bidirectional GC-defined interactions between the two cortical regions, peaking near 2 Hz within the delta band, consistent with coordinated predictive signaling (Figs 3, S4, and S5). Deviant tones elicited early responses in AC and later activation in PFC (Fig 4A–4C), with comparable deviance-related response strength across regions (Fig 4D). Deviance processing further involved frequency-specific GC-defined interactions, including AC → PFC theta/alpha and conventional gamma-band patterns and PFC → AC theta patterns (Figs 5C, S8, and S9). Importantly, behavioral engagement amplified local repetition-related and deviance-related responses and shaped AC–PFC GC-defined interactions (Figs 6, S10, and S11). Together, these results reveal a distributed, state-dependent auditory–prefrontal network in which auditory-region contacts capture early sensory regularities and violations, whereas PFC activity reflects behavioral context and recurrent network modulation. These findings are consistent with a predictive-coding interpretation, while also allowing alternative explanations based on adaptation, novelty detection, salience, and task-dependent gain modulation (Fig 7).

An important limitation is that the present oddball paradigm design cannot, by itself, distinguish predictive processing from other context-dependent mechanisms. Repetition suppression may reflect reduced prediction error under a predictive-coding account, but it may also arise from stimulus-specific adaptation, synaptic or network-level fatigue, or reduced responsiveness to repeated acoustic input. Likewise, deviance-related responses can be interpreted as prediction-error-related activity, but they may also reflect novelty detection, sensory salience, frequency contrast, or task-dependent gain modulation [7,71,73]. We therefore use predictive coding as an interpretive framework rather than as a mechanism directly proven by the present data. The central empirical finding is that repetition-related and deviance-related ECoG responses, together with AC–PFC GC-defined interactions, are modulated by behavioral engagement.

### Dual modes of repetition-related modulation in the AC–PFC circuit

Repetition suppression and repetition enhancement represent complementary forms of repetition-related modulation within the auditory–prefrontal network. In the anatomically and functionally defined AC channels, repetition suppression (observed in 50.7% of electrodes) appeared as a gradual attenuation of evoked responses to repeated tones (Fig 2A and 2D), consistent with predictive coding models in which redundant input reduces prediction-error signaling in primary auditory and subcortical nuclei [4,5,71,74]. A comparable subset of AC channels exhibited pronounced repetition enhancement, with evoked amplitudes and phase-locked magnitude at the stimulation rhythm (~2 Hz) increasing monotonically across repetitions (Fig 2B and 2E). Similar facilitation reported in marmoset and mouse AC [75,76] may represent a transient “prediction template” that sharpens temporal expectancy through delta-phase alignment [77]. The PFC showed the same dual motifs but was strongly biased toward enhancement: 75.8% of electrodes displayed progressive facilitation, typically with a later buildup than in AC (Fig 2C and 2F–2H). This predominance accords with evidence that prefrontal regions can broadcast precision-weighted predictions to increase auditory gain under high behavioral relevance [50,78] and sustain cross-modal predictive templates during working memory [78].

Interestingly, these repetition-related effects were strongly modulated by behavioral state. During passive listening, the average 2-Hz-band magnitude slope was significantly lower in PFC than in the active condition (55.2% reduction), whereas AC showed no significant overall change, reflecting a more balanced distribution of enhancement and suppression effects across contacts. Consistent with this pattern, low-frequency AC–PFC GC-defined interactions were also weaker during passive listening (S10 Fig). This pattern mirrors evidence that task engagement amplifies frontal contributions to sensory processing [79,80], suggesting that PFC repetition enhancement is more strongly engaged during active behavior. However, the categorical balance between repetition enhancement and suppression showed only a modest, non-significant shift between behavioral states: enhancement channels decreased from 49.3% to 43.8% in AC and from 75.8% to 67.2% in PFC during passive listening. Thus, passive listening reduced the strength of repetition-related enhancement, especially in PFC, but did not produce a significant categorical reorganization of enhancement versus suppression. This state dependence highlights a functional dissociation between repetition suppression and repetition enhancement within the AC–PFC circuit: repetition suppression may optimize coding efficiency for predictable repetitive stimuli, whereas repetition enhancement may strengthen the representation of behaviorally relevant information through active cortical buffering.

Converging human magnetoencephalogram (MEG) and functional magnetic resonance (fMRI) work suggests that such dual repetition effects are a general feature of auditory memory-trace and prediction formation. Using a roving-standard paradigm, a human MEG study [81] has shown that repetition suppression of the N1m response is accompanied by a progressive repetition enhancement of a later sustained field, and that both components contribute to the build-up of the repetition positivity [72] and the echoic memory trace. Another event-related fMRI study [82] further demonstrated that deviant sounds preceded by a longer sequence of standard tones evoke stronger blood-oxygen level-dependent responses in bilateral auditory cortex, and that responses to the standards themselves show a mixture of suppression and enhancement depending on whether they predict an upcoming deviant. These findings support the view that repetition suppression primarily reflects reduced prediction error for highly expected input, whereas repetition enhancement may index the strengthening of short-term auditory predictions and sensory memory representations in auditory cortex.

Our macaque ECoG data extend this perspective in several important ways. First, we show that repetition suppression and enhancement coexist not only within AC but also in PFC, and that their relative expression is modulated by behavioral state, with enhancement particularly prominent in PFC during active task engagement. Second, the slow build-up of low-frequency activity we observe in PFC—and in a subset of AC sites—resembles the sustained-field repetition enhancement and deviant-history effects described in human MEG and fMRI, suggesting a common mechanism whereby frontal feedback and local delta-band entrainment maintain short-term auditory predictions during regular stimulation [81,82]. Third, by combining these repetition effects with GC-defined interaction analyses, we show that they are embedded in a structured AC–PFC network, in which PFC feedback preferentially amplifies enhancement-related dynamics under behavioral demands. This cross-species convergence supports the notion that dual repetition mechanisms—suppression and enhancement—are a core implementation of predictive coding in the auditory system, jointly supporting efficient encoding of predictable input and robust maintenance of context-dependent expectations.

The coexistence of repetition suppression and repetition enhancement within the same regions supports a flexible predictive coding framework. Repetition suppression in AC may reflect prediction error minimization at early sensory stages, whereas repetition enhancement in PFC may index precision-weighted model updating [71]. Repetition-rate magnitude changes in AC (Fig 2) and task-dependent facilitation in PFC (Fig 6) suggest that temporal coding and fronto-sensory interactions jointly refine predictive accuracy. Together, these mechanisms show how the brain balances efficiency through suppression and precision through enhancement, adapting dynamically to behavioral demands via repetition suppression, repetition enhancement, and AC–PFC coordination. Future work should examine how these processes generalize to naturalistic contexts and to clinical conditions with impaired predictive processing.

The GC patterns observed during standard stimulus repetitions revealed a structured interplay between AC and PFC that supports predictive maintenance. Bidirectional GC-defined interactions peaked near 2-Hz repetition-rate band in the 500-ms ISI condition (Fig 3A and 3B), matching the stimulation rhythm and phase-locked delta oscillations in both regions (Fig 2G and 2H). These low-frequency interactions suggest that AC and PFC synchronize to encode temporal regularities, with reciprocal AC → PFC and PFC → AC signaling reinforcing the stability of predictive templates. The dominance of delta frequencies in these exchanges (Fig 3B and 3C) aligns with theories positing that low-frequency oscillations coordinate hierarchical predictions [51,77,83], whereby AC conveys sensory-derived temporal structure to PFC, which in turn modulates auditory gain to emphasize behaviorally relevant patterns [50]. More broadly, this interpretation is also consistent with cross-species evidence that frontal–auditory coupling is not unique to macaques or humans. In bats, simultaneous recordings from frontal–auditory field and auditory cortex have revealed robust fronto-temporal coupling during spontaneous activity and auditory processing, as well as behavior-dependent reversals of directed functional interactions between frontal and auditory cortices during vocal behavior [84,85]. In rodents, orbitofrontal cortex modulates auditory cortical sensitivity and sound perception, supporting the idea that frontal regions can shape auditory cortical processing according to behavioral context [86]. In marmosets, frontal–auditory cortical interactions during vocal production have been linked to sensory prediction and feedback-dependent control [87]. Together, these studies suggest that frontal–auditory coordination may represent an evolutionarily conserved circuit motif for linking auditory processing with behavioral context.

The buildup of ~2-Hz activity and delta-band AC–PFC coupling is compatible with repetition-related predictive or memory-related processing, while also potentially reflecting sensory tracking, temporal alignment to the repeated acoustic stream, or stimulus-rate-dependent modulation of the repeated acoustic sequence. The 400-ms ISI control condition, in which the stimulation rate increased to 2.5 Hz while the AC–PFC GC peak remained near ~2 Hz, argues against a purely one-to-one stimulus-locking account. However, temporal tracking and other sequence-related low-frequency mechanisms cannot be fully excluded. Accordingly, the present data do not by themselves establish the ~2-Hz response as a signature of temporal prediction.

Task engagement strongly modulated the strength and direction of functional coupling between AC and PFC. During active performance, GC-defined PFC → AC interactions were stronger than during passive listening (S10 Fig), mirroring the higher prevalence of repetition enhancement in PFC under behaviorally relevant conditions. This pattern is consistent with the possibility that prefrontal feedback contributes to auditory predictions when sustained attention to regularities is required. However, this interpretation should be treated cautiously, because field-potential connectivity metrics can also be shaped by common input, behavior-dependent changes in local oscillatory power, or activity arriving at AC and PFC with a temporal lag. Recent work on interareal coherence has shown that long-range field-potential coupling can arise from anatomical connectivity and oscillatory power, without necessarily implying direct oscillatory entrainment or direct causal communication [88]. In contrast, passive listening reduced PFC → AC drive, shifting the balance toward bottom-up AC-driven interactions. Such flexibility accords with predictive-coding frameworks in which precision weighting—shaped by behavioral relevance—dynamically regulates the balance between top-down predictions and bottom-up sensory evidence [71,89].

The persistence of delta-band coupling across repetition rates (S5 Fig) further underscores its role in maintaining temporal predictions. Unlike higher-frequency interactions linked to local processing or prediction-error signaling [90], delta oscillations appear to provide a scaffold for cross-cortical predictive maintenance. This interpretation aligns with evidence that delta-phase coding in AC sharpens temporal expectancy [77,91], while PFC leverages these rhythms to sustain working-memory representations [92]. Together, AC–PFC delta synchrony during repetition emerges as a dynamic *prediction loop* that stabilizes auditory regularities and optimizes processing for anticipated inputs.

### Deviance-related hierarchy and AC–PFC communication

Our findings outline a hierarchical architecture for deviance-related processing in the auditory system, defined by dynamic interactions between AC and PFC. Deviant tones evoked significant responses in both regions, yet their temporal dynamics diverged: deviations from standard responses emerged ~30–100 ms earlier in AC than in PFC (Figs 4A–4C, S6A, and S6B). This temporal lead is consistent with hierarchical deviance processing in which lower-order auditory regions respond earlier than higher-order regions [58,93]. The absolute timing of these deviance-related responses varied across animals, particularly in Monkey C, indicating that the latency of ECoG-defined prediction-error signals may depend on individual differences in electrode coverage, auditory-field sampling, signal-to-noise ratio, or task-related neural dynamics. Thus, our conclusion concerns the relative temporal ordering between AC and PFC within animals, rather than a fixed latency value across individuals. Despite this relative temporal lead, the overall magnitude of deviance-related activity—indexed by peak *F*-values—did not differ significantly between AC and PFC (Fig 4D). These results suggest that while AC rapidly flags sensory deviations, PFC contributes to their amplification and integration, linking error detection to higher cognitive functions such as attention and decision-making [94].

To characterize frequency-specific directed interactions during deviant processing, we examined connectivity dynamics across frequencies. In the active condition, AC → PFC differential GC was most consistently expressed in the theta/alpha and conventional gamma bands, whereas PFC → AC effects were predominantly observed in the theta range (Figs 5A–5C and S9). This frequency-specific organization is consistent with prediction-error-related AC → PFC interactions, while not excluding contributions from shared sensory drive or indirect pathways. Shortly thereafter, enhanced PFC → AC coupling in the same frequency range was consistent with feedback-related modulation, although this GC-defined interaction should not be interpreted as direct evidence of causal top-down signaling [93]. The strengthening of prefrontal feedback following deviant detection may reflect rapid recalibration of predictive templates, consistent with evidence implicating frontal activity in expectation updating after unexpected auditory events [93].

Although the GC analyses revealed frequency-specific directed interactions between AC and PFC, these findings warrant cautious interpretation. Granger causality quantifies whether past activity in one signal improves prediction of future activity in another signal, but it does not establish direct anatomical causality. In ECoG recordings, GC can be influenced by common input, indirect polysynaptic pathways, signal mixing, differences in signal-to-noise ratio, local spectral magnitude or power differences, and temporally aligned evoked or induced responses. In addition, because some analyses summarized GC estimates across many electrode pairs or channel maps, the resulting GC estimates should be interpreted as array-level directed interactions rather than precise communication between specific neuronal populations. Thus, the present data leave open the possibility that part of the AC–PFC coupling reflects shared sensory drive or common input to both regions rather than direct cortico-cortical transmission. Our results therefore identify behaviorally modulated, frequency-specific directed ECoG-level interactions within an anatomically connected auditory–prefrontal network, rather than proving direct monosynaptic feedforward or feedback signaling.

A related limitation concerns the distinction between ventral and dorsal auditory streams in primates [95,96]. In primates, auditory processing is often described in terms of partially segregated ventral and dorsal pathways, with the ventral stream more strongly implicated in sound identity and auditory object processing, and the dorsal stream more strongly implicated in spatial, sensorimotor, and action-related auditory processing [95,97]. The present task used non-spatial frequency deviants and therefore probed auditory feature-based novelty detection rather than spatial or vocal-motor processing. However, our recordings were limited to the temporal auditory-region array and lateral PFC, without coverage of orbitofrontal cortex, frontal pole, parietal cortex, or motor-related frontal regions. Thus, although the observed AC–PFC interactions reveal behaviorally modulated auditory–prefrontal predictive dynamics, they cannot be assigned unambiguously to either the ventral or dorsal auditory stream [63,97]. Future studies with broader frontal and temporal coverage will be needed to determine how prediction and prediction-error signals are distributed across ventral and dorsal auditory-stream circuits.

We also observed conventional gamma-band (30–70 Hz) AC → PFC GC-defined interactions during deviant processing. However, these effects should not be interpreted as direct evidence for spiking-related gamma-band burst coupling or for a fast membrane-potential oscillation. Although condition-specific ERPs were subtracted from individual trials before GC analysis to reduce phase-locked evoked components, gamma-band GC could still be influenced by temporally sharp induced responses, common sensory drive, or shared inputs to AC and PFC. The present analyses were performed at the ECoG level and did not specifically test lagged co-occurrence of gamma-band amplitude bursts between AC and PFC. Future work combining laminar recordings, spike-field analyses, and event-based gamma coupling analyses will be required to determine whether the ECoG-level gamma interactions observed here reflect coordinated local spiking activity, temporally sharp population activation, or other forms of fast population-level activity.

### Behavioral engagement shapes deviance-related dynamics

During active task performance, both AC and PFC exhibited stronger deviant responses and broader spatial differentiation of deviance-related activity than during passive listening (Figs 6E–6G). Connectivity analyses revealed frequency-specific GC-defined interactions during deviant processing. Under active task engagement, AC → PFC differential-GC patterns were most consistently expressed in theta/alpha and conventional gamma bands, whereas PFC → AC patterns were prominently observed in the theta range (Fig 5C). Passive-listening analyses further showed that stable AC → PFC interactions, particularly in theta and alpha bands, remained detectable even in the absence of active behavioral engagement (S11 Fig). These results converge with conceptual models in which prediction-error signaling is weighted by attention and task demands [89], highlighting behavioral relevance as a key factor shaping both the magnitude of local deviance-related responses and the frequency-specific structure of GC-defined interactions across cortical networks (Fig 7).

Together, the data support a bidirectional model of auditory predictive processing: AC responses emerged earlier than PFC responses during deviant processing, and GC analyses revealed recurrent AC–PFC interactions across frequency bands. These findings are consistent with recurrent auditory–prefrontal coordination during deviant processing, but they do not establish direct feedforward transmission of prediction errors or direct feedback transmission of predictions.

## Materials and methods

### Ethics statement

All experimental procedures were conducted in accordance with the Guide for the Care and Use of Laboratory Animals, European Union Directives 86/609/EEC, 2003/65/EC, and 2010/63/EU, and the Regulations for the Administration of Affairs Concerning Experimental Animals of the People’s Republic of China (GB 14925-2010). The experimental protocols were approved by the Bioethics Committee of Zhejiang University (approval no. ZJU20200148). Throughout the study, researchers and animal care staff closely monitored the animals daily to ensure their health and well-being. To enhance their quality of life, toys containing food items that the animals enjoyed were routinely introduced into their 0.74 m^3^ home cage to promote exploratory behavior. Regular assessments of the animals’ physical and physiological well-being were conducted by veterinarians, registered veterinary technicians, and researchers.

### Experimental model and surgical procedures

We conducted experiments in two adult male rhesus monkeys (*Macaca mulatta*; subjects C and X), aged 7 years and weighing 5.5–7 kg. Each monkey was implanted with two 64-channel subdural electrode arrays (E64-2500-50-200, NeuroNexus Technologies, USA), one over the left temporal auditory cortical region (AC array) and the other over the sampled left lateral prefrontal cortical surface (PFC array). The surgical and ECoG recording procedures followed those described in our previous report [98].

Aseptic techniques were employed throughout surgery. Monkeys were premedicated with ketamine (50 mg/kg) and medetomidine (0.03 mg/kg), then intubated and maintained under anesthesia with 1%–2% isoflurane in oxygen during surgery. Artificial ventilation was used as needed, and body temperature was maintained at 37 °C using an electric heating mat. Vital signs (oxygen saturation, heart rate, and end-tidal CO_2_) were continuously monitored to adjust anesthesia depth as required. Each subject’s head was stabilized in a stereotaxic frame (Narishige, Japan). After local infiltration with lidocaine, the scalp and overlying muscles were retracted, and a titanium headpost (Gray Matter Research, USA) was secured to the skull with bone screws and resin. Craniotomy and durotomy were performed under a surgical microscope (Carl Zeiss, Germany). Following implantation, the dura mater, bone flap, and skin were sutured, and the exposed area was covered with resin. Postoperative care included ketoprofen administration for three days and antibiotic treatment for one week to prevent infection and ensure recovery.

### ECoG recording

The ECoG electrodes had a diameter of 0.2 mm and gold recording sites embedded in a 2 × 2 cm polyimide substrate. Electrode impedances measured at 1 kHz ranged from 20 to 50 kΩ, with an inter-electrode spacing of 2.5 mm. Preoperative MRI scans were used to determine the target coordinates and craniotomy size for each subject.

The temporal array was positioned to broadly cover the auditory cortical region and adjacent temporal cortex around the lateral sulcus and superior temporal sulcus. Because not all temporal-array contacts could be assumed to belong to auditory cortex based on array placement alone, AC channels were defined using combined anatomical and functional criteria. Specifically, contacts located within the region between the lateral sulcus and superior temporal sulcus were included, and contacts immediately adjacent to these sulci were also included if they showed significant sound-evoked responses. Contacts clearly outside this anatomically plausible auditory cortical region and without significant auditory responsiveness were excluded from AC analyses. Thus, all analyses involving AC were performed using this anatomically and functionally defined AC channel set.

The prefrontal array was positioned over the lateral prefrontal cortex. Thus, throughout the manuscript, “PFC” refers to the sampled lateral PFC region rather than the entire prefrontal cortex. We did not further subdivide PFC contacts into dorsolateral PFC (DLPFC) and ventrolateral PFC (VLPFC), because the implanted array covered only part of the exposed lateral PFC surface, while the anatomical boundaries between dorsal and ventral prefrontal subdivisions depend partly on sulcal banks and folded cortical surfaces that cannot be resolved unambiguously from the surface ECoG layout. In addition, contacts near the principal and arcuate sulci may reflect signals from adjacent dorsal and ventral banks, making a reliable DLPFC/VLPFC assignment difficult. Therefore, PFC analyses were performed at the level of the sampled lateral PFC region. In contrast to the temporal array, all PFC contacts were retained for population-level statistical analyses and GC analyses.

A gold reference electrode was placed near the ECoG array within the subdural space and oriented toward the dura mater. Lead wires from both the ECoG and reference electrodes were connected to micro-connectors (ZIF-Clip 64, Tucker-Davis Technologies, USA), housed in a titanium chamber fixed to the skull with dental resin. Neural signals were amplified and band-pass filtered between 0.5 and 300 Hz using a differential amplifier (RZ5, Tucker-Davis Technologies).

### Sound stimulation

All recordings were performed in a sound-attenuated chamber. Monkeys were seated in a primate chair with the head fixed, the right ear oriented toward a free-field loudspeaker (LS50, KEF, UK), and one hand resting on a response button. Acoustic stimuli were digitally generated using a computer-controlled auditory workstation (RZ6, Tucker-Davis Technologies) at a 100 kHz sampling rate and delivered through the contralateral (right) speaker. Sound pressure levels were calibrated using a ¼-inch condenser microphone (Brüel & Kjær 4954, Denmark) connected to a PHOTON/RT analyzer (Brüel & Kjær, Denmark).

The stimuli consisted of complex tones composed of seven octave-spaced harmonic components, defined as:


$$\begin{array}{c} \text{y}(t)=\frac{\text{1}}{\text{7}}\sum_{i=\text{0}}^{\text{6}}\text{sin}\left[\text{2}\pi \left({\text{2}}^{i}f_{\text{0}}\right)t\right] \end{array}$$

where *t* represents time and *f*_0_ is the base frequency of the tone. The standard sound had a base frequency of 245 Hz, producing components at 245, 490, 980, 1,960, 3,920, 7,840, and 15,680 Hz. For deviant tones, the base frequency was altered relative to the standard according to the ratio *λ*, such that $f_{\text{dev}}=f_{\text{std}}\times \lambda$, with *λ* randomly selected from 1.02, 1.04, 1.06, or 1.08. Both standard and deviant tones had a duration of 100 ms and were presented at 60 dB SPL.

### Behavioral task

Monkeys were trained to perform a novelty-detection task based on an auditory oddball paradigm (Fig 1) [41,42]. Each trial began when the monkey pressed a button to initiate the sequence. Two seconds after the button press, a series of 8–11 complex tones were presented at a rate of 2 Hz, corresponding to a 500-ms inter-stimulus interval (ISI), defined as the time between sound onsets. A 5-s inter-trial interval was imposed, during which button presses were invalid.

Within each sequence, the initial tones were standards with identical frequencies, while the final tone served as the target. In deviant trials, the target tone differed in frequency from the preceding sounds, whereas in control trials it was identical. The monkey was required to press the button within 600 ms after deviant onset to receive a water reward. Responses earlier than 200 ms after target onset were considered unlikely to reflect genuine deviant detection, and the response window was set to reduce random button presses near the end of each sequence.

Deviant trials were defined by the frequency ratio (*λ*) between deviant and standard tones, with *λ* = 1.02, 1.04, 1.06, or 1.08. Control trials corresponded to *λ* = 1. Conditions were presented in random order and repeated 40–50 times per recording session. The number of standards preceding the target varied randomly between 7 and 10 to prevent anticipatory responses based on sequence length.

After each behavioral session, a passive session was conducted using the same auditory sequences, but a baffle was placed between the button and the monkey’s hands to block motor responses. Additionally, an extra behavioral session was recorded using a shorter ISI of 400 ms for both monkeys.

Behavioral performance was evaluated according to two criteria. First, Correct Hit (Deviant Identification): the monkey had to correctly identify the deviant sound with at least 80% accuracy (button-press ratio >0.8) for the largest frequency difference condition. Second, Correct Rejection (Absence of Deviant): in control trials, the monkey had to correctly withhold responses, achieving at least 90% accuracy (button-press ratio <0.1). Only recording sessions meeting both criteria were included in the analyses. In total, five behavioral and four passive sessions were included for each monkey.

### Data preprocessing

ECoG data were down-sampled to 500 Hz and band-pass filtered between 0.1 and 200 Hz, followed by a 50 Hz notch filter to remove line noise. The filtered signals were segmented from 3 s before to 7 s after the onset of the first sound in each trial. To reduce noise and artifact contamination, we performed independent component analysis (ICA) using the *runica* function from the *FieldTrip* toolbox [99]. Components were visually inspected and retained or rejected based on their spatial topography and variance.

After ICA, the data were re-referenced in three consecutive steps. First, we applied common average referencing (CAR) by subtracting the mean signal across all channels within each array. This step minimized inter-array differences related to electrode impedance and array-specific properties. Second, we applied orthogonal source derivation by subtracting the average signal from the four nearest neighboring electrodes (two horizontal and two vertical). This local re-referencing reduced residual common-reference and volume-conduction effects, thereby enhancing spatial specificity. Finally, the signals were normalized by their temporal standard deviation to ensure equal weighting across electrodes. The re-referenced data were retained for subsequent response, time-frequency, and Granger causality analyses.

For Granger causality (GC) analyses, we applied an additional preprocessing step to the re-referenced single-trial data. The average event-related potential (ERP) was subtracted from individual trials to reduce phase-locked evoked components associated with stimulus onsets and offsets. Importantly, ERP subtraction was performed separately within each analysis condition rather than across pooled conditions. For prediction GC analyses during the standard period, ERPs were computed and subtracted separately for trials with different numbers of standard sounds. For prediction-error GC analyses, ERPs were computed and subtracted independently for each frequency-ratio condition, including the control condition and each deviant condition (*λ* = 1, 1.02, 1.04, 1.06, and 1.08). GC was then calculated separately for each corresponding condition. This condition-specific ERP-subtraction procedure reduced the contribution of stereotyped evoked waveforms to GC estimates and allowed the analysis to focus more strongly on non-phase-locked activity and trial-by-trial functional interactions between cortical regions.

### Time–frequency analyses

Time–frequency analyses were performed using the MATLAB *cwt* function. Morlet wavelets with a time-bandwidth product of 5 were used as the analytic basis functions. The complex-valued CWT coefficients were converted to magnitude values by taking their absolute values.

For prediction analyses, the time–frequency responses (TFRs) during standard sound presentations were computed from trial-averaged time-domain responses. Because trials contained different numbers of standards before the final target sound, ERPs were first calculated separately for trials with different standard numbers and then combined using a weighted average according to the number of trials in each condition. To minimize edge artifacts introduced by the cone of influence (COI) in the wavelet transform, data segments were zero-padded to 10 s on both sides before transformation and subsequently trimmed to their original duration.

For GC analyses, TFRs were computed at the single-trial level from the ERP-subtracted data described above. In prediction GC analyses, only the standard sound period of each trial was included, from the onset of the first tone up to the onset of the deviant tone, or to one ISI after the last standard tone in control trials. For prediction-error GC analyses, TFRs were computed separately for each frequency-ratio condition using the 1 s period following the onset of the final deviant or standard sound.

### Local deviance-related time–frequency analyses

Local time–frequency responses aligned to the final-sound onset were computed from trial-averaged ECoG responses for each target/standard frequency ratio. For each deviant ratio (*λ* = 1.02, 1.04, 1.06, and 1.08), deviant-minus-standard ΔMagnitude was calculated by subtracting the CWT magnitude of the standard condition (*λ* = 1) from that of the deviant condition. For the example maps in S7A and S7B Fig, ΔMagnitude was shown for the same AC and PFC example channels used in Fig 4A and 4B. For band-specific quantification, ΔMagnitude was averaged within 0–350 ms after final-sound onset and within canonical frequency bands: δ, 0.6–4 Hz; θ, 4–8 Hz; α, 8–14 Hz; β, 14–30 Hz; γ, 30–70 Hz. Statistical significance was assessed across channels using one-sample right-tailed *t*-tests against zero for each frequency-band × deviant-ratio combination, and *p*-values were FDR-corrected across these comparisons within each area and monkey.

### Repetition-related suppression and enhancement analyses

Repetition-related suppression and enhancement were quantified by averaging spectral magnitude within a frequency band centered around the stimulus repetition rate (± 0.2 Hz relative to the repeat rate). This corresponded to 1.8–2.2 Hz for the 500-ms ISI and 2.3–2.7 Hz for the 400-ms ISI (Figs 2G and S3C).

To estimate temporal changes in repetition-related activity, the mean spectral magnitude at the repetition rate was fitted across the sequence of standard sounds, specifically from the 5th to the 9th complex tone. For analyses aligned by standard-stimulus presentation order, each order was calculated using all trials that contained that order. Thus, trials with 7, 8, 9, or 10 standards all contributed to early standard orders, whereas only trials containing later repetitions contributed to the corresponding later orders. Slopes from these linear fits were compared between cortical regions (AC versus PFC), ISI conditions (400 versus 500 ms), and behavioral contexts (behavioral versus passive).

For the channel-wise slope maps in Figs 2I and S3, the significance of the repetition-rate magnitude slope was assessed using a bootstrap procedure. Trials were first grouped according to the number of standards preceding the final target sound. Within each standard-number group, trials were resampled with replacement, and the corresponding trial-averaged response was recomputed. The resampled responses from different standard-number groups were then combined using a weighted average according to the number of trials in each group, and the repetition-rate magnitude slope was recalculated. This procedure was repeated 1,000 times for each channel, yielding a bootstrap distribution of slope estimates. A two-tailed bootstrap *p*-value was estimated as 2 × min[*P*(slope ≤ 0), *P*(slope ≥ 0)], and channels with *p* < 0.05 were considered significant. Channels with positive 2-Hz magnitude slope values were classified as showing repetition enhancement, whereas channels with negative slope values were classified as showing repetition suppression. The proportions of enhancement and suppression channels were then compared between behavioral and passive conditions for each cortical area.

### Granger causality (GC) analyses

Granger causality quantifies whether past values of one time series improve the prediction of future values of another time series. We used a nonparametric GC approach in which the transfer matrix and residual covariance matrix were derived by factorizing the cross-spectral density (CSD) matrix. The CSD was computed from wavelet-transformed single-trial spectra for all electrode pairs between the temporal array and the PFC array. GC estimates were therefore initially obtained for all temporal-array and PFC contacts. For subsequent population-level statistical analyses and reported AC topographic summaries, however, AC contacts were restricted to the anatomically and functionally defined AC channel set described above, whereas all PFC-array contacts were retained.

Before GC estimation, ERP subtraction was performed separately within each analysis condition, as described above. For prediction GC analysis, all trials without button presses during the standard-sound period were included, spanning from the onset of the first sound to the onset of the deviant sound, or to one ISI after the last standard tone in control trials. Trials containing different numbers of standard tones (7–10) were analyzed separately, and condition-specific ERPs were subtracted before GC computation. For prediction-error GC analyses, data were extracted from passive sessions and from successful behavioral trials, spanning from the onset of the final sound to 1 s post-stimulus. Prediction-error GC was computed separately for each frequency-ratio condition. For each ratio condition (*λ* = 1, 1.02, 1.04, 1.06, and 1.08), the corresponding condition-specific ERP was first subtracted from individual trials, and GC was then estimated independently for that condition. Thus, differential GC between deviant and standard conditions was calculated from GC estimates obtained after ratio-specific ERP subtraction, rather than from pooled ERP-subtracted data.

Nonparametric GC values were computed bidirectionally (AC → PFC and PFC → AC) for all electrode pairs, across 72 frequency bins (0.6–70 Hz) and temporal bins (2 ms step size). Baseline GC values, obtained from the pre-stimulus period (−3 to −2.5 s relative to the first sound onset), were subtracted from subsequent results. Frequencies were grouped into five canonical bands: δ (0.6–4 Hz), θ (4–8 Hz), α (8–14 Hz), β (14–30 Hz), and γ (30–70 Hz), and GC values were averaged within each band.

To estimate average GC-defined directed interactions across cortical regions, GC maps were first computed for each electrode by averaging its GC values with all electrodes in the opposite array. For AC-centered summaries, only electrodes belonging to the anatomically and functionally defined AC channel set were entered into subsequent population-level statistics and topographic summaries. For PFC-centered summaries, all PFC-array contacts were retained. This procedure was performed separately for both directions (AC → PFC and PFC → AC), frequency-ratio conditions (*λ* = 1, 1.02, 1.04, 1.06, and 1.08), frequency bands, and overlapping time windows (100-ms bins with 50-ms steps).

For prediction GC analyses, temporal dynamics of GC-defined interactions were assessed by computing Spearman’s rank correlations of GC values across time bins corresponding to sound orders 5–6 during the standard period. For the 500-ms ISI condition, this corresponded to the 2–3 s window after the onset of the first sound. Topographic maps of Spearman’s *ρ*, averaged across conditions with 7–10 standard tones, were generated for each frequency band and direction. Electrodes showing significant correlations (*p* < 0.05, FDR-corrected across electrodes) were marked with an ‘o’.

For prediction-error GC analysis, differential GC maps were constructed by subtracting the GC map for the standard condition (*λ* = 1) from that for each deviant condition. To evaluate whether deviance-related GC topographies were stable across deviant strengths, we computed cross-condition spatial correlation matrices. The *λ* = 1.02 condition was excluded because of the limited number of successful trials; therefore, this analysis used deviant conditions with *λ* = 1.04, 1.06, and 1.08. GC values were averaged within each frequency band and within sliding post-stimulus time windows. For each monkey, behavioral context, direction (AC → PFC or PFC → AC), cortical map (AC or PFC), frequency band, and pair of deviant conditions, Spearman’s rank correlations were computed between differential GC topographies across channels for all pairs of time windows. This produced a time-by-time correlation matrix in which each element quantified the spatial similarity between deviance-related GC maps from two deviant conditions at two time windows. For visualization, correlation coefficients were averaged across the three deviant-condition pairs (1.04 versus 1.06, 1.04 versus 1.08, and 1.06 versus 1.08). A time-window pair was considered consistently correlated in one monkey when all three deviant-condition pairs showed positive correlations at *p* < 0.05. Time-window pairs meeting this criterion in both monkeys were marked separately. These exploratory correlation tests were not corrected for multiple comparisons.

### Statistical analysis

Spearman’s rank correlation coefficients (*ρ*) were computed in two contexts. First, for prediction GC analyses, Spearman correlations were used to quantify temporal changes in GC-defined interactions during the standard-repetition period (Figs 3C, S4, S5C, and S10B). For these analyses, right-tailed *p*-values were used, and significant electrodes were identified after FDR correction across electrodes. Second, for prediction-error GC analyses, Spearman correlations were used to assess the topographic stability of differential GC maps across deviant conditions (Figs 5C, S9, and S11). For these time-by-time correlation matrices, a time-window pair was considered significant when the differential GC topographies were positively correlated across deviant-condition pairs at *p* < 0.05. These exploratory correlation tests were not corrected for multiple comparisons.

Two-way ANOVAs were used to test whether repetition-rate magnitude slopes differed across cortical regions and monkeys (Figs 2H and S3D). For these analyses, cortical area and monkey identity were treated as between-channel factors. To compare repetition-rate magnitude slopes between behavioral and passive conditions (Fig 6C), we used repeated-measures two-way ANOVA separately for AC and PFC, with behavioral context (behavioral versus passive) as a within-channel factor and monkey identity as a between-channel factor. Effect sizes for ANOVAs were expressed as partial eta squared (*η*^2^):


$$\begin{array}{c} \eta_{k}^{\text{2}}=\frac{SS_{k}}{SS_{k}+SS_{\text{error}}} \end{array}$$

where *k* denotes the tested factor, *SS*_*k*_ is the sum of squares for that factor, and *SS*_error_ is the residual sum of squares. Values of 0.01, 0.06, and 0.14 were interpreted as small, medium, and large effects, respectively.

For the categorical analysis of repetition enhancement and repetition suppression (Fig 6D), channels were classified according to the sign of the repetition-rate magnitude slope. Positive slopes were classified as repetition enhancement, whereas negative slopes were classified as repetition suppression. Because the same channels were compared between behavioral and passive conditions, changes in enhancement/suppression classification were assessed using exact McNemar tests separately for AC and PFC.

Two-tailed Mann–Whitney *U* tests were used to compare the first significant time and maximum *F*-values between AC and PFC (Fig 4C and 4D). For these comparisons, only channels showing significant response differences across target/standard frequency ratios within 0–350 ms after final-sound onset were included. The first significant time was defined as the earliest significant time bin within this window, and the maximum *F*-value was calculated within the same 0–350 ms window for the included channels. Effect size was expressed as rank-biserial correlation:


$$\begin{array}{c} =\frac{\left|Z\right|}{\sqrt{n_{\text{1}}+n_{\text{2}}}} \end{array}$$

where *Z* is the standardized Mann–Whitney *U* statistic and *n*_1_, *n*_2_ are sample sizes of the two groups. Values of 0.1, 0.3, and 0.5 indicated small, medium, and large effects, respectively.

Two-tailed paired *t*-tests were used to compare maximum *F*-values between behavioral and passive conditions across matched channels (Fig 6G).

To evaluate differences in neural responses across target/standard frequency-ratio conditions (Figs 4A, 4B, and 6E), we applied non-parametric cluster-based permutation tests using the *FieldTrip* toolbox. Event-related responses were first averaged across trials for each of the five frequency-ratio conditions. At each time point and electrode, an *F*-test across conditions produced an *F*-value per sample. Channels adjacent in orthogonal directions on the ECoG grid were defined as neighbors. Samples exceeding *p* < 0.05 before correction were grouped into spatiotemporal clusters, and *F*-values within each cluster were summed to yield a cluster-level statistic. Condition labels were randomly shuffled 1,000 times to generate a null distribution of maximum cluster-level statistics. Observed clusters were considered significant if their cluster-level statistics exceeded the 95th percentile of the null distribution, corresponding to a corrected threshold of *p* < 0.05.

For local deviance-related time-frequency analyses (S7 Fig), deviant-minus-standard ΔMagnitude was computed by subtracting the CWT magnitude of the standard condition (target/standard ratio = 1) from that of each deviant condition (target/standard ratios = 1.02, 1.04, 1.06, and 1.08). For band-specific quantification, ΔMagnitude was averaged within 0–350 ms after final-sound onset and within canonical frequency bands: δ, 0.6–4 Hz; θ, 4–8 Hz; α, 8–14 Hz; β, 14–30 Hz; and γ, 30–70 Hz. For each area, monkey, frequency band, and deviant ratio, one-sample right-tailed *t*-tests were used to test whether ΔMagnitude was greater than zero across channels. *p*-values were FDR-corrected across frequency-band × deviant-ratio comparisons within each area and monkey.

To assess whether GC values were larger for deviant than standard conditions, one-tailed permutation tests were performed for topographic GC analyses (Figs 5B and S8). For each electrode, direction, frequency band, and time window, GC values from deviant and standard trials were randomly shuffled across 1,000 permutations to generate a null distribution of differential GC values. The p-value was defined as the proportion of permuted values exceeding the observed deviant-minus-standard difference. *p*-values were FDR-corrected across electrodes within each map, and electrodes with corrected *p* < 0.05 were considered significant.

All data preprocessing, time–frequency transformations, and statistical analyses were performed using custom MATLAB scripts (R2021a, MathWorks) in combination with the *FieldTrip* toolbox, as well as supplementary routines written in Python (v3.9). Figures were generated in MATLAB and refined in Adobe Illustrator for publication. Unless otherwise stated, statistical significance was established at *p* < 0.05.

## Supporting information

S1 Fig ECoG array implantation sites. (**A**) ECoG array implantation in monkey X. For each region, the left image shows the cortical surface before implantation, the middle image shows the array after implantation, and the right schematic shows electrode positions relative to visible sulcal landmarks. Top, AC; bottom, PFC. (**B**) Same as (A), for monkey C. The central sulcus was not clearly visible in the surgical photographs and was therefore not labeled. Scale bars, 2.5 mm. AC, auditory cortex; PFC, lateral prefrontal cortex; asl, lower limb of the arcuate sulcus; asu, upper limb of the arcuate sulcus; ps, principal sulcus; ls, lateral sulcus; sts, superior temporal sulcus; C, caudal; V, ventral.
(TIF)

S2 Fig Sound-evoked responses used to define AC channels. (**A**) Event-related potentials aligned to the onset of the first standard sound in the temporal array of monkey X. Traces show responses within 0–300 ms after sound onset for each channel. Purple box indicates channels included in AC analyses. The red and blue boxes indicate the example AC channels showing repetition enhancement and repetition suppression in Fig 2, respectively. Right, topographic map of sound-evoked response magnitude, quantified as ΔRMS = RMS(0 to 300 ms) − RMS(−200 to 0 ms). Open circles indicate channels with significant positive ΔRMS (one-tailed one-sample *t* test, *p* < 0.05, FDR-corrected). (**B**) Same as (A), for monkey C.
(TIF)

S3 Fig Repetition-related suppression and enhancement under the 400-ms ISI condition. (**A, B**) Example AC (#52) and PFC (#30) channels from monkey X showing repetition-related enhancement under the 400-ms ISI condition. Top, trial-averaged normalized response; bottom, corresponding time–frequency magnitude. Vertical dashed lines indicate standard-tone onsets, and the horizontal dashed line marks 2.5 Hz, corresponding to the 400-ms onset-to-onset interval. (**C**) Time course of magnitude in the 2.3–2.7 Hz band for the example channels. Slanted dashed lines show linear fits from 1.6 to 3.6 s, corresponding to response windows for standards 5–9; the endpoint at 3.6 s marks the onset boundary of the 10th standard. (**D**) Dot plots of 2.3–2.7 Hz magnitude slopes across included channels in AC and PFC of both monkeys. Each dot represents one channel; horizontal lines indicate the median. (**E**) Channel-wise comparison of magnitude slopes between the 500- and 400-ms ISI conditions. (**F**) Topographic maps of magnitude slopes under the 400- and 500-ms ISI conditions for AC and PFC in both monkeys.
(TIF)

S4 Fig Topographic maps of GC-defined interactions during standard repetition in monkey X. Same analysis as Fig 3C, shown for monkey X. Topographic maps show Spearman correlations of GC values across consecutive time bins during the standard-repetition period, separately for AC → PFC and PFC → AC directions and for the 2-Hz bin and canonical frequency bands. Circled channels indicate significant correlations (*p* < 0.05, FDR-corrected across channels; see Materials and methods).
(TIF)

S5 Fig GC-defined interactions between AC and PFC during standard repetition under the 400-ms ISI condition. (**A**) Time–frequency maps of bidirectional GC between AC and PFC in monkey X under the 400-ms ISI condition, averaged across AC–PFC channel pairs. GC was aligned to the onset of the first standard sound; for visualization, only the later repetition period from 0.8 to 2.4 s is shown, beginning at the onset of the third standard sound. Side traces show GC averaged over the displayed time window. (**B**) Time courses of GC in the 2-Hz bin and canonical frequency bands for both directions. Horizontal yellow bars indicate the time window used for temporal-correlation analysis. (**C**) Topographic maps of temporal correlations in GC for each frequency band and direction. Circled channels indicate significant correlations (Spearman’s *ρ*, *p* < 0.05, FDR-corrected across channels; see Materials and methods).
(TIF)

S6 Fig Temporal profiles of deviance-related response differences across channels. (**A**) Channel-by-time maps of *F*-values for AC and PFC in monkey X, aligned to the onset of the final sound. *F*-values were computed across the five target/standard frequency ratios at each time point. Channels are sorted by the first significant time of deviance-related response differences. (**B**) Same as (A), for monkey C.
(TIF)

S7 Fig Local time-frequency responses during deviance processing. (**A**) Deviant-minus-standard local time-frequency magnitude maps for the same example AC channel (#38, monkey X) shown in Fig 4A, aligned to final-target onset and shown separately for target/standard frequency ratios of 1.02, 1.04, 1.06, and 1.08. ΔMagnitude was computed by subtracting the standard condition (ratio = 1) from each deviant condition. (**B**) Same as (A), for an example PFC channel (#47, monkey X). (**C**) Band-specific quantification of deviant-minus-standard time-frequency magnitude in AC for monkey X and monkey C. For each deviant ratio, ΔMagnitude was averaged within 0–350 ms after final-target onset and within canonical frequency bands. (**D**) Same as (C), for PFC. Asterisks indicate significant positive deviant-minus-standard magnitude across channels after FDR correction across frequency-band × deviant-ratio comparisons (*p < 0.05, **p < 0.01, ***p < 0.001).
(TIF)

S8 Fig Frequency-band topographies of deviance-related differential GC. (**A**) Differential GC topographic maps for monkey X, averaged within 0–100 ms after final-sound onset. Differential GC was defined as deviant GC minus standard GC and was computed separately for each frequency band and direction. Maps are shown for δ, θ, α, β, and γ bands in both AC → PFC and PFC → AC directions. (**B**) Same as (A), for monkey C.
(TIF)

S9 Fig Cross-condition stability of deviance-related differential GC topographies in monkey C. Same analysis as Fig 5C, shown for monkey C. Colors indicate the mean Spearman’s *ρ* across deviant-condition pairs. Dots and stars mark significant correlations in monkey C and in both monkeys, respectively (*p* < 0.05, uncorrected; see Materials and methods).
(TIF)

S10 Fig GC-defined interactions between AC and PFC during passive standard repetition. (**A**) Time–frequency maps of bidirectional GC between AC and PFC during passive presentation of repeated standard sounds, averaged across AC–PFC channel pairs. GC was aligned to the onset of the first standard sound; for visualization, only the 1–3 s repetition period is shown. (**B**) Topographic maps of temporal GC correlations in the 2-Hz bin and δ band under the passive condition. Maps are shown for AC → PFC and PFC → AC directions in both monkeys. The color scale indicates Spearman’s *ρ*. No channels showed significant temporal GC correlations.
(TIF)

S11 Fig Cross-condition stability of deviance-related differential GC topographies under passive listening. (**A**) Time-by-time correlation matrices showing the spatial stability of differential GC topographies across deviant conditions in monkey X during passive listening. Differential GC was defined as deviant minus standard GC. Colors indicate the mean Spearman’s *ρ* across deviant-condition pairs, computed separately for each frequency band, direction, and cortical map. Dots indicate significant correlations in monkey X, and stars indicate significant correlations in both monkeys (*p* < 0.05, uncorrected; see Materials and methods). (**B**) Same as (A), for monkey C.
(TIF)

## Abbreviations

AC: auditory cortex
CAR: common average referencing
COI: cone of influence
CSD: cross-spectral density
CWT: continuous wavelet transform
DLPFC: dorsolateral PFC
ECoG: electrocorticography
EEG: electroencephalogram
ERP: event-related potential
FDR: false discovery rate
GC: Granger causality
IC: inferior colliculus
ICA: independent component analysis
ISI: inter-stimulus interval
MEG: magnetoencephalogram
MMN: mismatch negativity
PFC: prefrontal cortex
RMS: root mean square
SSA: stimulus-specific adaptation
TFRs: time–frequency responses
VLPFC: ventrolateral PFC
