# Supplementary material for: Behavioral engagement and stimulus regularities coordinate auditory-prefrontal network activity
Source: PLoS Biol. 2026 Aug 26;24(8):e3003966. doi: 10.1371/journal.pbio.3003966 (PMC13537682; doi:10.1371/journal.pbio.3003966)
Supplement: Supplementary file 1 [file pbio.3003966.s014.docx]

Reviewer #1 [Julio Hechavarria]

This is my assessment of the article entitled: Behaviorally gated hierarchical predictive coding in macaque auditory-prefrontal circuits submitted to Plos Biology by Xu et al. The article deals with the question of how unpredictable and predictable auditory events are represented in two cortical areas: the PFC and AC. These two areas likely are fundamental parts of the auditory network and as such they need to be studied simultaneously to understand how they bind information together.
In the article the authors present simultaneous ECOG recordings form frontal and AC areas in two monkeys that were either engaged in a detection task or passively listening to mixtures of predictable and unpredictable events. The results clearly show the existence of interareal coupling in the delta and low theta frequency bands of the field potentials recorded, as well as distinct (likely complementary) representations of deviant and standard sours in the PFC-AC network. I enjoyed the paper very much and I think it is a great match for PLOS Biology as it sheds light into the neural substrates that allow the brain to process complex sound streams. While the paper is very good, I do see several points where clarification is needed, particularly regarding the interpretation of Granger Causality results and how they might be constrained by the underlying circuit anatomy. My specific comments are listed below.

**Our Response**

We thank Dr. Hechavarria for this very positive and thoughtful assessment of our study. We are grateful for your recognition that simultaneous ECoG recordings from auditory and prefrontal cortical regions in behaving and passive-listening macaques provide a valuable opportunity to examine how predictable and unpredictable auditory events are represented across the AC–PFC network. We also appreciate your positive evaluation of the interareal delta/low-theta coupling results and of the complementary representations of standard and deviant sounds across these regions.

We fully agree with your main concern that the interpretation of Granger causality results should be carefully constrained by the underlying circuit anatomy and by the known limitations of functional connectivity analyses. Therefore, we have clarified three points throughout the Results and Discussion in the revised manuscript.

First, we added anatomical context for AC–PFC interactions in macaques, including the established reciprocal connections between auditory temporal cortex and prefrontal regions, while also acknowledging that the present ECoG data do not isolate direct monosynaptic interactions from indirect pathways or common inputs.

Second, we tempered the language used to interpret Granger causality. Rather than treating GC as evidence of direct causal communication, we described it as a directional, statistical measure of frequency-specific interareal predictability and functional interaction. We explicitly stated that GC can be influenced by common input, signal mixing, differences in signal-to-noise ratio, and evoked response structure, and therefore should be interpreted as evidence for directed network dynamics rather than as proof of direct anatomical transmission.

Third, we revised the Discussion to consider alternative interpretations of the observed delta/theta and gamma-band interactions, including the possibility that some connectivity patterns reflect shared sensory drive, common input to both AC and PFC, or temporally aligned evoked responses, rather than exclusively feedforward prediction-error or feedback prediction signals.

These changes will make the manuscript more balanced and will better align the interpretation of the GC findings with the strength and limits of the data. We are grateful for this comment because it helps us sharpen the anatomical and methodological framing of the study while preserving the central finding that behavioral engagement strongly modulates large-scale AC–PFC network dynamics during auditory novelty detection.

Comments:

Colors in Fig. 2 G are difficult to distinguish. Consider a different color scale?
**Our Response**

We thank you for this helpful suggestion. We agree that the original color scheme in Fig. 2G was not sufficiently clear, especially because the three traces were displayed with colors that were too similar to one another. To improve readability, we have revised Fig. 2G using a clearer, color-blind–friendly color scheme with stronger contrast between the three example channels. We have also increased the line thickness and improved legend to facilitate visual discrimination of the traces.

These revisions improve figure clarity without affecting the analysis or interpretation of the results.

Line 280-> the word entrainment is used here to indicate evoked responses that track the acoustic stream but it could imply that there are temporal predictions occurring which is not clearly tested here.

**Our Response**

We thank you for this important clarification. We agree that the term “entrainment” may imply a stronger interpretation than is directly supported by the present analysis, because it could suggest that the observed 2-Hz ECoG activity reflects temporal prediction rather than low-frequency modulation associated with the repeated acoustic sequence. In the revised manuscript, we have therefore replaced “entrainment” with more descriptive and neutral wording, such as “repetition-related low-frequency modulation” and “2-Hz repetition-related ECoG activity.”

We also softened the interpretation of the 2-Hz response throughout this section. Rather than presenting this activity as direct evidence for temporal prediction, we now describe it as a repetition-related low-frequency ECoG signal that is consistent with the buildup of predictive or memory-related representations, while acknowledging that it may also reflect neural tracking of the repeating acoustic sequence.

In addition, because this issue is closely related to the broader interpretation of delta-band AC–PFC coupling, we added an explicit cautionary statement in the Discussion. This new text clarifies that the present data do not by themselves prove that the ~2-Hz response reflects temporal prediction rather than sequence-related low-frequency modulation.

These revisions make the interpretation more cautious and better aligned with the data.

**Revised text in the manuscript:**

“The average slope of 2-Hz-band magnitude was significantly steeper in PFC than in AC (F(1,197) = 16.66, p < 0.001, η^2^ = 0.08; two-way ANOVA), indicating stronger repetition-related low-frequency modulation in PFC. Together, these results show that repetition enhancement is particularly prominent in prefrontal regions, where it is reflected in the progressive buildup of 2-Hz repetition-related ECoG activity.” (P.10, Lines 230–234)

“Notably, when the repetition rate was increased from 2 Hz to 2.5 Hz using a 400-ms ISI, GC peaks remained near ~2 Hz rather than shifting to the 2.5-Hz stimulation rate (Supplementary Fig. 5A). This observation suggests that low-frequency AC–PFC coupling reflects more than direct tracking of the external stimulus rhythm, although sensory tracking, temporal alignment to the repeated acoustic stream, and other sequence-related low-frequency processes may contribute to the observed effects.” (P.12, Lines 269–274)

“The buildup of ~2-Hz activity and delta-band AC–PFC coupling is compatible with repetition-related predictive or memory-related processing, while also potentially reflecting sensory tracking, temporal alignment to the repeated acoustic stream, or stimulus-rate-dependent modulation of the repeated acoustic sequence. The 400-ms ISI control condition, in which the stimulation rate increased to 2.5 Hz while the AC–PFC GC peak remained near ~2 Hz, argues against a purely one-to-one stimulus-locking account. However, temporal tracking and other sequence-related low-frequency mechanisms cannot be fully excluded. Accordingly, the present data do not by themselves establish the ~2-Hz response as a signature of temporal prediction.” (P.21, Lines 588–596)

GC calculations. I commend the authors on these results. The delta/theta coupling between AC and PFC seems robust and is beautifully illustrated here. It seems this coupling is evolutionarily preserved (i.e., see papers on bats). I would have liked to have a bit more context here in the sense of the anatomy of the circuit in monkeys. Are there direct bidirectional projections between the PFC and AC? In other species, the PFC receives a fast input the by-passes the canonical auditory pathway and produces latencies that are shorter than those observed in the AC. Can we rule out that the connectivity reported here is not just a consequence of both areas receiving common input as opposed to the interaction between them? Beyond the low frequency LFPs, did you observe any type of interactions in the high gamma range that is usually related to spiking? i.e. gamma bursts co-occurring in the two areas with a certain lag?

**Our Response**

We thank you for this important and constructive comment. We are grateful for your positive assessment of the delta/theta AC–PFC coupling results, and we fully agree that these findings require clearer anatomical and methodological framing.

First, we have expanded the anatomical background for auditory–prefrontal interactions in macaques. In the revised manuscript, we now state that reciprocal anatomical connections between auditory temporal cortex and prefrontal regions have long been established, and that higher-order auditory cortical fields, including belt and parabelt regions, are anatomically connected with lateral prefrontal regions. Anatomical tracing studies in macaques have shown that anterior auditory belt cortex projects to frontal pole, rostral principal sulcus, and ventral prefrontal regions, whereas caudal auditory belt cortex preferentially targets more caudal prefrontal and frontal-eye-field regions. Parabelt auditory cortex also shows connections with dorsal, orbital, and polar prefrontal regions. These anatomical findings provide a plausible structural substrate for the AC–PFC interactions observed in our ECoG recordings.

Second, we agree that anatomical connectivity and Granger causality do not allow us to conclude that the observed coupling reflects direct monosynaptic communication between AC and PFC. Both regions may receive common or indirect sensory-related inputs, including pathways that bypass the canonical lemniscal auditory hierarchy. We have therefore revised the manuscript to explicitly acknowledge that the present ECoG data cannot exclude common input, shared sensory drive, or indirect polysynaptic pathways as contributors to the observed AC–PFC coupling.

Third, we have softened the interpretation of Granger causality throughout the manuscript. Rather than treating GC as evidence for direct causal transmission, we now describe it more cautiously as a directional measure of statistical dependence or functional interaction between ECoG signals. We also added a dedicated cautionary paragraph stating that GC can be influenced by common input, indirect pathways, signal mixing, differences in signal-to-noise ratio, and temporally aligned evoked responses.

Fourth, regarding high-gamma interactions, we did observe gamma-band GC effects during deviant processing, especially in the AC→PFC direction (Fig. 5C; Supplementary Figs. 8–9). However, we agree that these effects should not be interpreted as direct evidence for spike-related high-gamma burst coupling. The present analyses were performed at the ECoG level and did not include a dedicated high-gamma burst analysis, such as lagged co-occurrence of high-gamma amplitude events between AC and PFC. We have therefore clarified that the gamma-band GC effects should be interpreted as ECoG-level fast population interactions, not as direct evidence for spike-related high-gamma bursts.

Finally, following your suggestion, we have broadened the cross-species context of the Discussion. We now cite studies in bats showing robust fronto-auditory coupling during spontaneous activity and auditory processing, as well as behavior-dependent reversals of directed functional interactions between frontal and auditory cortices during vocal behavior. These findings provide valuable comparative evidence that frontal–auditory coupling may represent an evolutionarily conserved motif linking auditory processing with behavioral context.

Together, these revisions make our interpretation of the GC results more anatomically grounded and methodologically cautious, while preserving the central conclusion that auditory and prefrontal cortices show behaviorally modulated, frequency-specific directed interactions during auditory novelty detection.

**Revised text in the manuscript**

“Reciprocal anatomical connections between these regions have long been established. In macaques, higher-order auditory cortical fields, including belt and parabelt regions, are anatomically connected to lateral prefrontal regions, providing a structural substrate for auditory–prefrontal interactions. Nevertheless, such anatomical connectivity alone does not demonstrate that field-potential coupling reflects direct monosynaptic communication, as both regions may also receive common or indirect sensory-related inputs.” (P.3–4, Lines 96–101)

“Topographic GC maps corroborated these effects (Fig. 5B): bidirectional GC-defined interactions were minimal for standards but rose sharply for deviants, particularly in the AC→PFC direction, with subtraction maps showing larger deviance-related increases in this direction. Across frequency bands, deviant stimuli elicited significant GC-defined directed interactions from delta to gamma ranges in both directions and across both monkeys (Supplementary Fig. 8). Temporal correlation matrices of differential GC maps across frequency-ratio pairs (1.04 vs. 1.06, 1.04 vs. 1.08, 1.06 vs. 1.08) further showed consistent AC→PFC patterns in the theta/alpha (4–14 Hz) and conventional gamma (30–70 Hz) bands, while PFC→AC patterns predominated in the theta (4–8 Hz) range (Fig. 5C). These effects were reproduced in the second monkey (Supplementary Fig. 9), consistent with frequency-specific, bidirectional ECoG-level coordination between auditory and prefrontal cortices during deviant detection.” (P.15, Lines 352–363)

“Although the GC analyses revealed frequency-specific directed interactions between AC and PFC, these findings warrant cautious interpretation. Granger causality quantifies whether past activity in one signal improves prediction of future activity in another signal, but it does not establish direct anatomical causality. In ECoG recordings, GC can be influenced by common input, indirect polysynaptic pathways, signal mixing, differences in signal-to-noise ratio, local spectral magnitude or power differences, and temporally aligned evoked or induced responses. In addition, because some analyses summarized GC estimates across many electrode pairs or channel maps, the resulting GC estimates should be interpreted as array-level directed interactions rather than precise communication between specific neuronal populations. Thus, the present data leave open the possibility that part of the AC–PFC coupling reflects shared sensory drive or common input to both regions rather than direct cortico-cortical transmission. Our results therefore identify behaviorally modulated, frequency-specific directed ECoG-level interactions within an anatomically connected auditory–prefrontal network, rather than proving direct monosynaptic feedforward or feedback signaling.” (P.22–23, Lines 655–669)

“We also observed conventional gamma-band (30–70 Hz) AC→PFC GC-defined interactions during deviant processing. However, these effects should not be interpreted as direct evidence for spiking-related gamma-band burst coupling or for a fast membrane-potential oscillation. Although condition-specific ERPs were subtracted from individual trials before GC analysis to reduce phase-locked evoked components, gamma-band GC could still be influenced by temporally sharp induced responses, common sensory drive, or shared inputs to AC and PFC. The present analyses were performed at the ECoG level and did not specifically test lagged co-occurrence of gamma-band amplitude bursts between AC and PFC. Future work combining laminar recordings, spike-field analyses, and event-based gamma coupling analyses will be required to determine whether the ECoG-level gamma interactions observed here reflect coordinated local spiking activity, temporally sharp population activation, or other forms of fast population-level activity.” (P.23, Lines 686–697)

“For Granger causality (GC) analyses, we applied an additional preprocessing step to the re-referenced single-trial data. The average event-related potential (ERP) was subtracted from individual trials to reduce phase-locked evoked components associated with stimulus onsets and offsets. Importantly, ERP subtraction was performed separately within each analysis condition rather than across pooled conditions.” (P.27–28, Lines 864–868. Methods)

“More broadly, this interpretation is also consistent with cross-species evidence that frontal–auditory coupling is not unique to macaques or humans. In bats, simultaneous recordings from frontal auditory field and auditory cortex have revealed robust fronto-temporal coupling during spontaneous activity and auditory processing, as well as behavior-dependent reversals of directed functional interactions between frontal and auditory cortices during vocal behavior. In rodents, orbitofrontal cortex modulates auditory cortical sensitivity and sound perception, supporting the idea that frontal regions can shape auditory cortical processing according to behavioral context. In marmosets, frontal–auditory cortical interactions during vocal production have been linked to sensory prediction and feedback-dependent control. Together, these studies suggest that frontal–auditory coordination may represent an evolutionarily conserved circuit motif for linking auditory processing with behavioral context.” (P.21, Lines 575–586)

L332-334: yes but in Monkey C already occurred quite late even for the AC which could indicate high variability in the results.

**Our Response**

We thank you for this important point. We agree that the absolute latency of deviance-related responses was more variable across animals than our original wording suggested. Although deviant–standard differences emerged significantly earlier in AC than in PFC in both monkeys, the absolute first significant times were generally later in Monkey C, including within AC.

In the revised manuscript, we have therefore removed wording implying a fixed absolute latency of deviance-related responses across animals. Instead, we now emphasize the within-animal temporal ordering between AC and PFC. We also explicitly state that the later responses in Monkey C indicate inter-animal variability, which may reflect differences in electrode placement within auditory cortical fields, signal-to-noise ratio, cortical coverage, or individual variability in task-related neural dynamics. Given that the present dataset includes two animals, we do not overinterpret the precise origin of this latency difference.

Thus, the revised conclusion is more cautious: AC responses preceded PFC responses within each monkey, but the absolute timing of deviance-related responses varied across animals.

**Revised text in the manuscript**

“However, absolute latency distributions varied across animals, with Monkey C exhibiting generally later and more variable response onsets, including within AC. Accordingly, we interpret these results as evidence for a relative AC-before-PFC temporal sequence within each animal, rather than for a fixed absolute latency of deviance detection across animals. In contrast, maximum F-values among these significant channels did not differ significantly between regions (Fig. 4D; monkey X: |Z| = 1.19, p = 0.24, r = 0.12; monkey C: |Z| = 1.57, p = 0.12, r = 0.22). Sorting channels by first significant time further confirmed earlier deviance-related responses in AC (Supplementary Figs. 6A, B). The channel-by-time F-value maps also illustrated inter-animal variability in the timing and distribution of deviance-related responses.” (P.12, Lines 293–302)

“The absolute timing of these deviance-related responses varied across animals, particularly in Monkey C, indicating that the latency of Eco-defined prediction-error signals may depend on individual differences in electrode coverage, auditory-field sampling, signal-to-noise ratio, or task-related neural dynamics. Thus, our conclusion concerns the relative temporal ordering between AC and PFC within animals, rather than a fixed latency value across individuals.” (P.22, Lines 631–636)

Fig. 5 and corresponding text: I wonder if the emergence of gamma coupling in this case is only due to the evoked responses to the deviants in both AC and PFC being temporally steeper (i.e. faster rise time than responses to standards). This could change the interpretation of the results i.e. instead of gamma signals indicating a sort of fast underlying oscillation of membrane potentials, gamma inter-areal coupling could just indicate a rapid strong activation in most neurons in both areas driven by a common sensory input as opposed to bottom up or top-down functional connectivity. Again, it would help here to have a clear idea of the connectivity of the circuit.

**Our Response**

We thank you for this important and insightful comment. We agree that the gamma-band effects in Fig. 5 require a more cautious interpretation. A temporally sharp deviant-evoked response in both AC and PFC could contribute power at higher frequencies and may appear as gamma-band coupling in ECoG-based analyses, especially if both regions are driven by common sensory-related input. Therefore, we agree that the gamma-band GC effects should not be interpreted as direct evidence for fast membrane-potential oscillations, spiking-related gamma-band coupling, or definitive bottom-up/top-down cortico-cortical transmission.

In the revised manuscript, we have addressed this issue in three ways.

First, we clarified the preprocessing step used for the GC analysis. Specifically, condition-specific ERPs were subtracted from individual trials before GC estimation to reduce phase-locked evoked components associated with stimulus onset and offset. This step reduces the possibility that GC effects are driven solely by the average evoked waveform. However, we fully agree that this procedure does not completely exclude contributions from temporally sharp induced responses, common sensory drive, or shared inputs to AC and PFC. We have therefore made this limitation explicit in the Discussion.

Second, we softened the interpretation of the gamma-band results in the Results and Discussion. We now describe the gamma effect as an ECoG-level conventional gamma-band (30–70 Hz) GC-defined interaction during deviant processing, rather than as evidence for spiking-related high-gamma burst coupling or fast membrane-potential oscillations. We also clarify that our gamma analysis was performed in the conventional gamma range used in this manuscript (30–70 Hz), not as a dedicated high-gamma burst analysis. We did not specifically test the lagged co-occurrence of high-gamma amplitude bursts between AC and PFC. Addressing that question would require additional analyses focused on high-gamma amplitude envelopes, burst timing, and lagged co-occurrence, ideally combined with laminar or spike-field recordings.

Third, as described in our response to your previous GC comment, we expanded the anatomical and methodological discussion of AC–PFC connectivity. We now explicitly acknowledge that the observed GC patterns may be influenced by common input, indirect pathways, signal mixing, signal-to-noise differences, and temporally aligned evoked or induced responses. These revisions make the interpretation of Fig. 5 more cautious while preserving the central observation that deviant processing is associated with frequency-specific, behaviorally modulated AC–PFC network dynamics.

**Revised text in the manuscript**

“Across frequency bands, deviant stimuli elicited significant GC-defined directed interactions from delta to gamma ranges in both directions and across both monkeys. Temporal correlation matrices of differential GC maps across frequency-ratio pairs (1.04 vs. 1.06, 1.04 vs. 1.08, 1.06 vs. 1.08) further showed consistent AC→PFC patterns in the theta/alpha (4–14 Hz) and conventional gamma (30–70 Hz) bands, while PFC→AC patterns predominated in the theta (4–8 Hz) range. These effects were reproduced in the second monkey, consistent with frequency-specific, bidirectional ECoG-level coordination between auditory and prefrontal cortices during deviant detection.” (P.15, Lines 355–363)

“For Granger causality (GC) analyses, we applied an additional preprocessing step to the re-referenced single-trial data. The average event-related potential (ERP) was subtracted from individual trials to reduce phase-locked evoked components associated with stimulus onsets and offsets. Importantly, ERP subtraction was performed separately within each analysis condition rather than across pooled conditions.” (P.27–28, Lines 864–868. Methods)

“This condition-specific ERP-subtraction procedure reduced the contribution of stereotyped evoked waveforms to GC estimates and allowed the analysis to focus more strongly on non-phase-locked activity and trial-by-trial functional interactions between cortical regions.” (P.28, Lines 873–876. Methods)

“Although the GC analyses revealed frequency-specific directed interactions between AC and PFC, these findings warrant cautious interpretation. Granger causality quantifies whether past activity in one signal improves prediction of future activity in another signal, but it does not establish direct anatomical causality. In ECoG recordings, GC can be influenced by common input, indirect polysynaptic pathways, signal mixing, differences in signal-to-noise ratio, local spectral magnitude or power differences, and temporally aligned evoked or induced responses.” (P.22, Lines 655–661)

“We also observed conventional gamma-band (30–70 Hz) AC→PFC GC-defined interactions during deviant processing. However, these effects should not be interpreted as direct evidence for spiking-related gamma-band burst coupling or for a fast membrane-potential oscillation. Although condition-specific ERPs were subtracted from individual trials before GC analysis to reduce phase-locked evoked components, gamma-band GC could still be influenced by temporally sharp induced responses, common sensory drive, or shared inputs to AC and PFC. The present analyses were performed at the ECoG level and did not specifically test lagged co-occurrence of gamma-band amplitude bursts between AC and PFC. Future work combining laminar recordings, spike-field analyses, and event-based gamma coupling analyses will be required to determine whether the ECoG-level gamma interactions observed here reflect coordinated local spiking activity, temporally sharp population activation, or other forms of fast population-level activity.” (P.23, Lines 686–697)

Fig 6. The results on contextual modulation are very nice and make total sense considering the proposed functions of these two areas. I'm not sure I see this in the figure, but I would have liked to see a quantification of how much repetition enhancement /suppression there was in the passive vs. the active condition. I would assume the ratio suppression/enhancement shifts between these two conditions? Could you quantify this?

**Our Response**

We thank you for this helpful suggestion. We agree that the original Fig. 6 quantified the change in the magnitude of the 2-Hz slope between active and passive conditions, but did not explicitly show how the balance between repetition enhancement and repetition suppression varied across behavioral states.

In the revised manuscript, we therefore added a direct quantification of the proportion of channels showing repetition enhancement versus repetition suppression in each cortical area and behavioral condition. For each channel, we used the same 2-Hz-band magnitude slope calculated in Fig. 6B–C. Channels with positive slopes were classified as showing repetition enhancement, whereas channels with negative slopes were classified as showing repetition suppression. We then compared the enhancement/suppression balance between active and passive conditions separately for AC and PFC using exact McNemar’s tests.

This additional analysis showed a numerically higher proportion of enhancement channels during active behavior, particularly in PFC, although the categorical enhancement/suppression balance did not differ significantly between behavioral states. In AC, enhancement channels accounted for 49.3% of channels during active behavior and 43.8% during passive listening; in PFC, enhancement channels accounted for 75.8% during active behavior and 67.2% of channels during passive listening. We have added this quantification as a new panel in Fig. 6D and included a description of these results in the Results, Methods, Statistical analysis, and Figure legend sections.

This revision directly addresses your point and clarifies that behavioral engagement robustly influences the magnitude of repetition-related low-frequency activity, especially in PFC, while the categorical balance between repetition enhancement and repetition suppression shows only a modest, non-significant shift.

**Revised text in the manuscript**

“We further quantified the balance between repetition enhancement and repetition suppression by classifying each channel according to the sign of the 2-Hz-band magnitude slope. Channels with positive slopes were defined as showing repetition enhancement, whereas channels with negative slopes were defined as showing repetition suppression. This analysis showed a numerically higher proportion of enhancement channels during active behavior, particularly in PFC, although the categorical enhancement/suppression balance did not differ significantly between behavioral states. In AC, enhancement channels accounted for 49.3% during active behavior and 43.8% during passive listening; in PFC, enhancement channels accounted for 75.8% during active behavior and 67.2% during passive listening. Thus, behavioral engagement increased the magnitude of repetition-related low-frequency modulation, especially in PFC, while producing only a modest, non-significant change in the proportion of channels classified as repetition enhancement versus suppression.” (P.15, Lines 382–394)

“For the categorical analysis of repetition enhancement and repetition suppression, channels were classified according to the sign of the repetition-rate magnitude slope. Positive slopes were classified as repetition enhancement, whereas negative slopes were classified as repetition suppression. Because the same channels were compared between behavioral and passive conditions, changes in enhancement/suppression classification were assessed using exact McNemar tests separately for AC and PFC.” (P.31, Lines 1036–1041. Methods)

“(**D**) Proportions of channels showing repetition enhancement or repetition suppression under behavioral and passive conditions. Channels were classified according to the sign of the 1.8–2.2 Hz magnitude slope. Positive and negative slopes were classified as repetition enhancement and suppression, respectively. Stacked bars show the percentages of enhancement and suppression channels pooled across monkeys. p-values shown above the bars indicate exact McNemar tests comparing behavioral and passive conditions. Enh., enhancement; Sup., suppression.” (P.17, Lines 407–413. Figure 6 legend)

L512. I generally agree with this interpretation but the evidence of top-down modulation here is not necessarily overwhelming and as mentioned in my previous comments there might be alternative explanations (i.e. common activity arriving to the 2 areas either in sync or with a certain lag). This is an issue that has been largely discussed in the context of connectivity metrics such as coherence (see here: 10.1016/j.neuron.2021.09.037). I would recommend to check this literature.

**Our Response**

We thank you for this important suggestion. We agree that the evidence for top-down modulation should be presented more cautiously. In the original wording, our interpretation could be interpreted as implying that the observed PFC→AC Granger causality necessarily reflects direct top-down modulation from PFC to AC. However, as you point out, field-potential connectivity metrics can also be influenced by common input, shared sensory drive, changes in local oscillatory power, or activity reaching both regions with a temporal offset.

We have therefore revised the relevant Discussion paragraph to avoid overclaiming direct top-down modulation. Specifically, we now describe the result as stronger GC-defined PFC→AC interaction during active behavior, rather than as direct evidence that PFC feedback amplifies sensory predictions. We also added an explicit caveat that this pattern is consistent with top-down modulation but could also arise from shared inputs, behavior-dependent changes in local power, or common activity with temporal offsets.

Following your suggestion, we have also cited Schneider et al. (Neuron, 2021). This work showed that interareal field-potential coherence can depend on anatomical connectivity and local oscillatory power, and therefore does not necessarily imply direct oscillatory entrainment or direct functional communication. This literature is highly relevant to our interpretation because it reinforces the need to treat field-potential connectivity metrics, including our GC-defined interactions, as evidence for behaviorally modulated network dynamics rather than as proof of direct cortico-cortical causal signaling.

Together with the broader GC limitation paragraph added elsewhere in the Discussion, this revision makes our interpretation more conservative and better aligned with the known limitations of field-potential connectivity analyses.

**Revised text in the manuscript**

“Task engagement strongly modulated the strength and direction of functional coupling between AC and PFC. During active performance, GC-defined PFC→AC interactions were stronger than during passive listening (Supplementary Fig. 10), mirroring the higher prevalence of repetition enhancement in PFC under behaviorally relevant conditions. This pattern is consistent with the possibility that prefrontal feedback contributes to auditory predictions when sustained attention to regularities is required. However, this interpretation should be treated cautiously, because field-potential connectivity metrics can also be shaped by common input, behavior-dependent changes in local oscillatory power, or activity arriving at AC and PFC with a temporal lag. Recent work on interareal coherence has shown that long-range field-potential coupling can arise from anatomical connectivity and oscillatory power, without necessarily implying direct oscillatory entrainment or direct causal communication.” (P.21, Lines 598–608)

“Although the GC analyses revealed frequency-specific directed interactions between AC and PFC, these findings warrant cautious interpretation. Granger causality quantifies whether past activity in one signal improves prediction of future activity in another signal, but it does not establish direct anatomical causality. In ECoG recordings, GC can be influenced by common input, indirect polysynaptic pathways, signal mixing, differences in signal-to-noise ratio, local spectral magnitude or power differences, and temporally aligned evoked or induced responses. In addition, because some analyses summarized GC estimates across many electrode pairs or channel maps, the resulting GC estimates should be interpreted as array-level directed interactions rather than precise communication between specific neuronal populations. Thus, the present data leave open the possibility that part of the AC–PFC coupling reflects shared sensory drive or common input to both regions rather than direct cortico-cortical transmission. Our results therefore identify behaviorally modulated, frequency-specific directed ECoG-level interactions within an anatomically connected auditory–prefrontal network, rather than proving direct monosynaptic feedforward or feedback signaling.” (P.22–23, Lines 655–669)

Literature: The literature cited is highly macaque/human specific which is somewhat disappointing given that PLOS Bio is a general journal. People have looked at connectivity between frontal and auditory areas in other animals, here are some examples: bats (work from my own lab i.e., 10.3389/fnsys.2020.00014, https://doi.org/10.1038/s41467-022-31230-6), rodents (https://doi.org/10.1016/j.cub.2024.06.036) and marmosets (10.1016/j.cub.2025.03.077). No need to cite these papers if they are of no use, but they could really help the inter-specific interpretations presented here.

**Our Response**

We thank you for this very helpful suggestion. We agree that the previous version of the manuscript placed too much emphasis on macaque and human studies, which limited the broader comparative relevance of the work for a general biology audience. We also agree that studies in bats, rodents, and marmosets provide important context for interpreting frontal–auditory interactions across species.

In the revised manuscript, we have expanded the Discussion to include this cross-species literature. Specifically, we now cite work in bats showing fronto-temporal coupling between the frontal auditory field and auditory cortex during spontaneous activity and auditory processing, as well as behavior-dependent reversals of directed functional interactions in a frontal–auditory cortical vocalization network. These bat studies are particularly relevant because they demonstrate dynamic frontal–auditory interactions in a highly vocal mammalian model. We also added a recent study in rodents showing that the orbitofrontal cortex modulates auditory cortical sensitivity and sound perception in Mongolian gerbils, and a marmoset study demonstrating frontal–auditory cortical interactions and sensory prediction during vocal production. Together, these studies strengthen the cross-species interpretation that frontal–auditory coordination may be a conserved circuit motif for linking auditory processing with behavioral context.

We have revised the relevant Discussion paragraph accordingly. This revision broadens the manuscript beyond the macaque and human literature and makes the comparative significance of our findings clearer for PLOS Biology readers.

**Revised text in the manuscript**

“More broadly, this interpretation is also consistent with cross-species evidence that frontal–auditory coupling is not unique to macaques or humans. In bats, simultaneous recordings from frontal auditory field and auditory cortex have revealed robust fronto-temporal coupling during spontaneous activity and auditory processing, as well as behavior-dependent reversals of directed functional interactions between frontal and auditory cortices during vocal behavior. In rodents, orbitofrontal cortex modulates auditory cortical sensitivity and sound perception, supporting the idea that frontal regions can shape auditory cortical processing according to behavioral context. In marmosets, frontal–auditory cortical interactions during vocal production have been linked to sensory prediction and feedback-dependent control. Together, these studies suggest that frontal–auditory coordination may represent an evolutionarily conserved circuit motif for linking auditory processing with behavioral context.” (P.21, Lines 575–586)

Reviewer: Julio Hechavarria (I always sign reviews and encourage everyone to do the same)

**Our Response**

We thank Dr. Hechavarria for his careful, constructive, and transparent review of our manuscript. We greatly appreciate his positive assessment of the study and his detailed suggestions. These comments have helped us clarify the anatomical context of auditory–prefrontal interactions, soften the interpretation of Granger causality analyses, broaden our discussion of cross-species literature, and improve the presentation of behavioral-state modulation.

Reviewer #2

This paper represents a valuable study using macaque monkeys. While there are many animal studies on the MMN, few have examined the relationship between the auditory cortex and the prefrontal cortex. Furthermore, studies comparing conventional MMN research—in which subjects passively listen to tones—with studies conducted in the active state are expected to have a significant impact on the mechanisms underlying the generation of predictions and prediction errors. Therefore, I believe this study will be appreciated by a wide range of neuroscience readers. I have several comments that need to be addressed before I can recommend this paper for publication in this Journal.

**Our Response**

We thank you for this positive and encouraging assessment of our study. We are grateful that you recognize the value of simultaneous recordings from auditory and prefrontal cortices in macaques, and the importance of comparing active novelty detection with passive listening. We agree that this active–passive comparison is central for understanding how prediction and prediction-error signals are shaped by behavioral engagement.

In the revised manuscript, we have made several changes to strengthen this aspect of the study. First, we clarified throughout the Results that the main prediction and prediction-error analyses were performed under active behavioral conditions, and that the final Results section directly compares these active data with passive listening. Second, we added further quantification of how behavioral engagement shifts the balance between repetition enhancement and repetition suppression across AC and PFC. Third, following the concerns raised across the reviews, we have adopted a more cautious interpretation of Granger causality, emphasizing GC-defined directed interactions rather than direct causal transmission. Finally, we broadened the Discussion by adding anatomical, methodological, and cross-species context for interpreting frontal–auditory interactions.

These revisions preserve the central conclusion of the study while making the manuscript clearer, more balanced, and more accessible to a broad neuroscience readership.

1.	Supplementary figure 1 shows that many electrode contacts were above LS and below STS. These are somatosensory, motor and visual areas. Therefore, the authors should not use all 64 channels, at least for the AC array. Also, I cannot get a sense of where the AC arrays were located in the rostral-caudal direction. Can you describe where the central sulcus is? Furthermore, the waveforms on the monitor look like spikes which presumably were not recorded and should be replaced with waveforms typical of ECoG.

**Our Response**

We thank you for this important anatomical and methodological comment. We agree that it is not appropriate to treat all 64 contacts of the temporal array as auditory cortex simply based on array placement. In the revised manuscript, we have therefore adopted your suggestion and redefined the AC channels using a more conservative anatomical and functional criterion.

Specifically, AC channels were defined as temporal-array contacts located within the region between the lateral sulcus and superior temporal sulcus, together with immediately adjacent contacts close to these sulci that showed significant auditory responses. Contacts clearly outside this anatomically plausible auditory cortical region or without significant auditory responsiveness were excluded from the AC analyses. This definition was intended to include auditory cortical contacts and adjacent auditory-responsive temporal contacts while avoiding contacts likely located in non-auditory somatosensory, motor, or visual regions.

We then repeated all analyses involving AC using this redefined AC channel set, including repetition-related modulation, deviance-related responses, AC–PFC Granger causality analyses, behavioral-context comparisons, and all related topographic figures. The revised figures and statistics are now based on this anatomically and functionally defined AC channel set rather than all 64 temporal-array contacts. We have also revised the Methods and figure legends to describe this channel-selection procedure explicitly.

In addition, we revised the anatomical presentation in Supplementary Figs. 1–2. Supplementary Fig. 1 now shows the implantation sites with visible sulcal landmarks and orientation labels. The central sulcus was not clearly visible in the surgical photographs and was therefore not labeled, as is now stated explicitly in the legend. Supplementary Fig. 2 shows the sound-evoked responses used to define the AC channels, with the included AC contacts indicated on the temporal-array layout. This allows readers to see both the anatomical location of the temporal array and the functional criterion used to define AC channel set and analyses.

Finally, we agree that the original schematic neural traces could be misleading because, without a temporal reference, a zoomed ECoG waveform may visually resemble spike activity. We therefore replaced the spike-like schematic traces with representative ECoG-like field-potential waveforms and added a temporal scale bar to make clear that the signals are ECoG field potentials rather than spikes.

These revisions improve the anatomical precision of the AC definition and ensure that the analyses and figures are based on channels corresponding to auditory cortex and adjacent auditory-responsive temporal contacts.

**Revised text in the manuscript**

“Neural activity was simultaneously recorded from two 64-channel ECoG arrays implanted over the temporal auditory region and sampled lateral prefrontal cortex. Supplementary Fig. 1 shows electrode coverage and visible anatomical landmarks, including the superior temporal sulcus and lateral sulcus. Because some temporal-array contacts were located outside the likely auditory cortical territory, AC analyses were restricted to channels located within the lateral sulcus–superior temporal sulcus region and to adjacent contacts showing significant auditory responses.” (P.5, Lines 150–157)

“The temporal array was positioned to broadly cover the auditory cortical region and adjacent temporal cortex around the lateral sulcus and superior temporal sulcus. Because not all temporal-array contacts could be assumed to belong to auditory cortex based on array placement alone, AC channels were defined using combined anatomical and functional criteria. Specifically, contacts located within the region between the lateral sulcus and superior temporal sulcus were included, and contacts immediately adjacent to these sulci were also included if they showed significant sound-evoked responses. Contacts clearly outside this anatomically plausible auditory cortical region and without significant auditory responsiveness were excluded from AC analyses. Thus, all analyses involving AC were performed using this anatomically and functionally defined AC channel set.” (P.25–26, Lines 761–770)

“The prefrontal array was positioned over the lateral prefrontal cortex. Thus, throughout the manuscript, ‘PFC’ refers to the sampled lateral PFC region rather than the entire prefrontal cortex. We did not further subdivide PFC contacts into dorsolateral PFC and ventrolateral PFC, because the implanted array covered only part of the exposed lateral PFC surface, while the anatomical boundaries between dorsal and ventral prefrontal subdivisions depend partly on sulcal banks and folded cortical surfaces that cannot be resolved unambiguously from the surface ECoG layout.” (P.26, Lines 771–778)

“**Supplementary Figure 1. ECoG array implantation sites.** For each region, the left image shows the cortical surface before implantation, the middle image shows the array after implantation, and the right schematic shows electrode positions relative to visible sulcal landmarks. Top, AC; bottom, PFC. Same as A, for monkey C. The central sulcus was not clearly visible in the surgical photographs and was therefore not labeled. Scale bars, 2.5 mm. AC, auditory cortex; PFC, lateral prefrontal cortex; asl, lower limb of the arcuate sulcus; asu, upper limb of the arcuate sulcus; ps, principal sulcus; ls, lateral sulcus; sts, superior temporal sulcus; C, caudal; V, ventral.” (Supplementary Figure 1 legend)

“**Supplementary Figure 2. Sound-evoked responses used to define AC channels.** (**A**) Event-related potentials aligned to the onset of the first standard sound in the temporal array of monkey X. Traces show responses within 0–300 ms after sound onset for each channel. Purple box indicates channels included in AC analyses. The red and blue boxes indicate the example AC channels showing repetition enhancement and repetition suppression in Fig. 2, respectively. Right, topographic map of sound-evoked response magnitude, quantified as ΔRMS = RMS(0–300 ms) - RMS(-200–0 ms). Open circles indicate channels with significant positive ΔRMS (one-tailed one-sample *t*-test, *p* < 0.05, FDR-corrected). (B) Same as (A), for monkey C.” (Supplementary Figure 2 legend)

2.	Figure 2A-2C show 10 trials. Did you remove the trials that only had 7 to 9 repetitions? Where are channels AC#40, AC#27 and PFC#50? It would be insightful to describe it in an inset. Isn't the vertical axis for figures 2A-2C "Hz" instead of "KHz"? It would be better to adjust the horizontal scale of 2G to match that of 2A-2C. 2H: which ones are statistically significant individually.

**Our Response**

We thank you for these helpful comments on Fig. 2. We agree that the original figure and legend did not describe the treatment of trials with different numbers of standard repetitions with sufficient clarity.

First, Fig. 2A–C do not show 10 trials; they show responses aligned by standard-stimulus presentation order. We did not remove trials with only 7–9 repetitions. Instead, each presentation order was calculated using all trials that contained that order. Thus, trials with 7 standards contributed to orders 1–7, trials with 8 standards contributed to orders 1–8, and so on. Later orders were therefore based only on trials that contained those later repetitions. We have clarified this point in the Methods.

Second, we agree that the anatomical locations of the example channels AC#40, AC#27, and PFC#50 should be indicated more clearly. Rather than adding an inset to Fig. 2, we marked these example channels in the Fig. 2I topographic maps, where colored arrows indicate the three example channels shown in Fig. 2G. In addition, the example AC channels showing repetition suppression and enhancement are also highlighted in Supplementary Fig. 2, which shows sound-evoked responses used to define the included AC channels. This provides clearer anatomical context for the example channels displayed in Fig. 2A–C without overloading Fig. 2.

Third, you are correct that the vertical frequency axis in Fig. 2A–C should be labeled in Hz, not kHz. We have corrected this error.

Fourth, we adjusted the horizontal scale of Fig. 2G to better match that of Fig. 2A–C and aligned the sound-onset markers across panels.

Finally, we added channel-wise topographic maps of 1.8–2.2 Hz magnitude slopes in Fig. 2I and assessed individual channel significance using a bootstrap procedure. Channels with significant positive slopes were classified as showing repetition enhancement, whereas channels with significant negative slopes were classified as showing repetition suppression. We have clarified the corresponding statistical procedure in the Methods.

Together, these revisions improve the clarity of Fig. 2 and make the treatment of variable repetition numbers, the anatomical locations of the example channels, axis labeling, horizontal scaling, and channel-level significance explicit.

**Revised text in the manuscript**

“To estimate temporal changes in repetition-related activity, the mean spectral magnitude at the repetition-rate was fitted across the sequence of standard sounds, specifically from the 5th to the 9th complex tone. For analyses aligned by standard-stimulus presentation order, each order was calculated using all trials that contained that order. Thus, trials with 7, 8, 9, or 10 standards all contributed to early standard orders, whereas only trials containing later repetitions contributed to the corresponding later orders.” (P.29, Lines 919–923)

“For the channel-wise slope maps in Fig. 2I and Supplementary Fig. 3F, the significance of the repetition-rate magnitude slope was assessed using a bootstrap procedure. Trials were first grouped according to the number of standards preceding the final target sound. Within each standard-number group, trials were resampled with replacement, and the corresponding trial-averaged response was recomputed. The resampled responses from different standard-number groups were then combined using a weighted average according to the number of trials in each group, and the repetition-rate magnitude slope was recalculated. This procedure was repeated 1,000 times for each channel, yielding a bootstrap distribution of slope estimates. A two-tailed bootstrap p-value was estimated as 2 × min[P(slope ≤ 0), P(slope ≥ 0)], and channels with p < 0.05 were considered significant.” (P.29, Lines 927–937. Methods)

“**Figure 2. Repetition-related suppression and enhancement in AC and PFC activity.** (**A**) Example AC channel (#40, monkey X) showing repetition-related suppression. Top, trial-averaged normalized response; bottom, corresponding time–frequency magnitude. Vertical dashed lines indicate standard-tone onsets, and the horizontal dashed line marks 2 Hz, corresponding to the 500-ms onset-to-onset interval. (**B-C**) Example AC (#27) and PFC (#50) channels from monkey X showing repetition-related enhancement.” (P.9, Lines 177–182. Figure 2 legend)

“(**G**) Time course of magnitude in the 1.8–2.2 Hz band for the three example channels. Vertical black dashed lines indicate the onset of sounds. Slanted dashed lines represent linear fits from 2 to 4.5 s. The endpoint at 4.5 s marks the onset of the 10th standard and was used only as the boundary of the 9th-standard response window. (**H**) Dot plots of 1.8–2.2 Hz magnitude slopes across included channels in the AC and PFC arrays of both monkeys. Each dot represents one channel. Horizontal lines indicate the median, and vertical gray bars indicate the interquartile range. Numbers indicate the channels included in each group. Topographic maps of 1.8–2.2 Hz magnitude slopes for the AC and PFC arrays in monkeys X and C. Colored arrows indicate the three example channels shown in (G).” (P.9, Lines 184–194. Figure 2 legend)

“**Supplementary Figure 2. Sound-evoked responses used to define AC channels.** (**A**) Event-related potentials aligned to the onset of the first standard sound in the temporal array of monkey X. Traces show responses within 0–300 ms after sound onset for each channel. Purple box indicates channels included in AC analyses. The red and blue boxes indicate the example AC channels showing repetition enhancement and repetition suppression in Fig. 2, respectively.” (Supplementary Figure 2 legend)

3.	Line 275. Is this 5th-10th instead of 5th-9th? The dotted line in figure 2G goes from time 2 to 4.5 which I believe corresponds to tone 5 and 10.

**Our Response**

We thank you for pointing out this potential ambiguity. The fitting window was indeed the 5th–9th standard-stimulus presentations, not the 5th–10th. With a 500-ms ISI, the 5th standard begins at 2.0 s and the 10th standard begins at 4.5 s. In our analysis, the window from 2.0 to 4.5 s corresponds to the response intervals following the 5th through 9th standards; the 10th-standard onset at 4.5 s served only as the boundary of the 9th-standard response window and was not included as separate response interval.

To avoid confusion, we have clarified this point in the Figure 2 legend.

**Revised text in the manuscript**

“To estimate temporal changes in repetition-related activity, the mean spectral magnitude at the repetition-rate was fitted across the sequence of standard sounds, specifically from the 5^th^ to the 9^th^ complex tone.” (P.29, Lines 919–920. Methods)

“Slanted dashed lines represent linear fits from 2 to 4.5 s (corresponding to orders 5 to 9, indicated by the horizontal yellow line). The endpoint at 4.5 s marks the onset of the 10th standard and was used only as the boundary of the 9th-standard response window.” (P.9, Lines 186–189. Figure 2 legend)

4.	Line. 277. The percentage of repetition suppression should be restricted to auditory regions in AC. Figure 2H shows 128 AC channels, but as I described above, not all 64 AC channels should be used as some of them cover non-auditory (somatosensory, motor and visual) regions. Moreover, are there any localization of repetition suppression, for example, are they confined to regions near the primary auditory cortex (A1).

**Our Response**

We thank you for this important point. We agree that the percentage of repetition suppression and repetition enhancement should be calculated only from contacts corresponding to auditory cortex or adjacent auditory-responsive temporal cortex, rather than from all 64 temporal-array contacts.

As described in our response to your anatomical comment, we have redefined the AC channels using combined anatomical and functional criteria. Specifically, contacts located within the region between the lateral sulcus and superior temporal sulcus were included, and contacts immediately adjacent to these sulci were also included if they showed significant sound-evoked responses. Contacts clearly outside this anatomically plausible auditory cortical region or without significant auditory responsiveness were excluded from the AC analyses.

We have now recalculated the repetition-suppression and repetition-enhancement percentages using this redefined AC channel set. Accordingly, Fig. 2H and the relevant text no longer use all 64 temporal-array contacts per monkey as AC channels. Instead, the AC distributions and percentages are based only on the anatomically and functionally selected AC contacts. All other AC-related analyses and figures have been revised accordingly using the same channel selection criteria definition.

We also examined the spatial distribution of repetition suppression and repetition enhancement within the redefined AC region. In the revised figure, Fig. 2I shows topographic maps of 1.8–2.2 Hz magnitude slopes, allowing readers to assess whether repetition suppression and enhancement show spatial clustering within the sampled auditory-region channels. However, because the present ECoG recordings provide surface-level coverage and we did not perform independent tonotopic or cytoarchitectonic mapping of A1, we avoid assigning these effects specifically to primary auditory cortex. We therefore describe the distribution as occurring within the anatomically and functionally defined AC region, rather than claiming that it is confined to A1.

These revisions ensure that the reported percentages and spatial interpretations are based on auditory-region contacts and are not influenced by contacts likely located in non-auditory cortical regions.

**Revised text in the manuscript**

“Because some temporal-array contacts were located outside the likely auditory cortical territory, AC analyses were restricted to channels located within the lateral sulcus–superior temporal sulcus region and to adjacent contacts showing significant auditory responses.” (P.5, Lines 153–157)

“Across the anatomically and functionally defined AC channels, 49.3% showed increasing 2-Hz-band magnitude over repetitions, consistent with repetition enhancement, whereas 50.7% showed decreasing 2-Hz-band magnitude, consistent with repetition suppression. In PFC, 75.8% of channels showed increasing 2-Hz-band magnitude and 24.2% showed decreasing 2-Hz-band magnitude over repetitions.” (P.9–10, Lines 225–230)

“The temporal array was positioned to broadly cover the auditory cortical region and adjacent temporal cortex around the lateral sulcus and superior temporal sulcus. Because not all temporal-array contacts could be assumed to belong to auditory cortex based on array placement alone, AC channels were defined using combined anatomical and functional criteria. Specifically, contacts located within the region between the lateral sulcus and superior temporal sulcus were included, and contacts immediately adjacent to these sulci were also included if they showed significant sound-evoked responses. Contacts clearly outside this anatomically plausible auditory cortical region and without significant auditory responsiveness were excluded from AC analyses. Thus, all analyses involving AC were performed using this anatomically and functionally defined AC channel set.” (P.25–26, Lines 761–770. Methods)

“(**H**) Dot plots of 1.8–2.2 Hz magnitude slopes across included channels in the AC and PFC arrays of both monkeys. Each dot represents one channel. Horizontal lines indicate the median, and vertical gray bars indicate the interquartile range (25th–75th percentile). Numbers indicate the channels included in each group. (**I**) Topographic maps of 1.8–2.2 Hz magnitude slopes for the AC and PFC arrays in monkeys X and C.” (P.9, Lines 189–194. Figure 2 legend)

5.	Line 287. Signal averaging should be at least restricted to auditory regions. Also, is there any difference in GC between rostral AC-PFC and caudal AC-PFC? The PFC array can be divided into VLPFC and DLPFC, and I would assume that rostral AC is more connected to VLPFC, and caudal AC to DLPFC. Furthermore, are there any differences between repetition suppression and repetition enhancement channels?

**Our Response**

We thank you for this important suggestion. We agree that AC–PFC signal averaging and GC analyses should be restricted to auditory-region AC contacts rather than all 64 temporal-array contacts. As described in our response to your anatomical comment, we have redefined the AC channels using combined anatomical and functional criteria. Contacts located within the region between the lateral sulcus and superior temporal sulcus were included, and immediately adjacent contacts were also included if they showed significant sound-evoked responses. Contacts clearly outside this anatomically plausible auditory cortical region or lacking significant auditory responsiveness were excluded from AC analyses.

We have now repeated the AC–PFC GC analyses using this redefined AC channel set. Thus, the revised GC analyses no longer average across all 64 temporal-array contacts as AC channels, but instead use only the anatomically and functionally defined auditory-region contacts. We have revised the Results, Methods, and figure legends accordingly.

Regarding possible differences between rostral AC–PFC and caudal AC–PFC interactions, we agree that this is an interesting and anatomically meaningful question. However, after restricting the AC analysis to auditory-region contacts, the remaining AC channel set occupied a limited surface-accessible cortical territory around the lateral sulcus–superior temporal sulcus territory. Moreover, much of macaque auditory cortex, especially core and belt fields, lies within or near the lateral sulcus/supratemporal plane, and the present surface ECoG recordings do not provide independent tonotopic or cytoarchitectonic localization of A1, rostral belt, caudal belt, or parabelt subdivisions. For this reason, we do not think the present dataset can support a reliable rostral-versus-caudal AC subdivision or rigorously test rostral AC–VLPFC versus caudal AC–DLPFC connectivity.

Similarly, we did not further subdivide the PFC array into VLPFC and DLPFC. The implanted array covered only part of the exposed lateral PFC surface, and the anatomical boundaries between dorsal and ventral prefrontal subdivisions depend partly on sulcal banks and folded cortical surfaces that cannot be resolved unambiguously from the surface ECoG layout. We have therefore avoided overinterpreting the data in terms of pathway-specific AC–PFC anatomical streams and now describe PFC as the sampled lateral PFC region.

We also examined repetition suppression and repetition enhancement channels within the redefined AC region. Channels were classified according to the sign of the 2-Hz repetition-related slope during standard stimulation. We now show their spatial distribution on the temporal-array layout. We further addressed the relationship between repetition-related slope and deviance-related response strength in response to Comment 8, where we report that deviance-related response strength was not robustly or systematically related to whether a channel showed repetition enhancement or repetition suppression.

Together, these revisions ensure that AC–PFC GC analyses are restricted to auditory-region contacts, while avoiding unsupported claims about fine-grained rostral–caudal AC or VLPFC/DLPFC pathway specificity.

**Revised text in the manuscript**

“We focused on the 1–3 s window of each tone block corresponding to the later repetition period beginning at the third standard tone, and averaged GC estimates across pairs between the anatomically and functionally defined AC contacts and PFC contacts (Fig. 3A). GC spectra revealed a prominent ~2 Hz peak in both directions (AC→PFC and PFC→AC), indicating bidirectional GC-defined interactions near the stimulus repetition rate.” (P.10, Lines 240–245)

“The temporal array was positioned to broadly cover the auditory cortical region and adjacent temporal cortex around the lateral sulcus and superior temporal sulcus. Because not all temporal-array contacts could be assumed to belong to auditory cortex based on array placement alone, AC channels were defined using combined anatomical and functional criteria. Specifically, contacts located within the region between the lateral sulcus and superior temporal sulcus were included, and contacts immediately adjacent to these sulci were also included if they showed significant sound-evoked responses. Contacts clearly outside this anatomically plausible auditory cortical region and without significant auditory responsiveness were excluded from AC analyses. Thus, all analyses involving AC were performed using this anatomically and functionally defined AC channel set.” (P.25–26, Lines 761–770. Methods)

“The prefrontal array was positioned over the lateral prefrontal cortex. Thus, throughout the manuscript, ‘PFC’ refers to the sampled lateral PFC region rather than the entire prefrontal cortex. We did not further subdivide PFC contacts into dorsolateral PFC (DLPFC) and ventrolateral PFC (VLPFC), because the implanted array covered only part of the exposed lateral PFC surface, while the anatomical boundaries between dorsal and ventral prefrontal subdivisions depend partly on sulcal banks and folded cortical surfaces that cannot be resolved unambiguously from the surface ECoG layout.” (P.26, Lines 771–778. Methods)

“The CSD was computed from wavelet-transformed single-trial spectra for all electrode pairs between the temporal array and the PFC array. GC estimates were therefore initially obtained for all temporal-array and PFC contacts. For subsequent population-level statistical analyses and reported AC topographic summaries, however, AC contacts were restricted to the anatomically and functionally defined AC channel set described above, whereas all PFC-array contacts were retained.” (P.29, Lines 943–952. Methods)

“To estimate average GC-defined directed interactions across cortical regions, GC maps were first computed for each electrode by averaging its GC values with all electrodes in the opposite array. For AC-centered summaries, only electrodes belonging to the anatomically and functionally defined AC channel set were entered into subsequent population-level statistics and topographic summaries. For PFC-centered summaries, all PFC-array contacts were retained.” (P.30, Lines 974–981. Methods)

“(**I**) Topographic maps of 1.8–2.2 Hz magnitude slopes for the AC and PFC arrays in monkeys X and C. Colored arrows indicate the three example channels shown in (G).” (P.9, Lines 193–194. Figure 2 legend)

6.	Figure 3 legend. Is this not aligned to the third standard sound?

**Our Response**

We thank you for pointing out this ambiguity. Figure 3 was aligned to the onset of the first standard sound, with time zero defined as the first standard onset. However, for visualization, we displayed the later standard-repetition period from 1 to 3 s after sequence onset. Because the stimuli were presented with a 500-ms ISI, this displayed window begins at the onset of the third standard sound. Thus, the data were aligned to the first standard sound, but the plotted window starts from the third standard.

To avoid confusion, we have revised the Figure 3 legend to explicitly state that the GC maps were aligned to the first standard onset but displayed from 1 to 3 s, corresponding to the later standard-repetition period beginning at the third standard presentation.

**Revised text in the manuscript**

“We focused on the 1–3 s window of each tone block corresponding to the later repetition period beginning at the third standard tone, and averaged GC estimates across pairs between the anatomically and functionally defined AC contacts and PFC contacts.” (P.10, Lines 240–243)

“Time-frequency maps of Granger Causality (GC) spectrum, aligned to the onset of the first standard sound and averaged across AC–PFC channel pairs in monkey C. For visualization, only the later standard-repetition period from 1 to 3 s after sequence onset is shown; because the ISI was 500 ms, this displayed window begins at the onset of the third standard sound.” (P.11–12, Lines 248–253. Figure 3 legend)

7.	Are there no time-frequency analyses for prediction error signals?

**Our Response**

We thank you for this helpful question. In the original manuscript, the prediction-error analysis focused mainly on time-domain evoked responses and deviant–standard statistics (Fig. 4), together with time-frequency Granger causality analyses of AC–PFC interactions during deviant processing (Fig. 5). We agree that local time-frequency analyses of prediction-error signals themselves should be shown more explicitly.

In the revised manuscript, we therefore added a new supplementary figure showing local time-frequency responses aligned to the final sound onset. Because the task included multiple deviant levels and recordings from two cortical areas, we present the results separately for the standard condition and each deviant ratio (1.02, 1.04, 1.06, and 1.08) in both the AC array and PFC. We also show deviant-minus-standard local time-frequency magnitude maps for the same example AC and PFC channels shown in Fig. 4, together with band-specific quantification of deviant-minus-standard magnitude across deviant ratios. These analyses demonstrate that deviant stimuli elicited stronger local time-frequency responses than standards, with effects generally increasing with deviant magnitude, consistent with the time-domain prediction-error responses shown in Fig. 4.

In line with the revisions made in response to Reviewer 1, we describe these effects cautiously as local ECoG-level time-frequency responses associated with deviant processing, rather than as direct evidence for specific oscillatory mechanisms or spiking-related high-gamma activity.

**Revised text in the manuscript**

“We further examined local time–frequency responses time-locked to the onset of the final sound. For each deviant ratio (1.02, 1.04, 1.06, and 1.08), local time-frequency magnitude was compared with the standard condition by computing deviant-minus-standard ΔMagnitude in AC and PFC. The same example channels shown in Figs. 4A–B exhibited deviance-related time-frequency magnitude changes after final-sound onset (Supplementary Fig. 7). Band-specific quantification further showed positive deviant-minus-standard magnitude changes across multiple frequency bands and deviant ratios in both regions, consistent with the ratio-dependent deviance-related responses shown in Fig. 4. These local time–frequency analyses further characterize deviance-related ECoG activity before turning to the GC-defined AC–PFC interaction analyses.” (P.13, Lines 315–324)

“Local time-frequency responses aligned to the final-sound onset were computed from trial-averaged ECoG responses for each target/standard frequency ratio. For each deviant ratio (*λ* = 1.02, 1.04, 1.06, and 1.08), deviant-minus-standard ΔMagnitude was calculated by subtracting the CWT magnitude of the standard condition (*λ* = 1) from that of the deviant condition. For the example maps in Supplementary Fig. 7A–B, ΔMagnitude was shown for the same AC and PFC example channels used in Fig. 4A–B. For band-specific quantification, ΔMagnitude was averaged within 0–350 ms after final-sound onset and within canonical frequency bands: δ, 0.6–4 Hz; θ, 4–8 Hz; α, 8–14 Hz; β, 14–30 Hz; γ, 30–70 Hz. Statistical significance was assessed across channels using one-sample right-tailed t-tests against zero for each frequency-band × deviant-ratio combination, and p-values were FDR-corrected across these comparisons within each area and monkey.” (P.28, Lines 900–911. Methods)

“**Supplementary Figure 7. Local time-frequency responses during deviance processing.** (**A**) Deviant-minus-standard local time-frequency magnitude maps for the same example AC channel (#38, monkey X) shown in Fig. 4A, aligned to final-target onset and shown separately for target/standard frequency ratios of 1.02, 1.04, 1.06, and 1.08. ΔMagnitude was computed by subtracting the standard condition (ratio = 1) from each deviant condition. (**B**) Same as A, for an example PFC channel (#47, monkey X). (**C**) Band-specific quantification of deviant-minus-standard time-frequency magnitude in AC for monkey X and monkey C. For each deviant ratio, ΔMagnitude was averaged within 0–350 ms after final-target onset and within canonical frequency bands. (**D**) Same as (C), for PFC. Asterisks indicate significant positive deviant-minus-standard magnitude across channels after FDR correction across frequency-band × deviant-ratio comparisons.” (Supplementary Figure 7 legend)

8.	Is there any difference in prediction error signals between repetition suppression and repetition enhancement channels?

**Our Response**

We thank you for this helpful question. To address this point, we asked whether channels showing stronger repetition enhancement or repetition suppression also differed in their deviance-related response strength. Specifically, we performed an additional channel-wise analysis for this response letter by correlating each channel’s repetition-related 2-Hz-band magnitude slope with its deviance-related maximum F-value.

Across all included channels, we did not observe a significant correlation between the 2-Hz-band magnitude slope and the maximum F-value in either AC or PFC, in either monkey (AC, Monkey C: Spearman’s ρ = -0.139, p = 0.424; AC, Monkey X: ρ = -0.032, p = 0.850; PFC, Monkey C: ρ = -0.023, p = 0.856; PFC, Monkey X: ρ = -0.051, p = 0.690; Response Fig. 1). Thus, at the population level, deviance-related response strength was not systematically related to the continuous repetition-related slope.

We also performed an exploratory analysis after separating channels by slope sign. Enhancement channels, defined by positive 2-Hz-band magnitude slopes, showed no significant correlations in any area or monkey (all p > 0.09). Among suppression channels, defined by negative slopes, several nominal negative correlations were observed (AC, Monkey C: ρ = -0.623, p = 0.009; AC, Monkey X: ρ = -0.459, p = 0.044; PFC, Monkey C: ρ = -0.591, p = 0.029; PFC, Monkey X: ρ = 0.208, p = 0.421). Because these subset analyses were exploratory, not corrected for multiple comparisons, and based on smaller channel numbers, we interpret them cautiously and do not treat them as evidence for a robust dissociation between repetition-suppression and repetition-enhancement channels.

Because this additional analysis did not reveal a robust relationship and does not change the main conclusions, we did not add it to the main manuscript, but we report the analysis here for completeness.

**Response Fig. 1. Relationship between repetition-related slope and deviance-related response strength.** Scatter plots of channel-wise 2-Hz-band magnitude slope versus maximum F-value for AC and PFC in monkeys C and X. Purple indicates AC, green indicates PFC, circles indicate Monkey C, and triangles indicate Monkey X. Each point represents one channel. Across all included channels, no significant correlations were observed between repetition-related slope and deviance-related maximum F-value in either area or monkey. Exploratory analyses restricted to positive-slope channels also showed no significant correlations. Negative-slope channels showed nominal negative correlations in several area/monkey combinations, but these subset analyses were exploratory and were not corrected for multiple comparisons. Thus, deviance-related response strength was not robustly or systematically related to whether a channel showed repetition enhancement or repetition suppression.

9.	Why is there a large difference in prediction error latency between the two monkeys? Is it related to recording location within the AC?

**Our Response**

We thank you for this important question. We agree that the difference in prediction-error latency between the two monkeys should be discussed more explicitly. The later latency observed in Monkey C, including in the AC array, indicates inter-animal variability in the timing of deviance-related ECoG responses.

Recording location within the AC array is a plausible contributing factor. The temporal array was positioned to broadly cover the auditory cortical region and adjacent auditory-related temporal cortex, but the exact sampling of auditory subfields may differ between animals. Because response latencies can vary across auditory cortical fields, including core, belt, parabelt, and adjacent auditory-related regions, differences in array position or in the distribution of strongly auditory-responsive contacts could contribute to the observed latency difference.

However, we cannot attribute the latency difference solely to recording location. Other factors may also contribute, including signal-to-noise ratio, cortical folding relative to the ECoG contacts, local response strength, trial-by-trial variability, and individual differences in task-related neural dynamics. We have therefore revised the Discussion to make this point explicit. In addition, we have improved the anatomical presentation of the array locations in Supplementary Fig. 1, which should help readers evaluate the possible contribution of recording location.

Importantly, the main conclusion does not depend on a fixed absolute latency across animals. Rather, our conclusion is that deviance-related responses showed a relative AC-before-PFC temporal ordering within each monkey, while the absolute latency varied across animals.

**Revised text in the manuscript**

“However, absolute latency distributions varied across animals, with Monkey C exhibiting generally later and more variable response onsets, including within AC. Accordingly, we interpret these results as evidence for a relative AC-before-PFC temporal sequence within each animal, rather than for a fixed absolute latency of deviance detection across animals. In contrast, maximum F-values among these significant channels did not differ significantly between regions (Fig. 4D; monkey X: |Z| = 1.19, p = 0.24, r = 0.12; monkey C: |Z| = 1.57, p = 0.12, r = 0.22). Sorting channels by first significant time further confirmed earlier deviance-related responses in AC (Supplementary Figs. 6A, B). The channel-by-time F-value maps also illustrated inter-animal variability in the timing and distribution of deviance-related responses.” (P.12, Lines 293–302)

“The absolute timing of these deviance-related responses varied across animals, particularly in Monkey C, indicating that the latency of ECoG-defined prediction-error signals may depend on individual differences in electrode coverage, auditory-field sampling, signal-to-noise ratio, or task-related neural dynamics. Thus, our conclusion concerns the relative temporal ordering between AC and PFC within animals, rather than a fixed latency value across individuals.” (P.22, Lines 631–636)

“The temporal array was positioned to broadly cover the auditory cortical region and adjacent temporal cortex around the lateral sulcus and superior temporal sulcus. Because not all temporal-array contacts could be assumed to belong to auditory cortex based on array placement alone, AC channels were defined using combined anatomical and functional criteria. Specifically, contacts located within the region between the lateral sulcus and superior temporal sulcus were included, and contacts immediately adjacent to these sulci were also included if they showed significant sound-evoked responses. Contacts clearly outside this anatomically plausible auditory cortical region and without significant auditory responsiveness were excluded from AC analyses.” (P.25–26, Lines 761–769. Methods)

10.	Figure 6B. The upper right and left panels seem to be the same. Furthermore, the line drawings of the sulcus suggest that many of the panels are from the wrong monkey.

**Our Response**

We thank you for carefully checking Fig. 6B. You are correct. The previous version of Fig. 6B contained a plotting/figure-assembly error: the result from Monkey X was inadvertently duplicated, and the corresponding result from Monkey C was missing in that panel. This also led to a mismatch between some functional maps and the monkey-specific sulcal outlines.

We have now corrected Fig. 6B by replotting the behavioral and passive maps separately for Monkey X and Monkey C from the original analysis outputs. In the revised figure, each functional map is matched with the correct monkey-specific anatomical sulcal outline. We also rechecked the other topographic figures to ensure that the functional maps and anatomical outlines correspond with the correct monkey and condition.

This correction affects only the figure presentation. The underlying analysis, statistical results, and conclusions regarding behavioral modulation of repetition-related activity remain unchanged.

11.	Figure 7 is misleading in that it shows prediction as top-down and prediction error bottom-up. The data clearly shows that both prediction and prediction error have bidirectional networks.

**Our Response**

We thank you for this important comment. We agree that the previous version of Fig. 7 oversimplified the circuit interpretation by depicting prediction mainly as a top-down PFC→AC process and prediction error mainly as a bottom-up AC→PFC process. This was not fully consistent with our data, which showed bidirectional AC–PFC interactions during both standard repetition and deviant processing.

In the revised figure, we have therefore redrawn the schematic to represent prediction and prediction-error processing as bidirectional auditory–prefrontal network interactions, rather than as strictly unidirectional top-down or bottom-up pathways. During standard repetition, AC and PFC showed bidirectional low-frequency coupling, especially around the delta/2-Hz range. During deviant processing, prediction-error-related activity was also associated with bidirectional GC-defined interactions, with relatively stronger AC→PFC effects in theta/conventional gamma bands and PFC→AC effects more prominent in lower-frequency bands. We also revised the figure legend to avoid implying that prediction and prediction error are carried exclusively in one direction.

This revised schematic better reflects the main conclusion of the manuscript: predictive processing in the macaque auditory–prefrontal circuit is recurrent and bidirectional, and is modulated by behavior.

**Revised text in the manuscript**

“GC spectra revealed a prominent ~2 Hz peak in both directions (AC→PFC and PFC→AC) indicating bidirectional GC-defined interactions near the stimulus repetition rate.” (P.10, Lines 243–245)

“To assess frequency-specific GC-defined interactions, we computed GC across the 2-Hz band (1.8–2.2 Hz) and canonical frequency bands (delta: 0.6–4 Hz; theta: 4–8 Hz; alpha: 8–14 Hz; beta: 14–30 Hz; gamma: 30–70 Hz) (Fig. 3B). In both the AC→PFC and PFC→AC directions, GC-defined interactions were strongest at 2 Hz and within the delta range, with minimal coupling at higher frequencies.” (P.12, Lines 262–266)

“Topographic GC maps corroborated these effects (Fig. 5B): bidirectional GC-defined interactions were minimal for standards but rose sharply for deviants, particularly in the AC→PFC direction, with subtraction maps showing larger deviance-related increases in this direction.” (P.15, Lines 352–355)

“**Figure 7. State-dependent auditory–prefrontal dynamics during novelty detection**. Schematic summary of the main findings. During standard repetition, prediction-/repetition-related activity (P, blue) was associated with low-frequency GC-defined interactions between AC and PFC, mainly around the ~2-Hz/delta range. These interactions were weak during passive listening and stronger during active task engagement. During deviant processing, prediction-error-/deviance-related activity (PE, red) was associated with frequency-specific bidirectional GC-defined interactions. Under passive listening, AC→PFC interactions were detectable mainly in theta/alpha bands, with limited beta-band PFC→AC patterns. Under active task engagement, AC→PFC interactions were more prominent in theta/alpha and conventional gamma bands, whereas PFC→AC interactions were more evident in the theta range. Dashed arrows indicate weak or limited GC-defined interactions, and solid arrows indicate stronger or more consistent interactions.” (P.18–19, Lines 458–469. Figure 7 legend)

12.	I would like some discussion on the difference, if any, between the ventral vs dorsal auditory streams. Data on the ventral auditory stream is limited because there were no recordings in OFC and the frontal pole.

**Our Response**

We thank you for this important suggestion. We agree that the distinction between ventral and dorsal auditory streams provides useful anatomical and functional context for interpreting AC–PFC interactions. We also agree that the present data cannot fully resolve the contributions of these two streams, especially because we did not record from orbitofrontal cortex, frontal pole, parietal cortex, or motor-related frontal regions, which are important frontal targets of anterior/ventral auditory pathways.

In the revised manuscript, we have added a discussion of this limitation. The auditory ventral stream is generally associated with sound identity, auditory object recognition, and anterior temporal-to-frontal interactions, whereas the dorsal stream is more strongly associated with spatial, sensorimotor, and action-related auditory processing. Our task used non-spatial frequency deviants and therefore primarily probed auditory feature-based novelty detection rather than spatial or vocal-motor processing. However, because our ECoG arrays broadly sampled the temporal auditory-region array and sampled lateral PFC, but not OFC, frontal pole, or parietal/motor nodes, we cannot assign the observed AC–PFC interactions specifically to ventral or dorsal auditory stream pathways.

We have therefore revised the Discussion to clarify that our results should be interpreted as auditory-region–lateral-prefrontal network dynamics during novelty detection, rather than as a complete test of dorsal versus ventral stream organization. We also note that future studies combining denser anatomical coverage across anterior/ventral auditory cortex, OFC/frontal pole, parietal cortex, and dorsal frontal/motor regions will be needed to determine how prediction and prediction-error signals are distributed across the two auditory streams.

**Revised text in the manuscript**

“A related limitation concerns the distinction between ventral and dorsal auditory streams in primates. In primates, auditory processing is often described in terms of partially segregated ventral and dorsal pathways, with the ventral stream more strongly implicated in sound identity and auditory object processing, and the dorsal stream more strongly implicated in spatial, sensorimotor, and action-related auditory processing. The present task used non-spatial frequency deviants and therefore probed auditory feature-based novelty detection rather than spatial or vocal-motor processing. However, our recordings were limited to the temporal auditory-region array and lateral PFC, without coverage of orbitofrontal cortex, frontal pole, parietal cortex, or motor-related frontal regions. Thus, although the observed AC–PFC interactions reveal behaviorally modulated auditory–prefrontal predictive dynamics, they cannot be assigned unambiguously to either the ventral or dorsal auditory stream. Future studies with broader frontal and temporal coverage will be needed to determine how prediction and prediction-error signals are distributed across ventral and dorsal auditory-stream circuits.” (P.23, Lines 670–684)

“The prefrontal array was positioned over the lateral prefrontal cortex. Thus, throughout the manuscript, ‘PFC’ refers to the sampled lateral PFC region rather than the entire prefrontal cortex. We did not further subdivide PFC contacts into dorsolateral PFC and ventrolateral PFC, because the implanted array covered only part of the exposed lateral PFC surface, while the anatomical boundaries between dorsal and ventral prefrontal subdivisions depend partly on sulcal banks and folded cortical surfaces that cannot be resolved unambiguously from the surface ECoG layout.” (P.26, Lines 771–778. Methods)

13.	What anesthesia was used?

**Our Response**

We thank you for pointing out that the anesthesia protocol was not described clearly enough. We have revised the Methods to specify the anesthesia used during surgical implantation. Monkeys were premedicated with ketamine and medetomidine, then intubated and maintained under anesthesia with 1–2% isoflurane in oxygen throughout the surgical procedure.

**Revised text in the manuscript**

“Monkeys were premedicated with ketamine (50 mg/kg) and medetomidine (0.03 mg/kg), then intubated and maintained under anesthesia with 1–2% isoflurane in oxygen during surgery. Artificial ventilation was used as needed, and body temperature was maintained at 37°C using an electric heating mat. Vital signs (oxygen saturation, heart rate, and end-tidal CO_2_) were continuously monitored to adjust anesthesia depth as required.” (P.25, Lines 741–745. Methods)

Reviewer #3

This manuscript addresses an important question and is based on a valuable dataset combining simultaneous ECoG recordings from auditory and prefrontal cortices. However, the central claims are not sufficiently supported by the current analyses, and several key methodological and conceptual issues need to be addressed.

**Our Response**

We thank you for your careful evaluation of our manuscript and for recognizing the importance of the question and the value of the simultaneous auditory–prefrontal ECoG dataset. We also appreciate your critical comments regarding the strength of the central claims, the analytical support, and the conceptual framing.

In the revised manuscript, we have substantially softened the main claims and clarified the scope of interpretation. In particular, we now emphasize that the present oddball paradigm identifies repetition-related, deviance-related, and behaviorally modulated auditory–prefrontal network dynamics, but does not by itself uniquely demonstrate predictive coding mechanisms. We therefore describe predictive coding as an interpretive framework rather than as a mechanism directly established by the current data.

We have also revised the interpretation of the ~2-Hz activity, the Granger causality analyses, and the schematic model. Specifically, we now acknowledge that the ~2-Hz signal may reflect sensory tracking, temporal alignment to the repeated acoustic stream, or stimulus-rate-related modulation, not only predictive or memory-related processing. We also describe the Granger causality results as GC-defined directed ECoG-level interactions rather than direct causal communication, feedforward transmission, or top-down feedback. In addition, Fig. 7 has been revised to show prediction-related and deviance-related activity as bidirectional auditory–prefrontal network interactions rather than strictly unidirectional top-down prediction and bottom-up prediction error processes

Together, these revisions make the manuscript more cautious and better aligned with the strength of the data, while preserving the central empirical finding that behavioral engagement modulates repetition-related, deviance-related, and auditory–prefrontal ECoG network dynamics in macaques.

The primary concern is that the manuscript interprets the results within a predictive coding framework. The standard oddball paradigm used here does not allow dissociation between predictive processing and well-known alternatives such as stimulus-specific adaptation, novelty detection, or general salience responses. As a result, interpreting repetition suppression as "prediction" and deviant responses as "prediction error" remain speculative. I suggest the authors revise (soften) their claims and discuss alternative interpretations.

**Our Response**

We thank you for this important conceptual point. We agree that the standard oddball paradigm used in the present study does not by itself uniquely dissociate predictive processing from alternative mechanisms such as stimulus-specific adaptation, novelty detection, sensory salience, frequency contrast, or task-dependent gain modulation. We also agree that interpreting repetition suppression directly as “prediction” and deviant responses directly as “prediction error” would be too strong.

In the revised manuscript, we have therefore softened the predictive-coding claims throughout the text. We now describe the observed responses more cautiously as repetition-related, deviance-related, and prediction-error-related activity, rather than treating them as direct proof of predictive coding. We also clarify that predictive coding is used as an interpretive framework that is compatible with the data, rather than a mechanism uniquely established by the present experiment.

To address this point explicitly, we added a new limitation paragraph in the Discussion. This paragraph states that repetition suppression may reflect prediction-error reduction under a predictive-coding account, but may also arise from stimulus-specific adaptation, synaptic or network-level fatigue, or reduced responsiveness to repeated acoustic input. Similarly, deviance-related responses may reflect prediction-error-related activity, but may also reflect novelty detection, sensory salience, frequency contrast, or task-dependent gain modulation. We further revised the Results section headings and Fig. 7 legend to emphasize repetition-related and deviance-related auditory–prefrontal dynamics rather than implying a strict predictive-coding mechanism.

These revisions make the interpretation more cautious while preserving the central empirical finding: behavioral engagement modulates repetition-related, deviance-related, and AC–PFC network dynamics in macaques.

**Revised text in the manuscript**

We revised the Results and Discussion headings:

“Repetition-related dynamic**s** during novelty detection in AC and PFC under behavioral conditions” (P.5, Lines 138–139)

“Deviance-related response**s** during novelty detection in AC and PFC under behavioral conditions” (P.12, Lines 276–277)

“Active behavioral states amplify repetition-related and deviance-related dynamics in AC and PFC” (P.15, Lines 365–366)

“Behavioral engagement shapes deviance-related dynamics” (P.23, Line 699)

We softened the first Discussion summary paragraph:

“In this study, we examined how auditory cortex and prefrontal ECoG networks cooperate to predict and detect sensory novelty in behaving macaques. Repetition of standard tones produced two distinct response profiles: while some AC sites showed repetition suppression, a subset of AC sites and most PFC sites exhibited repetition enhancement, dominated by low-frequency (~2 Hz) repetition-related ECoG activity (Fig. 2). Linear fits of 2-Hz-band magnitude confirmed stronger facilitation in PFC than in AC (Fig. 2H). Granger causality analyses revealed robust bidirectional GC-defined interactions between the two cortical regions, peaking near 2 Hz within the delta band, consistent with coordinated predictive signaling (Fig. 3; Supplementary Figs. 4–5). Deviant tones elicited early responses in AC and later activation in PFC (Figs. 4A–C), with comparable deviance-related response strength across regions (Fig. 4D). Deviance processing further involved frequency-specific GC-defined interactions, including AC→PFC theta/alpha and conventional gamma-band patterns and PFC→AC delta/theta patterns (Fig. 5C; Supplementary Figs. 8–9). Importantly, behavioral engagement amplified local repetition-related and deviance-related responses and shaped AC–PFC GC-defined interactions (Fig. 6; Supplementary Figs. 10–11). Together, these results reveal a distributed, state-dependent auditory–prefrontal network in which auditory-region contacts capture early sensory regularities and violations, whereas PFC activity reflects behavioral context and recurrent network modulation. These findings are consistent with a predictive-coding interpretation, while also allowing alternative explanations based on adaptation, novelty detection, salience, and task-dependent gain modulation (Fig. 7).” (P.18, Lines 435–455)

We inserted a new limitation paragraph in the Discussion:

“An important limitation is that the present oddball paradigm design cannot, by itself, distinguish predictive processing from other context-dependent mechanisms. Repetition suppression may reflect reduced prediction error under a predictive-coding account, but it may also arise from stimulus-specific adaptation, synaptic or network-level fatigue, or reduced responsiveness to repeated acoustic input. Likewise, deviance-related responses can be interpreted as prediction-error-related activity, but they may also reflect novelty detection, sensory salience, frequency contrast, or task-dependent gain modulation. We therefore use predictive coding as an interpretive framework rather than as a mechanism directly proven by the present data. The central empirical finding is that repetition-related and deviance-related ECoG responses, together with AC–PFC GC-defined interactions, are modulated by behavioral engagement.” (P.19, Lines 472–483)

We softened the Abstract wording:

“The ability to detect deviations from expected sensory input is fundamental for adaptive behavior. We recorded electrocorticographic activity from the auditory (AC) and prefrontal (PFC) cortices of behaving macaques during an auditory oddball task to probe the cortical dynamics of predictive processing. Repetition of standard stimuli evoked suppression and facilitation in AC and strong low-frequency (2 Hz) enhancement in PFC, accompanied by bidirectional delta-band GC-defined interactions indicative of a shared predictive state. Deviant stimuli triggered earlier AC responses followed by PFC activation and increased GC-defined directed interactions across theta, alpha, and conventional gamma bands. Behavioral engagement amplified both repetition-related and deviance-related ECoG responses, strengthening cortical network coordination. Together, these findings reveal behaviorally gated auditory–prefrontal dynamics that are consistent with hierarchical predictive-processing accounts, while also allowing for contributions from repetition-, novelty-, and salience-related mechanisms.” (P.2, Lines 34–45)

Similarly, the interpretation of low-frequency (~2 Hz) activity as predictive signaling is not convincing. Given that this frequency closely matches the stimulation rate, the observed effects could be explained by sensory entrainment or temporal tracking. The current analyses do not rule out this alternative. This possibility should be considered and discussed.

**Our Response**

We thank you for this important point. We agree that the ~2-Hz activity should not be interpreted as definitive evidence for predictive signaling, because this frequency closely matches the 2-Hz stimulation rate in the 500-ms ISI condition. The observed low-frequency activity could also reflect sensory tracking, temporal alignment to the repeated acoustic stream, or other stimulus-rate-related modulation.

In the revised manuscript, we have therefore softened this interpretation throughout the Results and Discussion. We now describe this effect more neutrally as 2-Hz-band magnitude, repetition-related low-frequency modulation, or 2-Hz repetition-related ECoG activity, rather than treating it as direct evidence for temporal prediction. We also added a 400-ms ISI control condition, in which the stimulation rate increased to 2.5 Hz. In this condition, the AC–PFC GC peak remained near ~2 Hz rather than shifting to the 2.5-Hz stimulation rate. This argues against a purely one-to-one copy of the external stimulation rhythm, but we explicitly state that it does not rule out sensory tracking, temporal alignment, or other sequence-related low-frequency modulation.

We have also added a dedicated Discussion caveat stating that the buildup of ~2-Hz activity and delta-band AC–PFC coupling is compatible with repetition-related predictive or memory-related processing, but may also reflect temporal tracking or stimulus-rate-related modulation. We therefore conclude that the present data do not by themselves prove that the ~2-Hz response reflects temporal prediction.

**Revised text in the manuscript**

We softened the Results description of the 2-Hz activity:

“We quantified these effects by tracking 2-Hz-band magnitude, defined as the mean magnitude within 1.8–2.2 Hz, aligned with the 2-Hz stimulation rhythm corresponding to the 500-ms ISI (Fig. 2G). Linear fits (dashed lines, Fig. 2G) across the 5^th^–9^th^ tones revealed a negative slope for the first AC site, consistent with repetition suppression, and positive slopes for the second AC and PFC sites, consistent with repetition enhancement. Across the anatomically and functionally defined AC channels, 49.3% showed increasing 2-Hz-band magnitude over repetitions, consistent with repetition enhancement, whereas 50.7% showed decreasing 2-Hz-band magnitude, consistent with repetition suppression. In PFC, 75.8% of channels showed increasing 2-Hz-band magnitude and 24.2% showed decreasing 2-Hz-band magnitude over repetitions (Fig. 2H). The average slope of 2-Hz-band magnitude was significantly steeper in PFC than in AC (*F*(1,197) = 16.66, *p* < 0.001, *η*^2^ = 0.08; two-way ANOVA), indicating stronger repetition-related low-frequency modulation in PFC.” (P.9–10, Lines 221–232)

We added a 400-ms ISI control analysis and a cautious interpretation:

“Notably, when the repetition rate was increased from 2 Hz to 2.5 Hz using a 400-ms ISI, GC peaks remained near ~2 Hz rather than shifting to the 2.5-Hz stimulation rate (Supplementary Fig. 5A). This observation suggests that low-frequency AC–PFC coupling reflects more than direct tracking of the external stimulus rhythm, although sensory tracking, temporal alignment to the repeated acoustic stream, and other sequence-related low-frequency processes may contribute to the observed effects.” (P.12, Lines 269–274)

We added a Discussion caveat about temporal tracking and stimulus-rate-related modulation:

“The buildup of ~2-Hz activity and delta-band AC–PFC coupling is compatible with repetition-related predictive or memory-related processing, while also potentially reflecting sensory tracking, temporal alignment to the repeated acoustic stream, or stimulus-rate-dependent modulation of the repeated acoustic sequence. The 400-ms ISI control condition, in which the stimulation rate increased to 2.5 Hz while the AC–PFC GC peak remained near ~2 Hz, argues against a purely one-to-one stimulus-locking account. However, temporal tracking and other sequence-related low-frequency mechanisms cannot be fully excluded. Accordingly, the present data do not by themselves establish the ~2-Hz response as a signature of temporal prediction.” (P.21, Lines 588–596)

The manuscript draws strong conclusions about directional communication and assigns functional roles (feedforward prediction error vs feedback prediction) based on Granger causality results. However, GC does not establish causal interactions and is highly sensitive to common inputs, signal mixing, and differences in signal quality. Averaging signals across large electrode populations further complicates interpretation. The current conclusions regarding hierarchical signaling are too strong given the results presented.

**Our Response**

We thank you for this important methodological and conceptual concern. We agree that Granger causality should not be interpreted as proof of direct causal communication, monosynaptic feedforward transmission, or top-down feedback signaling. We also agree that common input, signal mixing, differences in signal-to-noise ratio, local spectral magnitude or power differences, and averaging across large electrode populations can complicate the interpretation of GC-based connectivity results.

In the revised manuscript, we have therefore softened the conclusions drawn from the GC analyses. We now refer to the results as GC-defined directed ECoG-level interactions rather than direct evidence of causal communication. We also avoid assigning the observed interactions exclusively to feedforward prediction-error transmission or feedback prediction signaling. Instead, we describe them as frequency-specific, behaviorally modulated auditory–prefrontal interactions that are compatible with hierarchical predictive-processing accounts but do not establish anatomically specific causal pathways.

We have added a dedicated cautionary paragraph in the Discussion stating that GC quantifies whether past activity in one signal improves prediction of future activity in another signal, but does not establish direct anatomical causality. We explicitly note that ECoG-based GC can be influenced by common input, indirect polysynaptic pathways, signal mixing, differences in signal-to-noise ratio, local spectral magnitude or power differences, and temporally aligned evoked or induced responses. We also state that because some analyses summarized GC estimates across many electrode pairs or channel maps, these results should be interpreted as array-level directed interactions rather than precise inference about communication between specific neuronal populations.

We also revised Fig. 7 and its legend to remove the simplified scheme in which prediction was depicted as purely top-down and prediction error as purely bottom-up. The revised schematic now shows both repetition-/prediction-related and deviance-/prediction-error-related activity as bidirectional AC–PFC network interactions.

Together, these revisions make the interpretation of GC more cautious and better aligned with the strength of the data, while preserving the central empirical observation that behavioral engagement modulates frequency-specific AC–PFC ECoG interactions.

**Revised text in the manuscript**

We softened the GC interpretation in the Results:

“GC spectra revealed a prominent ~2 Hz peak in both directions (AC→PFC and PFC→AC), indicating bidirectional GC-defined interactions near the stimulus repetition rate.” (P.10, Lines 243–245)

“To assess frequency-specific GC-defined interactions, we computed GC across the 2-Hz band (1.8–2.2 Hz) and canonical frequency bands (delta: 0.6–4 Hz; theta: 4–8 Hz; alpha: 8–14 Hz; beta: 14–30 Hz; gamma: 30–70 Hz) (Fig. 3B). In both the AC→PFC and PFC→AC directions, GC-defined interactions were strongest at 2 Hz and within the delta range, with minimal coupling at higher frequencies.” (P.12, Lines 262–266)

“Topographic GC maps corroborated these effects (Fig. 5B): bidirectional GC-defined interactions were minimal for standards but rose sharply for deviants, particularly in the AC→PFC direction, with subtraction maps showing larger deviance-related increases in this direction.” (P.15, Lines 352–355)

We inserted a dedicated cautionary paragraph on GC limitations:

“Although the GC analyses revealed frequency-specific directed interactions between AC and PFC, these findings warrant cautious interpretation. Granger causality quantifies whether past activity in one signal improves prediction of future activity in another signal, but it does not establish direct anatomical causality. In ECoG recordings, GC can be influenced by common input, indirect polysynaptic pathways, signal mixing, differences in signal-to-noise ratio, local spectral magnitude or power differences, and temporally aligned evoked or induced responses. In addition, because some analyses summarized GC estimates across many electrode pairs or channel maps, the resulting GC estimates should be interpreted as array-level directed interactions rather than precise communication between specific neuronal populations. Thus, the present data leave open the possibility that part of the AC–PFC coupling reflects shared sensory drive or common input to both regions rather than direct cortico-cortical transmission. Our results therefore identify behaviorally modulated, frequency-specific directed ECoG-level interactions within an anatomically connected auditory–prefrontal network, rather than proving direct monosynaptic feedforward or feedback signaling.” (P.22–23, Lines 655–669)

We revised the schematic model to avoid a strict feedforward/feedback interpretation:

“During standard repetition, prediction-/repetition-related activity (P, blue) was associated with low-frequency GC-defined interactions between AC and PFC, mainly around the ~2-Hz/delta range. These interactions were weak during passive listening and stronger during active task engagement. During deviant processing, prediction-error-/deviance-related activity (PE, red) was associated with frequency-specific bidirectional GC-defined interactions.” (P.18, Lines 459–464. Figure 7 legend)

“Dashed arrows indicate weak or limited GC-defined interactions, and solid arrows indicate stronger or more consistent interactions.” (P.19, Lines 468–469. Figure 7 legend)

We softened the overall conclusion about directional signaling:

“Together, the data support a bidirectional model of auditory predictive processing: AC responses emerged earlier than PFC responses during deviant processing, and GC analyses revealed recurrent AC–PFC interactions across frequency bands. These findings are consistent with recurrent auditory–prefrontal coordination during deviant processing, but they do not establish direct feedforward transmission of prediction errors or direct feedback transmission of predictions.” (P.24, Lines 714–719)

Overall, while the dataset is strong, the manuscript seems to overinterpret correlational findings and applies a theoretical framework that is inferred but not directly tested.

**Our Response**

We thank you for this clear summary of the main concern. We agree that the simultaneous AC–PFC ECoG dataset is correlational and that the predictive-coding framework is inferred rather than directly tested by the present oddball paradigm. We also agree that the original manuscript overstated some mechanistic interpretations.

In the revised manuscript, we have therefore softened the central claims throughout the Abstract, Results, Discussion, and Fig. 7. We now describe the findings primarily as repetition-related, deviance-related, and behaviorally modulated auditory–prefrontal ECoG dynamics, rather than as direct evidence for predictive coding mechanisms. We explicitly state that predictive coding is used as an interpretive framework, not as a mechanism directly established by the present data.

We also revised the interpretation of Granger causality. Rather than treating GC as evidence for direct causal transmission, we now describe the results as GC-defined directed ECoG-level interactions. We added a dedicated cautionary paragraph explaining that GC can be influenced by common input, indirect pathways, signal mixing, differences in signal-to-noise ratio, local spectral magnitude or power differences, and temporally aligned evoked or induced responses. We also note that averaging across many electrode pairs or channel maps supports an array-level interpretation, not precise inference about communication between specific neuronal populations.

Finally, we revised Fig. 7 and the Discussion to avoid presenting prediction as exclusively top-down and prediction error as exclusively bottom-up. The revised schematic and text now emphasize bidirectional auditory–prefrontal interactions during both standard repetition and deviant processing. Together, these revisions make the manuscript more cautious and better aligned with the strength and limits of the data.

**Revised text in the manuscript**

“An important limitation is that the present oddball paradigm design cannot, by itself, distinguish predictive processing from other context-dependent mechanisms. Repetition suppression may reflect reduced prediction error under a predictive-coding account, but it may also arise from stimulus-specific adaptation, synaptic or network-level fatigue, or reduced responsiveness to repeated acoustic input. Likewise, deviance-related responses can be interpreted as prediction-error-related activity, but they may also reflect novelty detection, sensory salience, frequency contrast, or task-dependent gain modulation. We therefore use predictive coding as an interpretive framework rather than as a mechanism directly proven by the present data. The central empirical finding is that repetition-related and deviance-related ECoG responses, together with AC–PFC GC-defined interactions, are modulated by behavioral engagement.” (P.19, Lines 472–483)
