# Supplementary material for: Behavioral engagement and stimulus regularities coordinate auditory-prefrontal network activity
Source: PLoS Biol. 2026 Aug 26;24(8):e3003966. doi: 10.1371/journal.pbio.3003966 (PMC13537682; doi:10.1371/journal.pbio.3003966)
Supplement: Peer review history

# Editor report: Decision Letter 0

**DOI:** 10.1371/journal.pbio.3003966.r001

**Authors:** Taylor Hart, PhD

17 Mar 2026

Dear Dr Malmierca,

Thank you for submitting your manuscript entitled "Behaviorally gated hierarchical predictive coding in macaque auditory–prefrontal circuits" for consideration as a Research Article by PLOS Biology.

Your manuscript has now been evaluated by the PLOS Biology editorial staff, and by a member of the editorial board, and I am writing to let you know that we would like to send your submission out for external peer review.

However, before we can send your manuscript to reviewers, we need you to complete your submission by providing the metadata that is required for full assessment. To this end, please login to Editorial Manager where you will find the paper in the 'Submissions Needing Revisions' folder on your homepage. Please click 'Revise Submission' from the Action Links and complete all additional questions in the submission questionnaire.

Once your full submission is complete, your paper will undergo a series of checks in preparation for peer review. After your manuscript has passed the checks it will be sent out for review. To provide the metadata for your submission, please Login to Editorial Manager (https://www.editorialmanager.com/pbiology) within two working days, i.e. by Mar 19 2026 11:59PM.

If your manuscript has been previously peer-reviewed at another journal, PLOS Biology is willing to work with those reviews in order to avoid re-starting the process. Submission of the previous reviews is entirely optional and our ability to use them effectively will depend on the willingness of the previous journal to confirm the content of the reports and share the reviewer identities. Please note that we reserve the right to invite additional reviewers if we consider that additional/independent reviewers are needed, although we aim to avoid this as far as possible. In our experience, working with previous reviews does save time.

If you would like us to consider previous reviewer reports, please edit your cover letter to let us know and include the name of the journal where the work was previously considered and the manuscript ID it was given. In addition, please upload a response to the reviews as a 'Prior Peer Review' file type, which should include the reports in full and a point-by-point reply detailing how you have or plan to address the reviewers' concerns.

During the process of completing your manuscript submission, you will be invited to opt-in to posting your pre-review manuscript as a bioRxiv preprint. Visit http://journals.plos.org/plosbiology/s/preprints for full details. If you consent to posting your current manuscript as a preprint, please upload a single Preprint PDF.

Feel free to email us at plosbiology@plos.org if you have any queries relating to your submission.

Kind regards,

Taylor

Taylor Hart, PhD,

Associate Editor

PLOS Biology

thart@plos.org

# Peer review: Decision Letter 1

**DOI:** 10.1371/journal.pbio.3003966.r002

**Authors:** Taylor Hart, PhD

24 Apr 2026

Dear Dr Malmierca,

Thank you for your patience while your manuscript "Behaviorally gated hierarchical predictive coding in macaque auditory–prefrontal circuits" was peer-reviewed at PLOS Biology. It has now been evaluated by the PLOS Biology editors, an Academic Editor with relevant expertise, and by several independent reviewers.

In light of the reviews, which you will find at the end of this email, we would like to invite you to revise the work to thoroughly address the reviewers' reports.

As you will see, the reviewers expressed interest in the study, but pointed out areas where the major claims were not fully supported by the available data. The reviewers have raised several conceptual, methodological, analytical, and interpretational concerns, and also commented on limitations in the engagement with prior literature and with the presentation. In your revision, you should thoroughly address all of the reviewers' points.

Given the extent of revision needed, we cannot make a decision about publication until we have seen the revised manuscript and your response to the reviewers' comments. Your revised manuscript is likely to be sent for further evaluation by all or a subset of the reviewers.

In addition to these revisions, you will need to complete some formatting changes, which you will receive in a follow up email. A member of our team will be in touch with a set of requests shortly.

We expect to receive your revised manuscript within 3 months. Please email us (plosbiology@plos.org) if you have any questions or concerns, or would like to request an extension.

At this stage, your manuscript remains formally under active consideration at our journal; please notify us by email if you do not intend to submit a revision so that we may withdraw it.

**IMPORTANT - SUBMITTING YOUR REVISION**

Your revisions should address the specific points made by each reviewer. Please submit the following files along with your revised manuscript:

1. A 'Response to Reviewers' file - this should detail your responses to the editorial requests, present a point-by-point response to all of the reviewers' comments, and indicate the changes made to the manuscript.

*NOTE: In your point-by-point response to the reviewers, please provide the full context of each review. Do not selectively quote paragraphs or sentences to reply to. The entire set of reviewer comments should be present in full and each specific point should be responded to individually, point by point.

You should also cite any additional relevant literature that has been published since the original submission and mention any additional citations in your response.

2. In addition to a clean copy of the manuscript, please also upload a 'track-changes' version of your manuscript that specifies the edits made. This should be uploaded as a "Revised Article with Changes Highlighted" file type.

*Re-submission Checklist*

When you are ready to resubmit your revised manuscript, please refer to this re-submission checklist: https://plos.io/Biology_Checklist

To submit a revised version of your manuscript, please go to https://www.editorialmanager.com/pbiology/ and log in as an Author. Click the link labelled 'Submissions Needing Revision' where you will find your submission record.

Please make sure to read the following important policies and guidelines while preparing your revision:

*Published Peer Review*

Please note while forming your response, if your article is accepted, you may have the opportunity to make the peer review history publicly available. The record will include editor decision letters (with reviews) and your responses to reviewer comments. If eligible, we will contact you to opt in or out. Please see here for more details:

https://blogs.plos.org/plos/2019/05/plos-journals-now-open-for-published-peer-review/

*PLOS Data Policy*

Please note that as a condition of publication PLOS' data policy (http://journals.plos.org/plosbiology/s/data-availability) requires that you make available all data used to draw the conclusions arrived at in your manuscript. If you have not already done so, you must include any data used in your manuscript either in appropriate repositories, within the body of the manuscript, or as supporting information (N.B. this includes any numerical values that were used to generate graphs, histograms etc.). For an example see here: http://www.plosbiology.org/article/info%3Adoi%2F10.1371%2Fjournal.pbio.1001908#s5

*Blot and Gel Data Policy*

We require the original, uncropped and minimally adjusted images supporting all blot and gel results reported in an article's figures or Supporting Information files. We will require these files before a manuscript can be accepted so please prepare them now, if you have not already uploaded them. Please carefully read our guidelines for how to prepare and upload this data: https://journals.plos.org/plosbiology/s/figures#loc-blot-and-gel-reporting-requirements

*Protocols deposition*

To enhance the reproducibility of your results, we recommend that if applicable you deposit your laboratory protocols in protocols.io, where a protocol can be assigned its own identifier (DOI) such that it can be cited independently in the future. Additionally, PLOS ONE offers an option for publishing peer-reviewed Lab Protocol articles, which describe protocols hosted on protocols.io. Read more information on sharing protocols at https://plos.org/protocols?utm_medium=editorial-email&utm_source=authorletters&utm_campaign=protocols

Thank you again for your submission to our journal. We hope that our editorial process has been constructive thus far, and we welcome your feedback at any time. Please don't hesitate to contact us if you have any questions or comments.

Sincerely,

Taylor

Taylor Hart, PhD,

Associate Editor

PLOS Biology

thart@plos.org

REVIEWS

Reviewer #1 [Julio Hechavarria]: This is my assessment of the article entitled: Behaviorally gated hierarchical predictive coding in macaque auditory-prefrontal circuits submitted to Plos Biology by Xu et al. The article deals with the question of how unpredictable and predictable auditory events are represented in two cortical areas: the PFC and AC. These two areas likely are fundamental parts of the auditory network and as such they need to be studied simultaneously to understand how they bind information together.

In the article the authors present simultaneous ECOG recordings form frontal and AC areas in two monkeys that were either engaged in a detection task or passively listening to mixtures of predictable and unpredictable events. The results clearly show the existence of interareal coupling in the delta and low theta frequency bands of the field potentials recorded, as well as distinct (likely complementary ) representations of deviant and standard sours in the PFC-AC network. I enjoyed the paper very much and I think it is a great match for PLOS Biology as it sheds light into the neural substrates that allow the brain to process complex sound streams. While the paper is very good, I do see several points where clarification is needed, particularly regarding the interpretation of Granger Causality results and how they might be constrained by the underlying circuit anatomy. My specific comments are listed below.

Comments:

Colors in Fig. 2 G are difficult to distinguish. Consider a different color scale?

Line 280-> the word entrainment is used here to indicate evoked responses that track the acoustic stream but it could inpmly that there are temporal predictions occurring which is not clearly tested here.

GC calculations. I commend the authors on these results. The delta/theta coupling between AC and PFC seems robust and is beautifully illustrated here. It seems this coupling is evolutionarily preserved (i.e. see papers on bats). I would have liked to have a bit more context here in the sense of the anatomy of the circuit in monkeys. Are there direct bidirectional projections between the PFC and AC? In other species, the PFC receives a fast input the by-passes the canonical auditory pathway and produces latencies that are shorter than those observed in the AC. Can we rule out that the connectivity reported here is not just a consequence of both areas receiving common input as opposed to the interaction between them? Beyond the low frequency LFPs, did you observe any type of interactions in the high gamma range that is usually related to spiking? i.e. gamma bursts co-occurring in the two areas with a certain lag?

L332-334: yes but in Monkey C already occurred quite late even for the AC which could indicate high variability in the results.

Fig. 5 and corresponding text: I wonder if the emergence of gamma coupling in this case is only due to the evoked responses to the deviants in both AC and PFC being temporally steeper (i.e. faster rise time than responses to standards). This could change the interpretation of the results i.e. instead of gamma signals indicating a sort of fast underlying oscillation of membrane potentials, gamma inter-areal coupling could just indicate a rapid strong activation in most neurons in both areas driven by a common sensory input as opposed to bottom up or top-down functional connectivity. Again, it would help here to have a clear idea of the connectivity of the circuit.

Fig 6. The results on contextual modulation are very nice and make total sense considering the proposed functions of these two areas. I'm not sure I see this in the figure, but I would have liked to see a quantification of how much repetition enhancement /suppression there was in the passive vs. the active condition. I would assume the ratio suppression/enhancement shifts between these two conditions? Could you quantify this?

L512. I generally agree with this interpretation but the evidence of top-down modulation here is not necessarily overwhelming and as mentioned in my previous comments there might be alternative explanations (i.e. common activity arriving to the 2 areas either in sync or with a certain lag). This is an issue that has been largely discussed in the context of connectivity metrics such as coherence (see here: 10.1016/j.neuron.2021.09.037). I would recommend to check this literature.

Literature: The literature cited is highly macaque/human specific which is somewhat disappointing given that PLOS Bio is a general journal. People have looked at connectivity between frontal and auditory areas in other animals, here are some examples: bats (work from my own lab i.e. 10.3389/fnsys.2020.00014, https://doi.org/10.1038/s41467-022-31230-6 ), rodents (https://doi.org/10.1016/j.cub.2024.06.036) and marmosets (10.1016/j.cub.2025.03.077). No need to cite these papers if they are of no use, but they could really help the inter-specific interpretations presented here.

Reviewer: Julio Hechavarria (I always sign reviews and encourage everyone to do the same)

Reviewer #2: This paper represents a valuable study using macaque monkeys. While there are many animal studies on the MMN, few have examined the relationship between the auditory cortex and the prefrontal cortex. Furthermore, studies comparing conventional MMN research—in which subjects passively listen to tones—with studies conducted in the active state are expected to have a significant impact on the mechanisms underlying the generation of predictions and prediction errors. Therefore, I believe this study will be appreciated by a wide range of neuroscience readers. I have several comments that need to be addressed before I can recommend this paper for publication in this Journal.

1.Supplementary figure 1 shows that many electrode contacts were above LS and below STS. These are somatosensory, motor and visual areas. Therefore, the authors should not use all 64 channels, at least for the AC array. Also, I cannot get a sense of where the AC arrays were located in the rostral-caudal direction. Can you describe where the central sulcus is? Furthermore, the waveforms on the monitor look like spikes which presumably were not recorded and should be replaced with waveforms typical of ECoG.

2.Figure 2A-2C show 10 trials. Did you remove the trials that only had 7 to 9 repetitions? Where are channels AC#40, AC#27 and PFC#50? It would be insightful to describe it in an inset. Isn't the vertical axis for figures 2A-2C "Hz" instead of "KHz"? It would be better to adjust the horizontal scale of 2G to match that of 2A-2C. 2H: which ones are statistically significant individually

3.Line 275. Is this 5th-10th instead of 5th-9th? The dotted line in figure 2G goes from time 2 to 4.5 which I believe corresponds to tone 5 and 10.

4.Line. 277. The percentage of repetition suppression should be restricted to auditory regions in AC. Figure 2H shows 128 AC channels, but as I described above, not all 64 AC channels should be used as some of them cover non-auditory (somatosensory, motor and visual) regions. Moreover, are there any localization of repetition suppression, for example, are they confined to regions near the primary auditory cortex (A1).

5.Line 287. Signal averaging should be at least restricted to auditory regions. Also, is there any difference in GC between rostral AC-PFC and caudal AC-PFC? The PFC array can be divided into VLPFC and DLPFC, and I would assume that rostral AC is more connected to VLPFC, and caudal AC to DLPFC. Furthermore, are there any differences between repetition suppression and repetition enhancement channels?

6.Figure 3 legend. Is this not aligned to the third standard sound?

7.Are there no time-frequency analyses for prediction error signals?

8.Is there any difference in prediction error signals between repetition suppression and repetition enhancement channels?

9.Why is there a large difference in prediction error latency between the two monkeys? Is it related to recording location within the AC?

10.Figure 6B. The upper right and left panels seem to be the same. Furthermore, the line drawings of the sulcus suggest that many of the panels are from the wrong monkey.

11.Figure 7 is misleading in that it shows prediction as top-down and prediction error bottom-up. The data clearly shows that both prediction and prediction error have bidirectional networks.

12.I would like some discussion on the difference, if any, between the ventral vs dorsal auditory streams. Data on the ventral auditory stream is limited because there were no recordings in OFC and the frontal pole.

13.What anesthesia was used?

Reviewer #3: This manuscript addresses an important question and is based on a valuable dataset combining simultaneous ECoG recordings from auditory and prefrontal cortices. However, the central claims are not sufficiently supported by the current analyses, and several key methodological and conceptual issues need to be addressed.

The primary concern is that the manuscript interprets the results within a predictive coding framework. The standard oddball paradigm used here does not allow dissociation between predictive processing and well-known alternatives such as stimulus-specific adaptation, novelty detection, or general salience responses. As a result, interpreting repetition suppression as "prediction" and deviant responses as "prediction error" remain speculative. I suggest the authors revise (soften) their claims and discuss alternative interpretations.

Similarly, the interpretation of low-frequency (~2 Hz) activity as predictive signaling is not convincing. Given that this frequency closely matches the stimulation rate, the observed effects could be explained by sensory entrainment or temporal tracking. The current analyses do not rule out this alternative. This possibility should be considered and discussed.

The manuscript draws strong conclusions about directional communication and assigns functional roles (feedforward prediction error vs feedback prediction) based on Granger causality results. However, GC does not establish causal interactions and is highly sensitive to common inputs, signal mixing, and differences in signal quality. Averaging signals across large electrode populations further complicates interpretation. The current conclusions regarding hierarchical signaling are too strong given the results presented.

Overall, while the dataset is strong, the manuscript seems to overinterpret correlational findings and applies a theoretical framework that is inferred but not directly tested.

# Author comment: Author response to Decision Letter 2

**DOI:** 10.1371/journal.pbio.3003966.r003

8 Jun 2026

Attachment Submitted filename: Response_to_Comments_260606.docx

# Peer review: Decision Letter 2

**DOI:** 10.1371/journal.pbio.3003966.r004

**Authors:** Taylor Hart, PhD

30 Jul 2026

Dear Dr Malmierca,

Thank you for your patience while we considered your revised manuscript "Behaviorally gated hierarchical predictive coding in macaque auditory–prefrontal circuits" for publication as a Research Article at PLOS Biology. This revised version of your manuscript has been evaluated by the PLOS Biology editors, the Academic Editor and two of the original reviewers. I would also like to extend my apologies again for the slow turnaround time on this decision, which was affected by the availability of the reviewers and the editorial team.

Based on the reviews and on our Academic Editor's assessment of your revision, we are likely to accept this manuscript for publication. Please also make sure to address the following data and other policy-related requests.

IMPORTANT: Please ensure that your next revision addresses the following points:

----------------------

**Title:

We would like to modify the paper's title, both to fit our stylistic preference for an 'active' verb, and to avoid the exclusive emphasis on the predictive coding framework that was critiqued by the reviewers. I have provided a suggested alternative title. Is this acceptable to you?

'Behavioral engagement and stimulus regularities coordinate auditory-prefrontal network activity'

**Financial disclosure statement:

Please add links to the funding agencies in the Financial Disclosure statement in the manuscript details, and delete the extraneous quotation mark at the end.

**Ethics:

Please move the information about ethical protocols and approvals to a new a new, first subheading of the Materials and Methods section, titled "Ethics Statement".

**Data:

Thank you for including the underlying data in your Zenodo repository. Please confirm that the numerical data related to all of the following figure panels are available (including all replicates, and with appropriate labels). In particular, I did not see data listed for Fig. 4CD, but it is possible that the labeling needs to be updated for the revised manuscript, or that I missed this.

This applies to the following figure panels:

Figs

1C

2H

4CD

6D

S3D

Please also ensure that your Data Statement in the submission system accurately describes where your data can be found and is in final format, as it will be published as written there.

**Supplement:

As you have already uploaded the supplementary figures individually, please move the supplementary figure Legends into the main manuscript document, and then remove the additional 'Supplementary Materials' text document and the PDF also containing the figures.

**Code availability:

Thank you for providing the underlying code in GitHub. However, I could not access it through the provided link (please check if this is correct).

Moreover, because Github depositions can be readily changed or deleted, please make a permanent DOI’d copy (e.g. in Zenodo) and provide this URL in the manuscript and Data Availability Statement. Please also confirm that you have selected a license for your code and included a README document.

----------------------

As you address these items, please take this last chance to review your reference list to ensure that it is complete and correct. If you have cited papers that have been retracted, please include the rationale for doing so in the manuscript text, or remove these references and replace them with relevant current references. Any changes to the reference list should be mentioned in the cover letter that accompanies your revised manuscript.

In addition to these revisions, you may need to complete some formatting changes, which you will receive in a follow up email. A member of our team will be in touch with a set of requests shortly. If you do not receive a separate email within a few days, please assume that checks have been completed, and no additional changes are required.

We expect to receive your revised manuscript within two weeks.

To submit your revision, please go to https://www.editorialmanager.com/pbiology/ and log in as an Author. Click the link labelled 'Submissions Needing Revision' to find your submission record. Your revised submission must include the following:

- a cover letter that should detail your responses to any editorial requests, if applicable, and whether changes have been made to the reference list

- a Response to Reviewers file that provides a detailed response to the reviewers' comments (if applicable, if not applicable please do not delete your existing 'Response to Reviewers' file.)

- a track-changes file indicating any changes that you have made to the manuscript.

NOTE: If Supporting Information files are included with your article, note that these are not copyedited and will be published as they are submitted. Please ensure that these files are legible and of high quality (at least 300 dpi) in an easily accessible file format. For this reason, please be aware that any references listed in an SI file will not be indexed. For more information, see our Supporting Information guidelines:

https://journals.plos.org/plosbiology/s/supporting-information

*Published Peer Review History*

Please note that you may have the opportunity to make the peer review history publicly available. The record will include editor decision letters (with reviews) and your responses to reviewer comments. If eligible, we will contact you to opt in or out. Please see here for more details:

https://plos.org/published-peer-review-history/

*Press*

Should you, your institution's press office or the journal office choose to press release your paper, please ensure you have opted out of Early Article Posting on the submission form. We ask that you notify us as soon as possible if you or your institution is planning to press release the article.

*Protocols deposition*

To enhance the reproducibility of your results, we recommend that if applicable you deposit your laboratory protocols in protocols.io, where a protocol can be assigned its own identifier (DOI) such that it can be cited independently in the future. Additionally, PLOS ONE offers an option for publishing peer-reviewed Lab Protocol articles, which describe protocols hosted on protocols.io. Read more information on sharing protocols at https://plos.org/protocols?utm_medium=editorial-email&utm_source=authorletters&utm_campaign=protocols

Please do not hesitate to contact me should you have any questions.

Sincerely,

Taylor

Taylor Hart, PhD,

Associate Editor

thart@plos.org

PLOS Biology

REVIEWS

Reviewer #1 [Julio Hechavarria]: I thank the authors very much for their thoughtfull responses to my comments. I think the paper is really beatiful and will make a fantastic contribution to our understanding of deviance detection and fronto-autditory interactions in the mammalian brain. No further comments from my side.

Reviewer #2: I would like to express my respect to the authors. I have no further comments.

# Editor report: Decision Letter 3

**DOI:** 10.1371/journal.pbio.3003966.r005

**Authors:** Taylor Hart, PhD

7 Aug 2026

Dear Manolo,

Thank you for the submission of your revised Research Article "Behavioral engagement and stimulus regularities coordinate auditory-prefrontal network activity" for publication in PLOS Biology. On behalf of my colleagues and the Academic Editor, Jennifer Bizley, I am pleased to say that we can in principle accept your manuscript for publication, provided you address any remaining formatting and reporting issues. These will be detailed in an email you should receive within 2-3 business days from our colleagues in the journal operations team; no action is required from you until then. Please note that we will not be able to formally accept your manuscript and schedule it for publication until you have completed any requested changes.

Please take a minute to log into Editorial Manager at http://www.editorialmanager.com/pbiology/, click the "Update My Information" link at the top of the page, and update your user information to ensure an efficient production process.

PRESS

We frequently collaborate with press offices. If your institution or institutions have a press office, please notify them about your upcoming paper at this point, to enable them to help maximise its impact. If the press office is planning to promote your findings, we would be grateful if they could coordinate with biologypress@plos.org. If you have previously opted in to the early version process, we ask that you notify us immediately of any press plans so that we may opt out on your behalf.

We also ask that you take this opportunity to read our Embargo Policy regarding the discussion, promotion and media coverage of work that is yet to be published by PLOS. As your manuscript is not yet published, it is bound by the conditions of our Embargo Policy. Please be aware that this policy is in place both to ensure that any press coverage of your article is fully substantiated and to provide a direct link between such coverage and the published work. For full details of our Embargo Policy, please visit http://www.plos.org/about/media-inquiries/embargo-policy/.

Thank you again for choosing PLOS Biology for publication and supporting Open Access publishing. We look forward to publishing your study.

Sincerely,

Taylor

Taylor Hart, PhD,

Associate Editor

PLOS Biology

thart@plos.org
